# Supplementary material for: Expression of miR-34a-5p is up-regulated in human colorectal cancer and correlates with survival and clock gene PER2 expression
Source: PLoS One. 2019 Oct 28;14(10):e0224396. doi: 10.1371/journal.pone.0224396 (PMC6816564; doi:10.1371/journal.pone.0224396)
Supplement: S1 Table — (DOCX) [file pone.0224396.s001.docx]

**S1 Table:** Results from *in silico* analysis with the TargetScan database

<http://www.targetscan.org/vert_72/>.

| **miRNA** | **Position in the UTR** | **seed match** | **context++ score** | **context++ score percentile** | **weighted context++ score** | **conserved branch length** | **Pct** |
| --- | --- | --- | --- | --- | --- | --- | --- |
| **Conserved sites** | | | | | | | |
| hsa-miR-103a-3p | 87-93 | 7mer-1A | -0.01 | 18 | -0.01 | 3.746 | N/A |
| hsa-miR-107 | 87-93 | 7mer-1A | -0.01 | 18 | -0.01 | 3.746 | N/A |
| hsa-miR-491-5p | 101-107 | 7mer-1A | -0.13 | 68 | -0.13 | 3.650 | N/A |
| hsa-miR-24-3p | 162-168 | 7mer-1A | -0.1 | 79 | -0.1 | 3.887 | N/A |
| hsa-miR-149-5p | 163-170 | 8mer | -0.33 | 97 | -0.33 | 3.404 | N/A |
| hsa-miR-708-5p | 414-420 | 7mer-1A | -0.12 | 79 | -0.12 | 3.818 | N/A |
| hsa-miR-28-5p | 414-420 | 7mer-1A | -0.12 | 79 | -0.12 | 3.818 | N/A |
| hsa-miR-3139 | 414-420 | 7mer-1A | -0.08 | 70 | -0.08 | 3.818 | N/A |
| hsa-miR-138-5p | 420-426 | 7mer-1A | -0.09 | 60 | -0.09 | 5.245 | N/A |
| hsa-miR-138-5p | 676-682 | 7mer-m8 | -0.1 | 64 | -0.1 | 4.560 | N/A |
| hsa-miR-3064-5p | 679-685 | 7mer-m8 | -0.07 | 67 | -0.07 | 4.338 | N/A |
| hsa-miR-6504-5p | 679-685 | 7mer-m8 | -0.06 | 63 | -0.06 | 4.338 | N/A |
| hsa-miR-22-3p | 687-693 | 7mer-1A | -0.06 | 63 | -0.06 | 7.027 | N/A |
| hsa-miR-212-5p | 855-861 | 7mer-1A | -0.05 | 60 | -0.05 | 5.759 | N/A |
| hsa-miR-873-5p.2 | 899-906 | 8mer | -0.03 | 30 | -0.03 | 3.156 | N/A |
| hsa-miR-34c-5p | 947-954 | 8mer | -0.26 | 86 | -0.26 | 2.068 | N/A |
| hsa-miR-449b-5p | 947-954 | 8mer | -0.26 | 86 | -0.26 | 2.068 | N/A |
| **hsa-miR-34a-5p** | 947-954 | 8mer | -0.25 | 86 | -0.25 | 2.068 | N/A |
| hsa-miR-449a | 947-954 | 8mer | -0.24 | 84 | -0.24 | 2.068 | N/A |
| hsa-miR-378h | 1191-1197 | 7mer-m8 | -0.08 | 65 | -0.08 | 3.813 | N/A |
| hsa-miR-378e | 1191-1197 | 7mer-m8 | -0.05 | 56 | -0.05 | 3.813 | N/A |
| hsa-miR-422a | 1191-1197 | 7mer-m8 | -0.05 | 55 | -0.05 | 3.813 | N/A |
| hsa-miR-378d | 1191-1197 | 7mer-m8 | -0.05 | 55 | -0.05 | 3.813 | N/A |
| hsa-miR-378c | 1191-1197 | 7mer-m8 | -0.05 | 55 | -0.05 | 3.813 | N/A |
| hsa-miR-378a-3p | 1191-1197 | 7mer-m8 | -0.05 | 55 | -0.05 | 3.813 | N/A |
| hsa-miR-378i | 1191-1197 | 7mer-m8 | -0.05 | 55 | -0.05 | 3.813 | N/A |
| hsa-miR-378b | 1191-1197 | 7mer-m8 | -0.03 | 42 | -0.03 | 3.813 | N/A |
| hsa-miR-378f | 1191-1197 | 7mer-m8 | -0.03 | 42 | -0.03 | 3.813 | N/A |
| hsa-miR-138-5p | 1973-1979 | 7mer-1A | -0.16 | 75 | -0.16 | 6.503 | N/A |
| hsa-miR-30d-5p | 2302-2308 | 7mer-m8 | -0.02 | 16 | -0.02 | 4.159 | N/A |
| hsa-miR-30a-5p | 2302-2308 | 7mer-m8 | -0.02 | 16 | -0.02 | 4.159 | N/A |
| hsa-miR-30e-5p | 2302-2308 | 7mer-m8 | -0.02 | 15 | -0.02 | 4.159 | N/A |
| hsa-miR-30c-5p | 2302-2308 | 7mer-m8 | -0.02 | 15 | -0.02 | 4.159 | N/A |
| hsa-miR-30b-5p | 2302-2308 | 7mer-m8 | -0.02 | 15 | -0.02 | 4.159 | N/A |
| hsa-miR-27b-3p | 2307-2314 | 8mer | -0.17 | 90 | -0.17 | 3.306 | N/A |
| hsa-miR-27a-3p | 2307-2314 | 8mer | -0.14 | 87 | -0.14 | 3.306 | N/A |
| hsa-miR-3681-3p | 2307-2313 | 7mer-1A | -0.06 | 66 | -0.06 | 5.357 | N/A |
| hsa-miR-128-3p | 2307-2313 | 7mer-1A | -0.03 | 59 | -0.03 | 5.357 | N/A |
| hsa-miR-216a-3p | 2307-2313 | 7mer-1A | -0.03 | 58 | -0.03 | 5.357 | N/A |
| hsa-miR-9-5p | 2390-2397 | 8mer | -0.03 | 63 | -0.03 | 2.233 | N/A |
| hsa-miR-30b-5p | 4481-4487 | 7mer-m8 | -0.12 | 68 | -0.12 | 4.880 | 0.7 |
| hsa-miR-30c-5p | 4481-4487 | 7mer-m8 | -0.12 | 68 | -0.12 | 4.880 | 0.7 |
| hsa-miR-30a-5p | 4481-4487 | 7mer-m8 | -0.1 | 63 | -0.1 | 4.880 | 0.7 |
| hsa-miR-30d-5p | 4481-4487 | 7mer-m8 | -0.1 | 63 | -0.1 | 4.880 | 0.7 |
| hsa-miR-30e-5p | 4481-4487 | 7mer-m8 | -0.1 | 61 | -0.1 | 4.880 | 0.7 |
| hsa-miR-24-3p | 4566-4572 | 7mer-m8 | -0.32 | 97 | -0.32 | 4.891 | 0.61 |
| hsa-miR-495-3p | 4657-4663 | 7mer-m8 | -0.02 | 78 | -0.02 | 3.318 | N/A |
| hsa-miR-5688 | 4657-4663 | 7mer-m8 | -0.02 | 77 | -0.02 | 3.318 | N/A |
| hsa-miR-363-3p | 4692-4699 | 8mer | -0.34 | 97 | -0.34 | 5.290 | 0.93 |
| hsa-miR-25-3p | 4692-4699 | 8mer | -0.33 | 97 | -0.33 | 5.290 | 0.93 |
| hsa-miR-32-5p | 4692-4699 | 8mer | -0.34 | 97 | -0.34 | 5.290 | 0.93 |
| hsa-miR-367-3p | 4692-4699 | 8mer | -0.34 | 97 | -0.34 | 5.290 | 0.93 |
| hsa-miR-92b-3p | 4692-4699 | 8mer | -0.3 | 96 | -0.3 | 5.290 | 0.93 |
| hsa-miR-92a-3p | 4692-4699 | 8mer | -0.3 | 96 | -0.3 | 5.290 | 0.93 |
| **Poorly conserved sites** | | | | | | | |
| hsa-miR-6766-5p | 15-21 | 7mer-m8 | -0.11 | 54 | -0.11 | 0 | N/A |
| hsa-miR-6756-5p | 15-21 | 7mer-m8 | -0.11 | 52 | -0.11 | 0 | N/A |
| hsa-miR-608 | 16-22 | 7mer-m8 | -0.14 | 52 | -0.14 | 0 | N/A |
| hsa-miR-4651 | 16-22 | 7mer-m8 | -0.11 | 48 | -0.11 | 0 | N/A |
| hsa-miR-6782-5p | 17-23 | 7mer-m8 | -0.09 | 59 | -0.09 | 0 | N/A |
| hsa-miR-1909-3p | 19-26 | 8mer | -0.35 | 97 | -0.35 | 0 | N/A |
| hsa-miR-6722-3p | 19-26 | 8mer | -0.32 | 95 | -0.32 | 0 | N/A |
| hsa-miR-6836-5p | 20-26 | 7mer-1A | -0.04 | 51 | -0.04 | 0.021 | N/A |
| hsa-miR-6132 | 20-26 | 7mer-1A | -0.05 | 50 | -0.05 | 0.021 | N/A |
| hsa-miR-3944-5p | 22-28 | 7mer-1A | -0.02 | 53 | -0.02 | 0 | N/A |
| hsa-miR-143-5p | 22-28 | 7mer-1A | -0.01 | 37 | -0.01 | 0 | N/A |
| hsa-miR-5008-3p | 24-30 | 7mer-1A | -0.14 | 83 | -0.14 | 0 | N/A |
| hsa-miR-6737-3p | 24-30 | 7mer-1A | -0.07 | 68 | -0.07 | 0 | N/A |
| hsa-miR-7157-3p | 24-30 | 7mer-1A | -0.07 | 67 | -0.07 | 0 | N/A |
| hsa-miR-6889-3p | 24-30 | 7mer-1A | -0.01 | 31 | -0.01 | 0.072 | N/A |
| hsa-miR-6780a-3p | 27-33 | 7mer-m8 | -0.04 | 52 | -0.04 | 0 | N/A |
| hsa-miR-4279 | 29-36 | 8mer | -0.33 | 98 | -0.33 | 0 | N/A |
| hsa-miR-4512 | 36-42 | 7mer-m8 | -0.16 | 86 | -0.16 | 0 | N/A |
| hsa-miR-3918 | 37-43 | 7mer-m8 | -0.2 | 88 | -0.2 | 0 | N/A |
| hsa-miR-6132 | 38-45 | 8mer | -0.34 | 96 | -0.34 | 0.075 | N/A |
| hsa-miR-6836-5p | 38-45 | 8mer | -0.34 | 96 | -0.34 | 0.075 | N/A |
| hsa-miR-1909-3p | 39-45 | 7mer-1A | -0.12 | 70 | -0.12 | 0 | N/A |
| hsa-miR-6722-3p | 39-45 | 7mer-1A | -0.08 | 60 | -0.08 | 0 | N/A |
| hsa-miR-5572 | 39-50 | non-canonical | N/A | N/A | N/A | 0 | N/A |
| hsa-miR-5572 | 39-50 | non-canonical | N/A | N/A | N/A | 0 | N/A |
| hsa-miR-342-5p | 43-50 | 8mer | -0.41 | 97 | -0.41 | 0.505 | N/A |
| hsa-miR-4664-5p | 43-50 | 8mer | -0.4 | 97 | -0.4 | 0.505 | N/A |
| hsa-miR-6819-5p | 44-50 | 7mer-m8 | -0.22 | 87 | -0.22 | 0.409 | N/A |
| hsa-miR-6737-5p | 44-50 | 7mer-m8 | -0.2 | 84 | -0.2 | 0.409 | N/A |
| hsa-miR-6812-5p | 44-50 | 7mer-m8 | -0.18 | 78 | -0.18 | 0.409 | N/A |
| hsa-miR-6747-5p | 44-50 | 7mer-1A | -0.11 | 70 | -0.11 | 0.901 | N/A |
| hsa-miR-608 | 44-50 | 7mer-1A | -0.15 | 56 | -0.15 | 0.409 | N/A |
| hsa-miR-4651 | 44-50 | 7mer-1A | -0.14 | 54 | -0.14 | 0.409 | N/A |
| hsa-miR-7155-5p | 45-51 | 7mer-m8 | -0.2 | 93 | -0.2 | 0 | N/A |
| hsa-miR-4731-5p | 46-53 | 8mer | -0.36 | 98 | -0.36 | 0 | N/A |
| hsa-miR-5589-5p | 47-53 | 7mer-1A | -0.05 | 77 | -0.05 | 0 | N/A |
| hsa-miR-3140-5p | 53-60 | 8mer | -0.1 | 81 | -0.1 | 0 | N/A |
| hsa-miR-3653-5p | 55-61 | 7mer-m8 | -0.04 | 68 | -0.04 | 0 | N/A |
| hsa-miR-3670 | 59-65 | 7mer-1A | -0.14 | 71 | -0.14 | 0.072 | N/A |
| hsa-miR-4677-3p | 62-69 | 8mer | -0.11 | 80 | -0.11 | 0 | N/A |
| hsa-miR-4679 | 63-69 | 7mer-1A | -0.01 | 28 | -0.01 | 1.082 | N/A |
| hsa-miR-5691 | 66-72 | 7mer-m8 | -0.11 | 79 | -0.11 | 0 | N/A |
| hsa-miR-6805-3p | 66-72 | 7mer-m8 | -0.07 | 69 | -0.07 | 0 | N/A |
| hsa-miR-3194-3p | 66-72 | 7mer-1A | -0.04 | 58 | -0.04 | 0.901 | N/A |
| hsa-miR-6828-3p | 68-74 | 7mer-m8 | -0.12 | 83 | -0.12 | 0 | N/A |
| hsa-miR-767-3p | 68-74 | 7mer-1A | -0.09 | 71 | -0.09 | 0.072 | N/A |
| hsa-miR-4686 | 69-75 | 7mer-m8 | -0.07 | 66 | -0.07 | 0 | N/A |
| hsa-miR-3165 | 74-80 | 7mer-m8 | -0.13 | 81 | -0.13 | 0.021 | N/A |
| hsa-miR-4722-5p | 83-89 | 7mer-m8 | -0.02 | 34 | -0.02 | 0 | N/A |
| hsa-miR-6165 | 84-90 | 7mer-m8 | -0.02 | 24 | -0.02 | 0 | N/A |
| hsa-miR-3619-5p | 85-91 | 7mer-m8 | -0.06 | 72 | -0.06 | 0.075 | N/A |
| hsa-miR-761 | 85-91 | 7mer-m8 | -0.02 | 55 | -0.02 | 0.075 | N/A |
| hsa-miR-214-3p | 85-91 | 7mer-m8 | -0.02 | 43 | -0.02 | 0.075 | N/A |
| hsa-miR-1184 | 88-94 | 7mer-m8 | -0.02 | 64 | -0.02 | 0.075 | N/A |
| hsa-miR-6829-5p | 91-97 | 7mer-m8 | -0.15 | 83 | -0.15 | 0 | N/A |
| hsa-miR-92b-5p | 96-102 | 7mer-m8 | -0.24 | 69 | -0.24 | 0.072 | N/A |
| hsa-miR-3191-3p | 97-103 | 7mer-m8 | -0.21 | 87 | -0.21 | 0 | N/A |
| hsa-miR-5010-5p | 99-105 | 7mer-1A | -0.16 | 75 | -0.16 | 0 | N/A |
| hsa-miR-625-5p | 99-105 | 7mer-1A | -0.2 | 74 | -0.2 | 0.409 | N/A |
| hsa-miR-4525 | 99-105 | 7mer-1A | -0.1 | 73 | -0.1 | 0 | N/A |
| hsa-miR-1275 | 99-105 | 7mer-m8 | -0.09 | 68 | -0.09 | 0.411 | N/A |
| hsa-miR-4665-5p | 99-105 | 7mer-m8 | -0.1 | 62 | -0.1 | 0.411 | N/A |
| hsa-miR-7111-5p | 99-105 | 7mer-1A | -0.14 | 53 | -0.14 | 0.409 | N/A |
| hsa-miR-6870-5p | 99-105 | 7mer-1A | -0.12 | 52 | -0.12 | 0.409 | N/A |
| hsa-miR-4723-5p | 99-105 | 7mer-1A | -0.12 | 51 | -0.12 | 0.409 | N/A |
| hsa-miR-5698 | 99-105 | 7mer-1A | -0.12 | 51 | -0.12 | 0.409 | N/A |
| hsa-miR-4447 | 100-107 | 8mer | -0.24 | 93 | -0.24 | 0 | N/A |
| hsa-miR-4472 | 100-107 | 8mer | -0.24 | 93 | -0.24 | 0 | N/A |
| hsa-miR-6742-5p | 101-107 | 7mer-1A | -0.13 | 76 | -0.13 | 0.310 | N/A |
| hsa-miR-6796-5p | 101-107 | 7mer-m8 | -0.13 | 70 | -0.13 | 0.409 | N/A |
| hsa-miR-3151-5p | 101-107 | 7mer-1A | -0.02 | 49 | -0.02 | 0.417 | N/A |
| hsa-miR-134-3p | 102-108 | 7mer-m8 | -0.03 | 51 | -0.03 | 0.445 | N/A |
| hsa-miR-505-5p | 110-116 | 7mer-1A | -0.11 | 77 | -0.11 | 0.668 | N/A |
| hsa-miR-4252 | 115-121 | 7mer-m8 | -0.02 | 29 | -0.02 | 0.984 | N/A |
| hsa-miR-449b-3p | 117-123 | 7mer-1A | -0.11 | 83 | -0.11 | 0.409 | N/A |
| hsa-miR-6882-3p | 134-140 | 7mer-1A | -0.1 | 66 | -0.1 | 0 | N/A |
| hsa-miR-3649 | 144-150 | 7mer-1A | -0.2 | 84 | -0.2 | 0 | N/A |
| hsa-miR-4648 | 145-151 | 7mer-m8 | -0.13 | 83 | -0.13 | 0 | N/A |
| hsa-miR-3677-3p | 147-153 | 7mer-m8 | -0.21 | 64 | -0.21 | 0 | N/A |
| hsa-miR-3907 | 152-158 | 7mer-m8 | -0.22 | 87 | -0.22 | 0 | N/A |
| hsa-miR-4735-3p | 154-161 | 8mer | -0.33 | 97 | -0.33 | 0.06 | N/A |
| hsa-miR-18a-5p | 154-161 | 8mer | -0.33 | 97 | -0.33 | 0.06 | N/A |
| hsa-miR-18b-5p | 154-161 | 8mer | -0.33 | 97 | -0.33 | 0.06 | N/A |
| hsa-miR-4284 | 162-168 | 7mer-1A | -0.01 | 34 | -0.01 | 0 | N/A |
| hsa-miR-3064-5p | 164-170 | 7mer-1A | -0.17 | 88 | -0.17 | 3.404 | N/A |
| hsa-miR-664a-5p | 164-170 | 7mer-1A | -0.15 | 87 | -0.15 | 0 | N/A |
| hsa-miR-4794 | 164-170 | 7mer-1A | -0.1 | 81 | -0.1 | 0 | N/A |
| hsa-miR-6504-5p | 164-170 | 7mer-1A | -0.12 | 79 | -0.12 | 3.404 | N/A |
| hsa-miR-765 | 172-179 | 8mer | -0.16 | 77 | -0.16 | 0.075 | N/A |
| hsa-miR-766-5p | 173-179 | 7mer-1A | -0.11 | 71 | -0.11 | 0 | N/A |
| hsa-miR-6894-5p | 173-179 | 7mer-1A | -0.08 | 69 | -0.08 | 0 | N/A |
| hsa-miR-7154-3p | 173-179 | 7mer-1A | -0.02 | 47 | -0.02 | 0 | N/A |
| hsa-miR-3135b | 175-181 | 7mer-m8 | -0.08 | 72 | -0.08 | 0 | N/A |
| hsa-miR-626 | 182-188 | 7mer-1A | -0.1 | 60 | -0.1 | 0.021 | N/A |
| hsa-miR-6876-3p | 182-188 | 7mer-1A | -0.07 | 52 | -0.07 | 0.021 | N/A |
| hsa-miR-6891-3p | 195-201 | 7mer-1A | -0.01 | 33 | -0.01 | 0 | N/A |
| hsa-miR-6072 | 195-201 | 7mer-1A | -0.01 | 30 | -0.01 | 0 | N/A |
| hsa-miR-4279 | 211-217 | 7mer-m8 | -0.1 | 83 | -0.1 | 0 | N/A |
| hsa-miR-6845-3p | 212-218 | 7mer-m8 | -0.05 | 58 | -0.05 | 0 | N/A |
| hsa-miR-6741-3p | 215-222 | 8mer | -0.44 | 98 | -0.44 | 0 | N/A |
| hsa-miR-513c-3p | 221-227 | 7mer-m8 | -0.02 | 78 | -0.02 | 0 | N/A |
| hsa-miR-3606-3p | 221-227 | 7mer-m8 | -0.02 | 78 | -0.02 | 0 | N/A |
| hsa-miR-513a-3p | 221-227 | 7mer-m8 | -0.02 | 78 | -0.02 | 0 | N/A |
| hsa-miR-520d-5p | 225-231 | 7mer-1A | -0.02 | 77 | -0.02 | 0 | N/A |
| hsa-miR-524-5p | 225-231 | 7mer-1A | -0.02 | 75 | -0.02 | 0 | N/A |
| hsa-miR-2681-5p | 228-234 | 7mer-1A | -0.01 | 53 | -0.01 | 0 | N/A |
| hsa-miR-153-5p | 230-236 | 7mer-m8 | -0.02 | 50 | -0.02 | 0.072 | N/A |
| hsa-miR-6512-5p | 233-239 | 7mer-1A | -0.09 | 76 | -0.09 | 0 | N/A |
| hsa-miR-6848-3p | 243-249 | 7mer-1A | -0.09 | 86 | -0.09 | 0 | N/A |
| hsa-miR-6843-3p | 243-249 | 7mer-1A | -0.16 | 85 | -0.16 | 0 | N/A |
| hsa-miR-133a-3p.2 | 244-250 | 7mer-1A | -0.14 | 76 | -0.14 | 1.628 | N/A |
| hsa-miR-133b | 244-250 | 7mer-1A | -0.14 | 76 | -0.14 | 1.628 | N/A |
| hsa-miR-138-2-3p | 250-256 | 7mer-1A | -0.01 | 48 | -0.01 | 0 | N/A |
| hsa-miR-4482-3p | 251-257 | 7mer-m8 | -0.06 | 83 | -0.06 | 0 | N/A |
| hsa-miR-6505-3p | 254-261 | 8mer | -0.43 | 98 | -0.43 | 0 | N/A |
| hsa-miR-617 | 255-261 | 7mer-1A | -0.16 | 82 | -0.16 | 0.021 | N/A |
| hsa-miR-580-3p | 264-270 | 7mer-m8 | -0.14 | 93 | -0.14 | 0.021 | N/A |
| hsa-miR-7154-5p | 268-274 | 7mer-1A | -0.1 | 77 | -0.1 | 0 | N/A |
| hsa-miR-433-3p | 268-274 | 7mer-1A | -0.06 | 73 | -0.06 | 0.695 | N/A |
| hsa-miR-10b-3p | 272-278 | 7mer-m8 | -0.14 | 89 | -0.14 | 0 | N/A |
| hsa-miR-488-5p | 274-280 | 7mer-1A | -0.15 | 84 | -0.15 | 0.467 | N/A |
| hsa-miR-3925-3p | 276-282 | 7mer-1A | -0.12 | 86 | -0.12 | 0 | N/A |
| hsa-miR-766-3p | 276-282 | 7mer-1A | -0.07 | 73 | -0.07 | 0.021 | N/A |
| hsa-miR-519e-5p | 277-283 | 7mer-1A | -0.1 | 80 | -0.1 | 0.072 | N/A |
| hsa-miR-515-5p | 277-283 | 7mer-1A | -0.08 | 72 | -0.08 | 0.072 | N/A |
| hsa-miR-4273 | 279-286 | 8mer | -0.15 | 95 | -0.15 | 0 | N/A |
| hsa-miR-7156-5p | 279-286 | 8mer | -0.21 | 95 | -0.21 | 0 | N/A |
| hsa-miR-2117 | 279-285 | 7mer-1A | -0.09 | 63 | -0.09 | 0 | N/A |
| hsa-miR-6739-3p | 280-287 | 8mer | -0.18 | 89 | -0.18 | 0 | N/A |
| hsa-miR-4677-5p | 280-286 | 7mer-1A | -0.06 | 75 | -0.06 | 0.072 | N/A |
| hsa-miR-375 | 281-287 | 7mer-1A | -0.08 | 81 | -0.08 | 2.207 | N/A |
| hsa-miR-1290 | 292-298 | 7mer-m8 | -0.11 | 81 | -0.11 | 0.021 | N/A |
| hsa-miR-191-5p | 296-302 | 7mer-1A | -0.27 | 81 | -0.27 | 0.105 | N/A |
| hsa-miR-3607-3p | 300-306 | 7mer-1A | -0.15 | 92 | -0.15 | 0 | N/A |
| hsa-miR-3686 | 300-307 | 8mer | -0.12 | 77 | -0.12 | 0 | N/A |
| hsa-miR-4743-3p | 302-308 | 7mer-1A | -0.01 | 39 | -0.01 | 0 | N/A |
| hsa-miR-4652-3p | 302-308 | 7mer-1A | -0.01 | 36 | -0.01 | 0 | N/A |
| hsa-miR-33b-5p | 307-313 | 7mer-1A | -0.07 | 83 | -0.07 | 1.681 | N/A |
| hsa-miR-33a-5p | 307-313 | 7mer-1A | -0.07 | 83 | -0.07 | 1.681 | N/A |
| hsa-miR-3680-3p | 308-314 | 7mer-m8 | -0.11 | 89 | -0.11 | 0 | N/A |
| hsa-miR-4450 | 317-323 | 7mer-m8 | -0.2 | 84 | -0.2 | 0 | N/A |
| hsa-miR-5010-5p | 318-325 | 8mer | -0.46 | 98 | -0.46 | 0 | N/A |
| hsa-miR-4525 | 318-325 | 8mer | -0.39 | 98 | -0.39 | 0 | N/A |
| hsa-miR-625-5p | 319-325 | 7mer-1A | -0.29 | 90 | -0.29 | 0 | N/A |
| hsa-miR-1275 | 319-325 | 7mer-m8 | -0.17 | 86 | -0.17 | 0.021 | N/A |
| hsa-miR-4665-5p | 319-325 | 7mer-m8 | -0.22 | 84 | -0.22 | 0.021 | N/A |
| hsa-miR-5698 | 319-325 | 7mer-1A | -0.23 | 75 | -0.23 | 0 | N/A |
| hsa-miR-7111-5p | 319-325 | 7mer-1A | -0.22 | 72 | -0.22 | 0 | N/A |
| hsa-miR-6870-5p | 319-325 | 7mer-1A | -0.2 | 70 | -0.2 | 0 | N/A |
| hsa-miR-4723-5p | 319-325 | 7mer-1A | -0.2 | 69 | -0.2 | 0 | N/A |
| hsa-miR-637 | 320-326 | 7mer-m8 | -0.27 | 94 | -0.27 | 0.021 | N/A |
| hsa-miR-4731-5p | 321-327 | 7mer-m8 | -0.26 | 95 | -0.26 | 0 | N/A |
| hsa-miR-1207-3p | 323-330 | 8mer | -0.27 | 97 | -0.27 | 0.021 | N/A |
| hsa-miR-6787-3p | 325-332 | 8mer | -0.17 | 93 | -0.17 | 0 | N/A |
| hsa-miR-942-5p | 329-335 | 7mer-1A | -0.07 | 72 | -0.07 | 0.021 | N/A |
| hsa-miR-4659b-3p | 330-337 | 8mer | -0.03 | 56 | -0.03 | 0 | N/A |
| hsa-miR-4659a-3p | 330-337 | 8mer | -0.03 | 56 | -0.03 | 0 | N/A |
| hsa-miR-6875-3p | 331-337 | 7mer-1A | -0.03 | 66 | -0.03 | 0 | N/A |
| hsa-miR-3160-5p | 333-339 | 7mer-m8 | -0.04 | 67 | -0.04 | 0 | N/A |
| hsa-miR-7845-5p | 340-346 | 7mer-m8 | -0.02 | 13 | -0.02 | 0 | N/A |
| hsa-miR-211-3p | 341-347 | 7mer-m8 | -0.02 | 63 | -0.02 | 0 | N/A |
| hsa-miR-4763-3p | 343-350 | 8mer | -0.26 | 93 | -0.26 | 0 | N/A |
| hsa-miR-1207-5p | 343-350 | 8mer | -0.24 | 91 | -0.24 | 0 | N/A |
| hsa-miR-7150 | 344-350 | 7mer-m8 | -0.05 | 55 | -0.05 | 0 | N/A |
| hsa-miR-4736 | 344-350 | 7mer-1A | -0.03 | 48 | -0.03 | 0 | N/A |
| hsa-miR-5581-3p | 349-355 | 7mer-1A | -0.01 | 34 | -0.01 | 0 | N/A |
| hsa-miR-587 | 350-356 | 7mer-1A | -0.01 | 25 | -0.01 | 0.021 | N/A |
| hsa-miR-361-3p | 365-371 | 7mer-m8 | -0.05 | 53 | -0.05 | 0.075 | N/A |
| hsa-miR-6859-3p | 367-374 | 8mer | -0.29 | 92 | -0.29 | 0 | N/A |
| hsa-miR-6846-3p | 368-374 | 7mer-1A | -0.08 | 60 | -0.08 | 0 | N/A |
| hsa-miR-4750-3p | 369-375 | 7mer-m8 | -0.03 | 60 | -0.03 | 0 | N/A |
| hsa-miR-4271 | 376-382 | 7mer-m8 | -0.02 | 35 | -0.02 | 0 | N/A |
| hsa-miR-4725-3p | 376-382 | 7mer-m8 | -0.02 | 24 | -0.02 | 0 | N/A |
| hsa-miR-6780b-5p | 376-382 | 7mer-m8 | -0.02 | 24 | -0.02 | 0 | N/A |
| hsa-miR-542-5p | 378-384 | 7mer-1A | -0.18 | 79 | -0.18 | 0.072 | N/A |
| hsa-miR-6777-5p | 378-384 | 7mer-1A | -0.18 | 58 | -0.18 | 0 | N/A |
| hsa-miR-6889-5p | 378-384 | 7mer-1A | -0.14 | 52 | -0.14 | 0 | N/A |
| hsa-miR-483-3p.1 | 383-389 | 7mer-m8 | -0.04 | 67 | -0.04 | 0.418 | N/A |
| hsa-miR-1972 | 390-396 | 7mer-m8 | -0.02 | 21 | -0.02 | 0.072 | N/A |
| hsa-miR-6511b-5p | 392-399 | 8mer | -0.2 | 89 | -0.2 | 0 | N/A |
| hsa-miR-6811-5p | 392-399 | 8mer | -0.17 | 87 | -0.17 | 0 | N/A |
| hsa-miR-182-3p | 399-405 | 7mer-1A | -0.15 | 81 | -0.15 | 0 | N/A |
| hsa-miR-152-5p | 399-405 | 7mer-1A | -0.09 | 61 | -0.09 | 0 | N/A |
| hsa-miR-597-3p | 399-405 | 7mer-1A | -0.03 | 52 | -0.03 | 0.409 | N/A |
| hsa-miR-5001-5p | 403-410 | 8mer | -0.47 | 98 | -0.47 | 0.075 | N/A |
| hsa-miR-4492 | 403-410 | 8mer | -0.39 | 98 | -0.39 | 0.075 | N/A |
| hsa-miR-4498 | 403-410 | 8mer | -0.47 | 98 | -0.47 | 0.075 | N/A |
| hsa-miR-762 | 403-410 | 8mer | -0.41 | 98 | -0.41 | 0.075 | N/A |
| hsa-miR-4741 | 404-410 | 7mer-1A | -0.14 | 85 | -0.14 | 0 | N/A |
| hsa-miR-4675 | 404-410 | 7mer-1A | -0.08 | 82 | -0.08 | 0 | N/A |
| hsa-miR-4656 | 404-410 | 7mer-1A | -0.15 | 80 | -0.15 | 0 | N/A |
| hsa-miR-1587 | 404-410 | 7mer-m8 | -0.15 | 79 | -0.15 | 0 | N/A |
| hsa-miR-3620-5p | 404-410 | 7mer-m8 | -0.1 | 78 | -0.1 | 0 | N/A |
| hsa-miR-6829-5p | 404-410 | 7mer-1A | -0.09 | 74 | -0.09 | 0 | N/A |
| hsa-miR-4417 | 405-411 | 7mer-m8 | -0.1 | 74 | -0.1 | 0 | N/A |
| hsa-miR-541-3p | 406-412 | 7mer-m8 | -0.26 | 92 | -0.26 | 0.072 | N/A |
| hsa-miR-654-5p | 406-412 | 7mer-m8 | -0.24 | 91 | -0.24 | 0.072 | N/A |
| hsa-miR-3665 | 409-415 | 7mer-m8 | -0.12 | 66 | -0.12 | 0 | N/A |
| hsa-miR-6081 | 412-418 | 7mer-m8 | -0.2 | 82 | -0.2 | 0 | N/A |
| hsa-miR-133a-5p | 419-426 | 8mer | -0.13 | 75 | -0.13 | 0.452 | N/A |
| hsa-miR-6131 | 420-426 | 7mer-1A | -0.01 | 30 | -0.01 | 0.021 | N/A |
| hsa-miR-3692-5p | 421-428 | 8mer | -0.26 | 96 | -0.26 | 0.415 | N/A |
| hsa-miR-93-3p | 422-428 | 7mer-1A | -0.09 | 74 | -0.09 | 0.705 | N/A |
| hsa-miR-6071 | 422-428 | 7mer-m8 | -0.05 | 56 | -0.05 | 0.409 | N/A |
| hsa-miR-4686 | 423-429 | 7mer-m8 | -0.02 | 30 | -0.02 | 0 | N/A |
| hsa-miR-646 | 431-437 | 7mer-m8 | -0.13 | 85 | -0.13 | 0.021 | N/A |
| hsa-miR-4502 | 447-453 | 7mer-m8 | -0.02 | 40 | -0.02 | 0.609 | N/A |
| hsa-miR-3691-3p | 456-462 | 7mer-1A | -0.02 | 57 | -0.02 | 0 | N/A |
| hsa-miR-5695 | 457-464 | 8mer | -0.18 | 92 | -0.18 | 0 | N/A |
| hsa-miR-519d-5p | 458-464 | 7mer-1A | -0.05 | 62 | -0.05 | 0 | N/A |
| hsa-miR-515-5p | 458-464 | 7mer-m8 | -0.02 | 31 | -0.02 | 0.072 | N/A |
| hsa-miR-519e-5p | 458-464 | 7mer-m8 | -0.02 | 31 | -0.02 | 0.072 | N/A |
| hsa-miR-4660 | 463-470 | 8mer | -0.23 | 91 | -0.23 | 0.021 | N/A |
| hsa-miR-5047 | 464-471 | 8mer | -0.2 | 91 | -0.2 | 0 | N/A |
| hsa-miR-1301-3p | 464-471 | 8mer | -0.19 | 90 | -0.19 | 0 | N/A |
| hsa-miR-7156-3p | 465-471 | 7mer-1A | -0.06 | 62 | -0.06 | 0 | N/A |
| hsa-miR-4317 | 468-474 | 7mer-1A | -0.01 | 34 | -0.01 | 0 | N/A |
| hsa-miR-885-3p | 476-482 | 7mer-1A | -0.14 | 83 | -0.14 | 0.075 | N/A |
| hsa-miR-6823-5p | 482-488 | 7mer-1A | -0.03 | 50 | -0.03 | 0.310 | N/A |
| hsa-miR-3127-5p | 483-489 | 7mer-1A | -0.07 | 60 | -0.07 | 0.021 | N/A |
| hsa-miR-6515-3p | 486-492 | 7mer-m8 | -0.02 | 32 | -0.02 | 0 | N/A |
| hsa-miR-877-3p | 487-494 | 8mer | -0.16 | 85 | -0.16 | 0 | N/A |
| hsa-miR-6881-3p | 488-495 | 8mer | -0.05 | 60 | -0.05 | 0 | N/A |
| hsa-miR-6780a-3p | 489-495 | 7mer-1A | -0.03 | 49 | -0.03 | 0.625 | N/A |
| hsa-miR-7111-3p | 489-495 | 7mer-1A | -0.01 | 33 | -0.01 | 0 | N/A |
| hsa-miR-1238-3p | 490-496 | 7mer-1A | -0.09 | 80 | -0.09 | 0 | N/A |
| hsa-miR-670-3p | 490-496 | 7mer-1A | -0.11 | 78 | -0.11 | 0.108 | N/A |
| hsa-miR-6814-3p | 496-502 | 7mer-m8 | -0.31 | 90 | -0.31 | 0 | N/A |
| hsa-miR-6872-5p | 496-502 | 7mer-m8 | -0.29 | 89 | -0.29 | 0 | N/A |
| hsa-miR-4471 | 501-508 | 8mer | -0.17 | 82 | -0.17 | 0 | N/A |
| hsa-miR-8059 | 501-508 | 8mer | -0.12 | 82 | -0.12 | 0 | N/A |
| hsa-miR-1292-5p | 502-508 | 7mer-1A | -0.13 | 82 | -0.13 | 0.507 | N/A |
| hsa-miR-4646-5p | 503-509 | 7mer-m8 | -0.03 | 52 | -0.03 | 0.468 | N/A |
| hsa-miR-204-3p | 503-509 | 7mer-m8 | -0.02 | 50 | -0.02 | 0.468 | N/A |
| hsa-miR-4731-5p | 505-511 | 7mer-1A | -0.15 | 83 | -0.15 | 0 | N/A |
| hsa-miR-5589-5p | 505-511 | 7mer-1A | -0.06 | 81 | -0.06 | 0 | N/A |
| hsa-miR-1282 | 509-515 | 7mer-m8 | -0.28 | 87 | -0.28 | 0.021 | N/A |
| hsa-miR-92b-5p | 514-520 | 7mer-1A | -0.24 | 69 | -0.24 | 0.072 | N/A |
| hsa-miR-659-5p | 526-533 | 8mer | -0.43 | 98 | -0.43 | 0 | N/A |
| hsa-miR-4535 | 527-533 | 7mer-m8 | -0.2 | 92 | -0.2 | 0 | N/A |
| hsa-miR-492 | 527-533 | 7mer-1A | -0.16 | 72 | -0.16 | 0.075 | N/A |
| hsa-miR-6861-3p | 527-533 | 7mer-1A | -0.07 | 62 | -0.07 | 0 | N/A |
| hsa-miR-3654 | 529-535 | 7mer-m8 | -0.12 | 89 | -0.12 | 0 | N/A |
| hsa-miR-653-3p | 531-537 | 7mer-m8 | -0.16 | 91 | -0.16 | 0.072 | N/A |
| hsa-miR-34c-3p | 533-539 | 7mer-1A | -0.09 | 80 | -0.09 | 0.075 | N/A |
| hsa-miR-572 | 540-546 | 7mer-1A | -0.18 | 74 | -0.18 | 0.075 | N/A |
| hsa-miR-4297 | 543-549 | 7mer-m8 | -0.08 | 60 | -0.08 | 0.409 | N/A |
| hsa-miR-5581-5p | 543-549 | 7mer-m8 | -0.08 | 56 | -0.08 | 0.409 | N/A |
| hsa-miR-7108-5p | 547-553 | 7mer-m8 | -0.09 | 79 | -0.09 | 0 | N/A |
| hsa-miR-663b | 547-553 | 7mer-1A | -0.04 | 59 | -0.04 | 0.021 | N/A |
| hsa-miR-4318 | 549-555 | 7mer-m8 | -0.04 | 58 | -0.04 | 0 | N/A |
| hsa-miR-943 | 551-557 | 7mer-m8 | -0.02 | 38 | -0.02 | 0.021 | N/A |
| hsa-miR-4656 | 555-562 | 8mer | -0.12 | 75 | -0.12 | 0 | N/A |
| hsa-miR-4492 | 556-562 | 7mer-1A | -0.16 | 86 | -0.16 | 0.075 | N/A |
| hsa-miR-4675 | 556-562 | 7mer-1A | -0.1 | 86 | -0.1 | 0 | N/A |
| hsa-miR-4741 | 556-562 | 7mer-1A | -0.15 | 86 | -0.15 | 0 | N/A |
| hsa-miR-762 | 556-562 | 7mer-1A | -0.17 | 85 | -0.17 | 0.075 | N/A |
| hsa-miR-5001-5p | 556-562 | 7mer-1A | -0.2 | 82 | -0.2 | 0.075 | N/A |
| hsa-miR-4498 | 556-562 | 7mer-1A | -0.19 | 79 | -0.19 | 0.075 | N/A |
| hsa-miR-1587 | 556-562 | 7mer-m8 | -0.12 | 73 | -0.12 | 0 | N/A |
| hsa-miR-3620-5p | 556-562 | 7mer-m8 | -0.07 | 71 | -0.07 | 0 | N/A |
| hsa-miR-6829-5p | 556-562 | 7mer-1A | -0.07 | 67 | -0.07 | 0 | N/A |
| hsa-miR-378g | 557-563 | 7mer-m8 | -0.05 | 64 | -0.05 | 0 | N/A |
| hsa-miR-939-3p | 558-564 | 7mer-m8 | -0.11 | 77 | -0.11 | 0 | N/A |
| hsa-miR-4292 | 559-565 | 7mer-m8 | -0.1 | 67 | -0.1 | 0 | N/A |
| hsa-miR-6791-5p | 559-565 | 7mer-m8 | -0.08 | 60 | -0.08 | 0 | N/A |
| hsa-miR-6775-3p | 561-568 | 8mer | -0.21 | 83 | -0.21 | 0.021 | N/A |
| hsa-miR-1291 | 561-568 | 8mer | -0.19 | 80 | -0.19 | 0.021 | N/A |
| hsa-miR-328-3p | 562-568 | 7mer-m8 | -0.05 | 49 | -0.05 | 0.098 | N/A |
| hsa-miR-6851-3p | 562-568 | 7mer-1A | -0.04 | 47 | -0.04 | 0 | N/A |
| hsa-miR-663b | 563-570 | 8mer | -0.18 | 88 | -0.18 | 0.021 | N/A |
| hsa-miR-7108-5p | 564-570 | 7mer-m8 | -0.03 | 62 | -0.03 | 0 | N/A |
| hsa-miR-644a | 565-571 | 7mer-m8 | -0.05 | 56 | -0.05 | 0 | N/A |
| hsa-miR-4738-5p | 571-577 | 7mer-1A | -0.06 | 41 | -0.06 | 0 | N/A |
| hsa-miR-766-3p | 572-579 | 8mer | -0.03 | 56 | -0.03 | 0.021 | N/A |
| hsa-miR-3925-3p | 573-579 | 7mer-1A | -0.01 | 41 | -0.01 | 0 | N/A |
| hsa-miR-2682-3p | 577-583 | 7mer-1A | -0.05 | 59 | -0.05 | 0.409 | N/A |
| hsa-miR-6781-3p | 577-583 | 7mer-1A | -0.03 | 46 | -0.03 | 0.409 | N/A |
| hsa-miR-5001-3p | 579-585 | 7mer-m8 | -0.02 | 27 | -0.02 | 0 | N/A |
| hsa-miR-3194-3p | 581-587 | 7mer-m8 | -0.02 | 48 | -0.02 | 0 | N/A |
| hsa-miR-760 | 582-588 | 7mer-m8 | -0.02 | 24 | -0.02 | 0.117 | N/A |
| hsa-miR-6741-3p | 583-589 | 7mer-m8 | -0.1 | 49 | -0.1 | 0 | N/A |
| hsa-miR-3141 | 587-593 | 7mer-m8 | -0.04 | 25 | -0.04 | 0 | N/A |
| hsa-miR-6515-5p | 590-596 | 7mer-m8 | -0.02 | 32 | -0.02 | 0 | N/A |
| hsa-miR-6797-5p | 590-596 | 7mer-1A | -0.03 | 21 | -0.03 | 0.445 | N/A |
| hsa-miR-1249-5p | 590-596 | 7mer-1A | -0.01 | 9 | -0.01 | 0.445 | N/A |
| hsa-miR-4254 | 592-598 | 7mer-m8 | -0.02 | 42 | -0.02 | 0 | N/A |
| hsa-miR-4308 | 593-599 | 7mer-m8 | -0.02 | 30 | -0.02 | 0 | N/A |
| hsa-miR-504-5p.1 | 594-600 | 7mer-m8 | -0.02 | 44 | -0.02 | 0.441 | N/A |
| hsa-miR-3620-3p | 595-602 | 8mer | -0.05 | 58 | -0.05 | 0 | N/A |
| hsa-miR-6865-3p | 596-602 | 7mer-1A | -0.01 | 33 | -0.01 | 0 | N/A |
| hsa-miR-6802-3p | 597-603 | 7mer-1A | -0.01 | 27 | -0.01 | 0 | N/A |
| hsa-miR-4687-3p | 602-608 | 7mer-m8 | -0.02 | 32 | -0.02 | 0 | N/A |
| hsa-miR-764 | 625-632 | 8mer | -0.07 | 87 | -0.07 | 0 | N/A |
| hsa-miR-125a-3p | 626-632 | 7mer-1A | -0.07 | 70 | -0.07 | 0.448 | N/A |
| hsa-miR-3934-5p | 626-632 | 7mer-1A | -0.01 | 34 | -0.01 | 0 | N/A |
| hsa-miR-381-5p | 632-638 | 7mer-m8 | -0.15 | 70 | -0.15 | 0.072 | N/A |
| hsa-miR-1202 | 637-643 | 7mer-m8 | -0.02 | 57 | -0.02 | 0.021 | N/A |
| hsa-miR-3972 | 637-643 | 7mer-m8 | -0.03 | 54 | -0.03 | 0.021 | N/A |
| hsa-miR-3194-5p | 637-643 | 7mer-1A | -0.01 | 25 | -0.01 | 0 | N/A |
| hsa-miR-449a | 642-648 | 7mer-m8 | -0.02 | 19 | -0.02 | 0.992 | N/A |
| hsa-miR-34a-5p | 642-648 | 7mer-m8 | -0.02 | 18 | -0.02 | 0.992 | N/A |
| hsa-miR-34c-5p | 642-648 | 7mer-m8 | -0.02 | 17 | -0.02 | 0.992 | N/A |
| hsa-miR-449b-5p | 642-648 | 7mer-m8 | -0.02 | 17 | -0.02 | 0.992 | N/A |
| hsa-miR-1915-5p | 650-656 | 7mer-m8 | -0.04 | 48 | -0.04 | 0 | N/A |
| hsa-miR-4312 | 652-658 | 7mer-1A | -0.01 | 36 | -0.01 | 0 | N/A |
| hsa-miR-5001-3p | 654-660 | 7mer-m8 | -0.02 | 27 | -0.02 | 0 | N/A |
| hsa-miR-6728-3p | 656-662 | 7mer-1A | -0.01 | 32 | -0.01 | 0 | N/A |
| hsa-miR-6817-3p | 657-663 | 7mer-m8 | -0.02 | 38 | -0.02 | 0 | N/A |
| hsa-miR-4639-3p | 659-665 | 7mer-m8 | -0.06 | 75 | -0.06 | 0.445 | N/A |
| hsa-miR-5708 | 671-677 | 7mer-m8 | -0.1 | 35 | -0.1 | 0 | N/A |
| hsa-miR-4792 | 673-680 | 8mer | -0.18 | 86 | -0.18 | 0 | N/A |
| hsa-miR-7855-5p | 674-680 | 7mer-m8 | -0.02 | 35 | -0.02 | 0 | N/A |
| hsa-miR-4710 | 674-680 | 7mer-1A | -0.01 | 28 | -0.01 | 0 | N/A |
| hsa-miR-4299 | 675-681 | 7mer-m8 | -0.02 | 36 | -0.02 | 0 | N/A |
| hsa-miR-4430 | 678-684 | 7mer-1A | -0.23 | 95 | -0.23 | 0.409 | N/A |
| hsa-miR-4505 | 678-684 | 7mer-1A | -0.26 | 93 | -0.26 | 0.409 | N/A |
| hsa-miR-3652 | 678-684 | 7mer-1A | -0.18 | 91 | -0.18 | 0.409 | N/A |
| hsa-miR-5787 | 678-684 | 7mer-1A | -0.16 | 87 | -0.16 | 0.409 | N/A |
| hsa-miR-6842-3p | 678-684 | 7mer-m8 | -0.1 | 69 | -0.1 | 0.468 | N/A |
| hsa-miR-892b | 680-686 | 7mer-m8 | -0.02 | 20 | -0.02 | 0.072 | N/A |
| hsa-miR-4793-3p | 684-690 | 7mer-m8 | -0.02 | 36 | -0.02 | 0.468 | N/A |
| hsa-miR-3137 | 690-696 | 7mer-m8 | -0.05 | 70 | -0.05 | 0 | N/A |
| hsa-miR-1537-5p | 692-698 | 7mer-1A | -0.05 | 23 | -0.05 | 0 | N/A |
| hsa-miR-4718 | 692-698 | 7mer-1A | -0.01 | 17 | -0.01 | 0 | N/A |
| hsa-miR-4999-5p | 692-698 | 7mer-1A | -0.01 | 10 | -0.01 | 0 | N/A |
| hsa-miR-3907 | 698-704 | 7mer-1A | -0.06 | 56 | -0.06 | 0.310 | N/A |
| hsa-miR-4802-3p | 708-714 | 7mer-m8 | -0.02 | 22 | -0.02 | 0 | N/A |
| hsa-miR-4717-3p | 709-715 | 7mer-m8 | -0.05 | 66 | -0.05 | 0 | N/A |
| hsa-miR-188-3p | 712-718 | 7mer-m8 | -0.06 | 56 | -0.06 | 0 | N/A |
| hsa-miR-532-3p | 713-720 | 8mer | -0.2 | 94 | -0.2 | 0.201 | N/A |
| hsa-miR-2116-3p | 714-720 | 7mer-1A | -0.04 | 74 | -0.04 | 0 | N/A |
| hsa-miR-150-5p | 714-720 | 7mer-1A | -0.03 | 65 | -0.03 | 0.201 | N/A |
| hsa-miR-4713-5p | 714-720 | 7mer-m8 | -0.03 | 31 | -0.03 | 0 | N/A |
| hsa-miR-6818-3p | 717-723 | 7mer-1A | -0.01 | 28 | -0.01 | 0 | N/A |
| hsa-miR-3655 | 719-725 | 7mer-1A | -0.05 | 75 | -0.05 | 0 | N/A |
| hsa-miR-578 | 720-726 | 7mer-m8 | -0.02 | 50 | -0.02 | 0.072 | N/A |
| hsa-miR-103a-2-5p | 722-728 | 7mer-m8 | -0.02 | 33 | -0.02 | 0.072 | N/A |
| hsa-miR-6815-3p | 723-729 | 7mer-m8 | -0.11 | 73 | -0.11 | 0 | N/A |
| hsa-miR-604 | 729-735 | 7mer-m8 | -0.12 | 77 | -0.12 | 0.021 | N/A |
| hsa-miR-4764-3p | 736-742 | 7mer-m8 | -0.02 | 43 | -0.02 | 0 | N/A |
| hsa-miR-520f-5p | 740-746 | 7mer-1A | -0.08 | 73 | -0.08 | 0 | N/A |
| hsa-miR-3675-3p | 741-747 | 7mer-m8 | -0.03 | 51 | -0.03 | 0 | N/A |
| hsa-miR-141-5p | 751-757 | 7mer-m8 | -0.06 | 69 | -0.06 | 0 | N/A |
| hsa-miR-107 | 756-762 | 7mer-m8 | -0.02 | 28 | -0.02 | 0.809 | N/A |
| hsa-miR-103a-3p | 756-762 | 7mer-m8 | -0.02 | 28 | -0.02 | 0.809 | N/A |
| hsa-miR-7155-3p | 766-772 | 7mer-1A | -0.11 | 73 | -0.11 | 0 | N/A |
| hsa-miR-3136-3p | 766-772 | 7mer-1A | -0.08 | 65 | -0.08 | 0 | N/A |
| hsa-miR-3176 | 768-774 | 7mer-1A | -0.05 | 64 | -0.05 | 0 | N/A |
| hsa-miR-3190-5p | 768-774 | 7mer-1A | -0.07 | 63 | -0.07 | 0 | N/A |
| hsa-miR-3922-3p | 768-774 | 7mer-1A | -0.03 | 48 | -0.03 | 0 | N/A |
| hsa-miR-4320 | 772-778 | 7mer-m8 | -0.02 | 35 | -0.02 | 0 | N/A |
| hsa-miR-7113-5p | 776-783 | 8mer | -0.11 | 76 | -0.11 | 0.409 | N/A |
| hsa-miR-6837-5p | 777-783 | 7mer-1A | -0.02 | 42 | -0.02 | 0 | N/A |
| hsa-miR-4685-5p | 777-783 | 7mer-1A | -0.02 | 40 | -0.02 | 0 | N/A |
| hsa-miR-455-3p.1 | 780-786 | 7mer-m8 | -0.02 | 33 | -0.02 | 1.591 | N/A |
| hsa-miR-2682-5p | 783-789 | 7mer-m8 | -0.05 | 73 | -0.05 | 0 | N/A |
| hsa-miR-34b-5p | 783-789 | 7mer-m8 | -0.04 | 67 | -0.04 | 0 | N/A |
| hsa-miR-449c-5p | 783-789 | 7mer-m8 | -0.03 | 56 | -0.03 | 0 | N/A |
| hsa-miR-4514 | 784-790 | 7mer-m8 | -0.03 | 53 | -0.03 | 0 | N/A |
| hsa-miR-4692 | 784-790 | 7mer-m8 | -0.02 | 40 | -0.02 | 0 | N/A |
| hsa-miR-4645-5p | 785-791 | 7mer-m8 | -0.02 | 32 | -0.02 | 0.072 | N/A |
| hsa-miR-4673 | 785-791 | 7mer-m8 | -0.02 | 28 | -0.02 | 0.072 | N/A |
| hsa-miR-4755-3p | 786-792 | 7mer-m8 | -0.1 | 67 | -0.1 | 0 | N/A |
| hsa-miR-4258 | 789-795 | 7mer-m8 | -0.02 | 5 | -0.02 | 0 | N/A |
| hsa-miR-4707-3p | 790-796 | 7mer-m8 | -0.19 | 52 | -0.19 | 0 | N/A |
| hsa-miR-7160-3p | 793-799 | 7mer-m8 | -0.02 | 36 | -0.02 | 0 | N/A |
| hsa-miR-3918 | 794-800 | 7mer-m8 | -0.02 | 39 | -0.02 | 0 | N/A |
| hsa-miR-6836-5p | 795-801 | 7mer-m8 | -0.02 | 37 | -0.02 | 0.075 | N/A |
| hsa-miR-6132 | 795-801 | 7mer-m8 | -0.02 | 32 | -0.02 | 0.075 | N/A |
| hsa-miR-1207-5p | 796-802 | 7mer-m8 | -0.02 | 36 | -0.02 | 0 | N/A |
| hsa-miR-4763-3p | 796-802 | 7mer-m8 | -0.02 | 35 | -0.02 | 0 | N/A |
| hsa-miR-6721-5p | 797-803 | 7mer-m8 | -0.02 | 29 | -0.02 | 0 | N/A |
| hsa-miR-4632-5p | 798-804 | 7mer-m8 | -0.02 | 40 | -0.02 | 0.310 | N/A |
| hsa-miR-7843-5p | 798-804 | 7mer-m8 | -0.02 | 39 | -0.02 | 0.310 | N/A |
| hsa-miR-6879-5p | 798-804 | 7mer-m8 | -0.02 | 31 | -0.02 | 0.310 | N/A |
| hsa-miR-6735-5p | 798-804 | 7mer-m8 | -0.02 | 30 | -0.02 | 0.310 | N/A |
| hsa-miR-4436b-3p | 798-804 | 7mer-m8 | -0.02 | 30 | -0.02 | 0.310 | N/A |
| hsa-miR-6837-5p | 800-806 | 7mer-m8 | -0.03 | 42 | -0.03 | 0 | N/A |
| hsa-miR-4685-5p | 800-806 | 7mer-m8 | -0.02 | 40 | -0.02 | 0 | N/A |
| hsa-miR-1972 | 804-810 | 7mer-m8 | -0.04 | 45 | -0.04 | 0.072 | N/A |
| hsa-miR-140-3p.1 | 807-813 | 7mer-m8 | -0.02 | 41 | -0.02 | 0.201 | N/A |
| hsa-miR-140-3p.2 | 808-814 | 7mer-m8 | -0.02 | 39 | -0.02 | 0.201 | N/A |
| hsa-miR-4786-5p | 811-818 | 8mer | -0.03 | 33 | -0.03 | 0 | N/A |
| hsa-miR-769-5p | 812-818 | 7mer-1A | -0.01 | 25 | -0.01 | 0.072 | N/A |
| hsa-miR-4329 | 813-819 | 7mer-m8 | -0.02 | 58 | -0.02 | 0 | N/A |
| hsa-miR-647 | 817-823 | 7mer-1A | -0.04 | 71 | -0.04 | 0 | N/A |
| hsa-miR-6842-3p | 817-823 | 7mer-1A | -0.1 | 67 | -0.1 | 0.468 | N/A |
| hsa-miR-6809-3p | 822-828 | 7mer-m8 | -0.02 | 49 | -0.02 | 0 | N/A |
| hsa-miR-4753-3p | 822-828 | 7mer-1A | -0.01 | 28 | -0.01 | 0 | N/A |
| hsa-miR-942-5p | 823-829 | 7mer-m8 | -0.02 | 38 | -0.02 | 0.021 | N/A |
| hsa-miR-6868-3p | 825-831 | 7mer-m8 | -0.02 | 38 | -0.02 | 0 | N/A |
| hsa-miR-1237-3p | 825-831 | 7mer-1A | -0.01 | 30 | -0.01 | 0.021 | N/A |
| hsa-miR-1248 | 825-831 | 7mer-1A | -0.01 | 27 | -0.01 | 0.021 | N/A |
| hsa-miR-4448 | 827-833 | 7mer-m8 | -0.02 | 45 | -0.02 | 0 | N/A |
| hsa-miR-5088-5p | 830-836 | 7mer-m8 | -0.16 | 85 | -0.16 | 0 | N/A |
| hsa-miR-6864-5p | 833-840 | 8mer | -0.08 | 68 | -0.08 | 0 | N/A |
| hsa-miR-3179 | 833-839 | 7mer-1A | -0.04 | 47 | -0.04 | 0 | N/A |
| hsa-miR-7515 | 833-839 | 7mer-1A | -0.04 | 36 | -0.04 | 0 | N/A |
| hsa-miR-6126 | 834-840 | 7mer-1A | -0.01 | 48 | -0.01 | 0 | N/A |
| hsa-miR-4635 | 835-842 | 8mer | -0.03 | 68 | -0.03 | 0 | N/A |
| hsa-miR-526b-5p | 837-843 | 7mer-1A | -0.05 | 61 | -0.05 | 0.075 | N/A |
| hsa-miR-103a-2-5p | 839-845 | 7mer-m8 | -0.02 | 33 | -0.02 | 0.072 | N/A |
| hsa-miR-5008-5p | 847-854 | 8mer | -0.21 | 89 | -0.21 | 0 | N/A |
| hsa-miR-6089 | 848-854 | 7mer-1A | -0.01 | 39 | -0.01 | 0 | N/A |
| hsa-miR-4316 | 849-855 | 7mer-m8 | -0.03 | 55 | -0.03 | 0 | N/A |
| hsa-miR-4710 | 850-857 | 8mer | -0.03 | 48 | -0.03 | 0 | N/A |
| hsa-miR-7855-5p | 851-858 | 8mer | -0.03 | 45 | -0.03 | 0 | N/A |
| hsa-miR-4792 | 851-857 | 7mer-1A | -0.02 | 39 | -0.02 | 0 | N/A |
| hsa-miR-3189-3p | 855-861 | 7mer-1A | -0.02 | 47 | -0.02 | 0 | N/A |
| hsa-miR-1911-5p | 862-868 | 7mer-m8 | -0.17 | 81 | -0.17 | 0 | N/A |
| hsa-miR-3944-5p | 869-876 | 8mer | -0.23 | 93 | -0.23 | 0 | N/A |
| hsa-miR-143-5p | 870-876 | 7mer-1A | -0.01 | 28 | -0.01 | 0.468 | N/A |
| hsa-miR-6867-5p | 873-880 | 8mer | -0.18 | 69 | -0.18 | 0 | N/A |
| hsa-miR-6867-5p | 875-882 | 8mer | -0.23 | 79 | -0.23 | 0 | N/A |
| hsa-miR-3182 | 880-886 | 7mer-m8 | -0.02 | 59 | -0.02 | 0 | N/A |
| hsa-miR-1237-3p | 881-888 | 8mer | -0.15 | 89 | -0.15 | 0.021 | N/A |
| hsa-miR-6868-3p | 882-888 | 7mer-m8 | -0.07 | 70 | -0.07 | 0 | N/A |
| hsa-miR-1248 | 882-888 | 7mer-1A | -0.06 | 67 | -0.06 | 0.312 | N/A |
| hsa-miR-660-3p | 885-891 | 7mer-1A | -0.1 | 71 | -0.1 | 0.072 | N/A |
| hsa-miR-5193 | 885-891 | 7mer-1A | -0.08 | 70 | -0.08 | 0.273 | N/A |
| hsa-miR-4268 | 887-894 | 8mer | -0.23 | 94 | -0.23 | 0 | N/A |
| hsa-miR-6772-3p | 888-894 | 7mer-m8 | -0.14 | 81 | -0.14 | 0 | N/A |
| hsa-miR-4448 | 888-894 | 7mer-1A | -0.07 | 79 | -0.07 | 0 | N/A |
| hsa-miR-6828-3p | 890-896 | 7mer-m8 | -0.06 | 66 | -0.06 | 0 | N/A |
| hsa-miR-767-3p | 890-896 | 7mer-1A | -0.06 | 61 | -0.06 | 0.072 | N/A |
| hsa-miR-6728-3p | 891-897 | 7mer-m8 | -0.05 | 67 | -0.05 | 0 | N/A |
| hsa-miR-3194-3p | 892-898 | 7mer-m8 | -0.16 | 87 | -0.16 | 0 | N/A |
| hsa-miR-6796-3p | 894-900 | 7mer-m8 | -0.02 | 47 | -0.02 | 0 | N/A |
| hsa-miR-6806-3p | 895-901 | 7mer-m8 | -0.07 | 74 | -0.07 | 0.072 | N/A |
| hsa-miR-3928-5p | 895-901 | 7mer-m8 | -0.06 | 71 | -0.06 | 0.072 | N/A |
| hsa-miR-1286 | 900-906 | 7mer-1A | -0.03 | 57 | -0.03 | 0.021 | N/A |
| hsa-miR-4722-5p | 900-906 | 7mer-1A | -0.01 | 26 | -0.01 | 0 | N/A |
| hsa-miR-1205 | 901-908 | 8mer | -0.34 | 98 | -0.34 | 0.021 | N/A |
| hsa-miR-3158-5p | 902-908 | 7mer-1A | -0.04 | 65 | -0.04 | 0.445 | N/A |
| hsa-miR-1184 | 902-908 | 7mer-1A | -0.01 | 44 | -0.01 | 0.075 | N/A |
| hsa-miR-17-3p | 902-908 | 7mer-1A | -0.01 | 35 | -0.01 | 0.507 | N/A |
| hsa-miR-544a | 903-909 | 7mer-m8 | -0.02 | 23 | -0.02 | 0.075 | N/A |
| hsa-miR-7158-3p | 909-915 | 7mer-1A | -0.08 | 79 | -0.08 | 0 | N/A |
| hsa-miR-203b-3p | 909-915 | 7mer-1A | -0.05 | 68 | -0.05 | 0 | N/A |
| hsa-miR-6844 | 913-919 | 7mer-1A | -0.08 | 80 | -0.08 | 0 | N/A |
| hsa-miR-4682 | 927-933 | 7mer-m8 | -0.12 | 86 | -0.12 | 0 | N/A |
| hsa-miR-8077 | 928-935 | 8mer | -0.31 | 98 | -0.31 | 0 | N/A |
| hsa-miR-7160-5p | 929-935 | 7mer-1A | -0.15 | 86 | -0.15 | 0 | N/A |
| hsa-miR-4663 | 929-935 | 7mer-1A | -0.09 | 81 | -0.09 | 0 | N/A |
| hsa-miR-4728-3p | 930-936 | 7mer-m8 | -0.15 | 86 | -0.15 | 0 | N/A |
| hsa-miR-6124 | 936-942 | 7mer-1A | -0.01 | 49 | -0.01 | 0 | N/A |
| hsa-miR-3148 | 936-942 | 7mer-1A | -0.01 | 25 | -0.01 | 0 | N/A |
| hsa-miR-875-3p | 937-943 | 7mer-m8 | -0.11 | 84 | -0.11 | 0.072 | N/A |
| hsa-miR-4515 | 940-946 | 7mer-m8 | -0.22 | 86 | -0.22 | 0 | N/A |
| hsa-miR-8052 | 941-948 | 8mer | -0.35 | 96 | -0.35 | 0 | N/A |
| hsa-miR-3199 | 941-948 | 8mer | -0.35 | 96 | -0.35 | 0 | N/A |
| hsa-miR-365a-5p | 942-948 | 7mer-1A | -0.12 | 77 | -0.12 | 0 | N/A |
| hsa-miR-365b-5p | 942-948 | 7mer-1A | -0.12 | 76 | -0.12 | 0 | N/A |
| hsa-miR-4648 | 943-949 | 7mer-m8 | -0.33 | 97 | -0.33 | 0 | N/A |
| hsa-miR-194-3p | 945-951 | 7mer-m8 | -0.16 | 89 | -0.16 | 0 | N/A |
| hsa-miR-548au-3p | 948-954 | 7mer-1A | -0.07 | 63 | -0.07 | 0 | N/A |
| hsa-miR-5197-5p | 950-957 | 8mer | -0.16 | 83 | -0.16 | 0 | N/A |
| hsa-miR-5697 | 958-964 | 7mer-m8 | -0.14 | 89 | -0.14 | 0 | N/A |
| hsa-miR-578 | 965-971 | 7mer-1A | -0.03 | 67 | -0.03 | 0.310 | N/A |
| hsa-miR-210-5p | 978-984 | 7mer-1A | -0.08 | 58 | -0.08 | 0.072 | N/A |
| hsa-miR-4749-3p | 978-984 | 7mer-1A | -0.03 | 46 | -0.03 | 0 | N/A |
| hsa-miR-1913 | 979-985 | 7mer-m8 | -0.13 | 80 | -0.13 | 0.075 | N/A |
| hsa-miR-324-3p | 979-985 | 7mer-m8 | -0.12 | 78 | -0.12 | 0.075 | N/A |
| hsa-miR-647 | 982-989 | 8mer | -0.12 | 87 | -0.12 | 0 | N/A |
| hsa-miR-6762-3p | 982-988 | 7mer-1A | -0.17 | 84 | -0.17 | 0 | N/A |
| hsa-miR-604 | 982-988 | 7mer-1A | -0.08 | 67 | -0.08 | 0.021 | N/A |
| hsa-miR-6842-3p | 983-989 | 7mer-1A | -0.07 | 59 | -0.07 | 0 | N/A |
| hsa-miR-3614-5p | 986-993 | 8mer | -0.03 | 50 | -0.03 | 0 | N/A |
| hsa-miR-6500-3p | 987-993 | 7mer-1A | -0.01 | 26 | -0.01 | 0 | N/A |
| hsa-miR-548au-3p | 997-1003 | 7mer-m8 | -0.06 | 55 | -0.06 | 0 | N/A |
| hsa-miR-486-3p | 999-1005 | 7mer-m8 | -0.16 | 63 | -0.16 | 0.505 | N/A |
| hsa-miR-6743-5p | 1000-1006 | 7mer-m8 | -0.06 | 44 | -0.06 | 0 | N/A |
| hsa-miR-4688 | 1000-1006 | 7mer-m8 | -0.06 | 41 | -0.06 | 0 | N/A |
| hsa-miR-7113-5p | 1003-1009 | 7mer-1A | -0.09 | 69 | -0.09 | 0 | N/A |
| hsa-miR-6837-5p | 1003-1009 | 7mer-1A | -0.01 | 32 | -0.01 | 0 | N/A |
| hsa-miR-4685-5p | 1003-1009 | 7mer-1A | -0.01 | 31 | -0.01 | 0 | N/A |
| hsa-miR-6723-5p | 1007-1013 | 7mer-1A | -0.16 | 84 | -0.16 | 0 | N/A |
| hsa-miR-4540 | 1007-1013 | 7mer-1A | -0.08 | 63 | -0.08 | 0.625 | N/A |
| hsa-miR-6731-3p | 1025-1032 | 8mer | -0.15 | 86 | -0.15 | 0 | N/A |
| hsa-miR-26a-1-3p | 1026-1032 | 7mer-1A | -0.05 | 58 | -0.05 | 0.452 | N/A |
| hsa-miR-26a-2-3p | 1026-1032 | 7mer-1A | -0.05 | 58 | -0.05 | 0.452 | N/A |
| hsa-miR-4761-5p | 1035-1041 | 7mer-m8 | -0.2 | 92 | -0.2 | 0 | N/A |
| hsa-miR-1266-3p | 1048-1054 | 7mer-1A | -0.12 | 80 | -0.12 | 0 | N/A |
| hsa-miR-7152-5p | 1049-1056 | 8mer | -0.2 | 95 | -0.2 | 0 | N/A |
| hsa-miR-3074-5p | 1050-1056 | 7mer-1A | -0.04 | 81 | -0.04 | 0 | N/A |
| hsa-miR-3124-3p | 1051-1058 | 8mer | -0.13 | 88 | -0.13 | 0 | N/A |
| hsa-miR-4677-5p | 1055-1061 | 7mer-m8 | -0.09 | 81 | -0.09 | 0.072 | N/A |
| hsa-miR-2116-5p | 1055-1061 | 7mer-1A | -0.06 | 73 | -0.06 | 0 | N/A |
| hsa-miR-22-5p | 1055-1061 | 7mer-1A | -0.04 | 55 | -0.04 | 0.468 | N/A |
| hsa-miR-26b-3p | 1056-1063 | 8mer | -0.26 | 94 | -0.26 | 0.072 | N/A |
| hsa-miR-4652-3p | 1058-1065 | 8mer | -0.03 | 75 | -0.03 | 0 | N/A |
| hsa-miR-4743-3p | 1059-1065 | 7mer-1A | -0.03 | 70 | -0.03 | 0 | N/A |
| hsa-miR-8058 | 1070-1077 | 8mer | -0.19 | 89 | -0.19 | 0.347 | N/A |
| hsa-miR-5009-5p | 1070-1077 | 8mer | -0.17 | 86 | -0.17 | 0.347 | N/A |
| hsa-miR-1269b | 1071-1077 | 7mer-1A | -0.16 | 80 | -0.16 | 0.310 | N/A |
| hsa-miR-1269a | 1071-1077 | 7mer-1A | -0.15 | 79 | -0.15 | 0.310 | N/A |
| hsa-miR-4529-3p | 1071-1077 | 7mer-m8 | -0.13 | 69 | -0.13 | 0 | N/A |
| hsa-miR-4748 | 1084-1090 | 7mer-m8 | -0.02 | 57 | -0.02 | 0 | N/A |
| hsa-miR-4464 | 1084-1090 | 7mer-m8 | -0.02 | 42 | -0.02 | 0 | N/A |
| hsa-miR-516a-5p | 1089-1095 | 7mer-m8 | -0.16 | 79 | -0.16 | 0.072 | N/A |
| hsa-miR-627-5p | 1093-1099 | 7mer-m8 | -0.02 | 41 | -0.02 | 0.075 | N/A |
| hsa-miR-924 | 1093-1099 | 7mer-1A | -0.01 | 22 | -0.01 | 0.075 | N/A |
| hsa-miR-1245b-3p | 1097-1104 | 8mer | -0.03 | 44 | -0.03 | 0 | N/A |
| hsa-miR-4330 | 1099-1106 | 8mer | -0.03 | 63 | -0.03 | 0 | N/A |
| hsa-miR-3907 | 1105-1111 | 7mer-m8 | -0.13 | 75 | -0.13 | 0 | N/A |
| hsa-miR-1250-5p | 1106-1112 | 7mer-m8 | -0.23 | 74 | -0.23 | 0.072 | N/A |
| hsa-miR-361-3p | 1116-1122 | 7mer-m8 | -0.02 | 27 | -0.02 | 0.075 | N/A |
| hsa-miR-296-5p | 1120-1126 | 7mer-m8 | -0.11 | 61 | -0.11 | 1.616 | N/A |
| hsa-miR-602 | 1124-1130 | 7mer-m8 | -0.02 | 19 | -0.02 | 0.021 | N/A |
| hsa-miR-4462 | 1125-1131 | 7mer-m8 | -0.02 | 5 | -0.02 | 0 | N/A |
| hsa-miR-6781-5p | 1135-1141 | 7mer-m8 | -0.04 | 16 | -0.04 | 0 | N/A |
| hsa-miR-128-2-5p | 1136-1142 | 7mer-m8 | -0.02 | 34 | -0.02 | 0.072 | N/A |
| hsa-miR-128-1-5p | 1136-1142 | 7mer-m8 | -0.02 | 22 | -0.02 | 0.072 | N/A |
| hsa-miR-4781-5p | 1140-1146 | 7mer-m8 | -0.03 | 7 | -0.03 | 0 | N/A |
| hsa-miR-1226-3p | 1144-1150 | 7mer-m8 | -0.02 | 51 | -0.02 | 0.021 | N/A |
| hsa-miR-498 | 1152-1158 | 7mer-m8 | -0.02 | 69 | -0.02 | 0.075 | N/A |
| hsa-miR-7974 | 1162-1168 | 7mer-m8 | -0.04 | 35 | -0.04 | 0 | N/A |
| hsa-miR-1911-3p | 1166-1172 | 7mer-m8 | -0.17 | 83 | -0.17 | 0 | N/A |
| hsa-miR-6804-5p | 1173-1180 | 8mer | -0.2 | 79 | -0.2 | 0 | N/A |
| hsa-miR-4649-3p | 1176-1182 | 7mer-1A | -0.01 | 41 | -0.01 | 0 | N/A |
| hsa-miR-7162-3p | 1176-1182 | 7mer-1A | -0.01 | 41 | -0.01 | 0 | N/A |
| hsa-miR-187-3p | 1180-1186 | 7mer-m8 | -0.21 | 72 | -0.21 | 0.405 | N/A |
| hsa-miR-92b-5p | 1185-1191 | 7mer-1A | -0.25 | 71 | -0.25 | 0.072 | N/A |
| hsa-miR-4515 | 1189-1196 | 8mer | -0.16 | 76 | -0.16 | 0 | N/A |
| hsa-miR-4740-5p | 1190-1196 | 7mer-1A | -0.11 | 67 | -0.11 | 0 | N/A |
| hsa-miR-1269a | 1190-1196 | 7mer-m8 | -0.07 | 58 | -0.07 | 0.609 | N/A |
| hsa-miR-1269b | 1190-1196 | 7mer-m8 | -0.03 | 40 | -0.03 | 0.609 | N/A |
| hsa-miR-1288-3p | 1190-1196 | 7mer-1A | -0.02 | 38 | -0.02 | 0.075 | N/A |
| hsa-miR-1207-3p | 1194-1200 | 7mer-m8 | -0.02 | 43 | -0.02 | 1.107 | N/A |
| hsa-miR-6721-5p | 1198-1204 | 7mer-1A | -0.01 | 21 | -0.01 | 0.310 | N/A |
| hsa-miR-612 | 1199-1205 | 7mer-m8 | -0.02 | 39 | -0.02 | 0.021 | N/A |
| hsa-miR-3187-5p | 1199-1205 | 7mer-m8 | -0.02 | 28 | -0.02 | 0.021 | N/A |
| hsa-miR-5189-5p | 1199-1205 | 7mer-m8 | -0.02 | 28 | -0.02 | 0.021 | N/A |
| hsa-miR-1285-3p | 1199-1205 | 7mer-m8 | -0.02 | 26 | -0.02 | 0.021 | N/A |
| hsa-miR-6860 | 1199-1205 | 7mer-m8 | -0.02 | 25 | -0.02 | 0.021 | N/A |
| hsa-miR-4505 | 1201-1207 | 7mer-m8 | -0.13 | 73 | -0.13 | 0 | N/A |
| hsa-miR-5787 | 1201-1207 | 7mer-m8 | -0.06 | 65 | -0.06 | 0 | N/A |
| hsa-miR-3177-3p | 1205-1211 | 7mer-m8 | -0.24 | 82 | -0.24 | 0 | N/A |
| hsa-miR-564 | 1206-1212 | 7mer-m8 | -0.18 | 63 | -0.18 | 0.021 | N/A |
| hsa-miR-5681a | 1211-1217 | 7mer-m8 | -0.02 | 40 | -0.02 | 0 | N/A |
| hsa-miR-3153 | 1213-1219 | 7mer-m8 | -0.02 | 54 | -0.02 | 0 | N/A |
| hsa-miR-6733-5p | 1213-1219 | 7mer-m8 | -0.02 | 42 | -0.02 | 0 | N/A |
| hsa-miR-6739-5p | 1213-1219 | 7mer-m8 | -0.02 | 41 | -0.02 | 0 | N/A |
| hsa-miR-3196 | 1218-1225 | 8mer | -0.48 | 93 | -0.48 | 0 | N/A |
| hsa-miR-6816-5p | 1218-1225 | 8mer | -0.44 | 92 | -0.44 | 0 | N/A |
| hsa-miR-3180 | 1218-1225 | 8mer | -0.43 | 92 | -0.43 | 0 | N/A |
| hsa-miR-3180-3p | 1218-1225 | 8mer | -0.43 | 92 | -0.43 | 0 | N/A |
| hsa-miR-183-5p.2 | 1225-1231 | 7mer-m8 | -0.03 | 40 | -0.03 | 1.221 | N/A |
| hsa-miR-5580-5p | 1228-1234 | 7mer-1A | -0.06 | 48 | -0.06 | 0 | N/A |
| hsa-miR-3692-5p | 1229-1235 | 7mer-m8 | -0.06 | 66 | -0.06 | 0 | N/A |
| hsa-miR-320e | 1233-1239 | 7mer-1A | -0.05 | 64 | -0.05 | 0 | N/A |
| hsa-miR-34a-5p | 1242-1248 | 7mer-m8 | -0.16 | 69 | -0.16 | 1.082 | N/A |
| hsa-miR-34c-5p | 1242-1248 | 7mer-m8 | -0.14 | 63 | -0.14 | 1.082 | N/A |
| hsa-miR-449a | 1242-1248 | 7mer-m8 | -0.11 | 60 | -0.11 | 1.082 | N/A |
| hsa-miR-449b-5p | 1242-1248 | 7mer-m8 | -0.11 | 59 | -0.11 | 1.082 | N/A |
| hsa-miR-7112-5p | 1244-1250 | 7mer-m8 | -0.34 | 91 | -0.34 | 0 | N/A |
| hsa-miR-602 | 1247-1253 | 7mer-m8 | -0.22 | 87 | -0.22 | 0.021 | N/A |
| hsa-miR-4261 | 1251-1258 | 8mer | -0.03 | 39 | -0.03 | 0 | N/A |
| hsa-miR-875-3p | 1253-1259 | 7mer-m8 | -0.02 | 33 | -0.02 | 0.072 | N/A |
| hsa-miR-6791-5p | 1262-1268 | 7mer-1A | -0.05 | 50 | -0.05 | 0 | N/A |
| hsa-miR-4292 | 1262-1268 | 7mer-1A | -0.01 | 23 | -0.01 | 0 | N/A |
| hsa-miR-4308 | 1262-1268 | 7mer-1A | -0.01 | 20 | -0.01 | 0.409 | N/A |
| hsa-miR-2114-5p | 1264-1270 | 7mer-m8 | -0.02 | 32 | -0.02 | 0 | N/A |
| hsa-miR-3064-3p | 1270-1276 | 7mer-m8 | -0.02 | 23 | -0.02 | 0 | N/A |
| hsa-miR-6882-3p | 1273-1279 | 7mer-1A | -0.05 | 47 | -0.05 | 0 | N/A |
| hsa-miR-4664-5p | 1277-1283 | 7mer-m8 | -0.09 | 74 | -0.09 | 0 | N/A |
| hsa-miR-342-5p | 1277-1283 | 7mer-m8 | -0.08 | 66 | -0.08 | 0 | N/A |
| hsa-miR-6752-5p | 1278-1284 | 7mer-m8 | -0.06 | 35 | -0.06 | 0 | N/A |
| hsa-miR-7110-5p | 1278-1284 | 7mer-m8 | -0.08 | 33 | -0.08 | 0 | N/A |
| hsa-miR-6842-5p | 1278-1284 | 7mer-m8 | -0.08 | 32 | -0.08 | 0 | N/A |
| hsa-miR-4787-5p | 1279-1285 | 7mer-m8 | -0.07 | 44 | -0.07 | 0 | N/A |
| hsa-miR-3689d | 1287-1293 | 7mer-m8 | -0.02 | 35 | -0.02 | 0 | N/A |
| hsa-miR-6851-5p | 1287-1293 | 7mer-m8 | -0.02 | 22 | -0.02 | 0 | N/A |
| hsa-miR-6799-5p | 1288-1294 | 7mer-m8 | -0.02 | 53 | -0.02 | 0 | N/A |
| hsa-miR-6825-5p | 1289-1296 | 8mer | -0.09 | 44 | -0.09 | 0 | N/A |
| hsa-miR-6763-5p | 1290-1296 | 7mer-m8 | -0.03 | 44 | -0.03 | 0.072 | N/A |
| hsa-miR-3150a-3p | 1290-1296 | 7mer-m8 | -0.02 | 33 | -0.02 | 0.072 | N/A |
| hsa-miR-3175 | 1290-1296 | 7mer-1A | -0.04 | 32 | -0.04 | 0 | N/A |
| hsa-miR-1343-5p | 1290-1296 | 7mer-1A | -0.01 | 28 | -0.01 | 0.075 | N/A |
| hsa-miR-939-5p | 1290-1296 | 7mer-1A | -0.01 | 24 | -0.01 | 0.075 | N/A |
| hsa-miR-491-5p | 1291-1297 | 7mer-m8 | -0.02 | 11 | -0.02 | 1.016 | N/A |
| hsa-miR-6853-5p | 1293-1299 | 7mer-m8 | -0.18 | 72 | -0.18 | 0 | N/A |
| hsa-miR-320e | 1299-1305 | 7mer-m8 | -0.06 | 68 | -0.06 | 0 | N/A |
| hsa-miR-127-5p | 1301-1307 | 7mer-m8 | -0.03 | 46 | -0.03 | 0 | N/A |
| hsa-miR-3928-5p | 1301-1307 | 7mer-1A | -0.01 | 34 | -0.01 | 0 | N/A |
| hsa-miR-6806-3p | 1301-1307 | 7mer-1A | -0.01 | 33 | -0.01 | 0 | N/A |
| hsa-miR-3611 | 1303-1309 | 7mer-m8 | -0.02 | 44 | -0.02 | 0 | N/A |
| hsa-miR-6715b-5p | 1310-1316 | 7mer-m8 | -0.05 | 72 | -0.05 | 0 | N/A |
| hsa-miR-4269 | 1310-1316 | 7mer-m8 | -0.02 | 65 | -0.02 | 0 | N/A |
| hsa-miR-4690-5p | 1312-1318 | 7mer-m8 | -0.05 | 77 | -0.05 | 0 | N/A |
| hsa-miR-761 | 1313-1319 | 7mer-m8 | -0.02 | 55 | -0.02 | 0.021 | N/A |
| hsa-miR-3619-5p | 1313-1319 | 7mer-m8 | -0.02 | 46 | -0.02 | 0.021 | N/A |
| hsa-miR-214-3p | 1313-1319 | 7mer-m8 | -0.02 | 43 | -0.02 | 0.021 | N/A |
| hsa-miR-6851-5p | 1329-1335 | 7mer-1A | -0.03 | 29 | -0.03 | 0 | N/A |
| hsa-miR-3689d | 1329-1335 | 7mer-1A | -0.01 | 26 | -0.01 | 0 | N/A |
| hsa-miR-3135b | 1331-1338 | 8mer | -0.03 | 50 | -0.03 | 0 | N/A |
| hsa-miR-1287-5p | 1332-1338 | 7mer-1A | -0.01 | 22 | -0.01 | 0 | N/A |
| hsa-miR-3907 | 1335-1341 | 7mer-1A | -0.01 | 18 | -0.01 | 0 | N/A |
| hsa-miR-548q | 1336-1342 | 7mer-m8 | -0.14 | 91 | -0.14 | 0 | N/A |
| hsa-miR-582-3p | 1338-1344 | 7mer-m8 | -0.02 | 36 | -0.02 | 0.072 | N/A |
| hsa-miR-1288-3p | 1346-1353 | 8mer | -0.26 | 92 | -0.26 | 0.075 | N/A |
| hsa-miR-4740-5p | 1347-1353 | 7mer-1A | -0.12 | 71 | -0.12 | 0 | N/A |
| hsa-miR-1269a | 1347-1353 | 7mer-m8 | -0.11 | 70 | -0.11 | 0 | N/A |
| hsa-miR-1269b | 1347-1353 | 7mer-m8 | -0.09 | 64 | -0.09 | 0 | N/A |
| hsa-miR-4515 | 1347-1353 | 7mer-1A | -0.06 | 51 | -0.06 | 0 | N/A |
| hsa-miR-378c | 1348-1354 | 7mer-m8 | -0.06 | 58 | -0.06 | 0.735 | N/A |
| hsa-miR-378b | 1348-1354 | 7mer-m8 | -0.06 | 58 | -0.06 | 0.735 | N/A |
| hsa-miR-422a | 1348-1354 | 7mer-m8 | -0.06 | 58 | -0.06 | 0.735 | N/A |
| hsa-miR-378h | 1348-1354 | 7mer-m8 | -0.06 | 58 | -0.06 | 0.735 | N/A |
| hsa-miR-378i | 1348-1354 | 7mer-m8 | -0.05 | 52 | -0.05 | 0.735 | N/A |
| hsa-miR-378d | 1348-1354 | 7mer-m8 | -0.05 | 52 | -0.05 | 0.735 | N/A |
| hsa-miR-378f | 1348-1354 | 7mer-m8 | -0.05 | 52 | -0.05 | 0.735 | N/A |
| hsa-miR-378e | 1348-1354 | 7mer-m8 | -0.05 | 52 | -0.05 | 0.735 | N/A |
| hsa-miR-378a-3p | 1348-1354 | 7mer-m8 | -0.05 | 52 | -0.05 | 0.735 | N/A |
| hsa-miR-4430 | 1350-1356 | 7mer-m8 | -0.07 | 67 | -0.07 | 0 | N/A |
| hsa-miR-3652 | 1350-1356 | 7mer-m8 | -0.06 | 60 | -0.06 | 0 | N/A |
| hsa-miR-4498 | 1351-1357 | 7mer-m8 | -0.27 | 89 | -0.27 | 0.075 | N/A |
| hsa-miR-4492 | 1351-1357 | 7mer-m8 | -0.16 | 86 | -0.16 | 0.075 | N/A |
| hsa-miR-5001-5p | 1351-1357 | 7mer-m8 | -0.21 | 83 | -0.21 | 0.075 | N/A |
| hsa-miR-762 | 1351-1357 | 7mer-m8 | -0.14 | 79 | -0.14 | 0.075 | N/A |
| hsa-miR-185-3p | 1352-1358 | 7mer-m8 | -0.22 | 78 | -0.22 | 0.445 | N/A |
| hsa-miR-6848-5p | 1353-1360 | 8mer | -0.26 | 83 | -0.26 | 0 | N/A |
| hsa-miR-6846-5p | 1353-1360 | 8mer | -0.25 | 83 | -0.25 | 0 | N/A |
| hsa-miR-4697-5p | 1354-1360 | 7mer-1A | -0.24 | 84 | -0.24 | 0 | N/A |
| hsa-miR-1237-5p | 1354-1360 | 7mer-1A | -0.21 | 80 | -0.21 | 0 | N/A |
| hsa-miR-4488 | 1354-1360 | 7mer-1A | -0.2 | 77 | -0.2 | 0 | N/A |
| hsa-miR-4447 | 1355-1361 | 7mer-m8 | -0.1 | 72 | -0.1 | 0 | N/A |
| hsa-miR-4472 | 1355-1361 | 7mer-m8 | -0.1 | 72 | -0.1 | 0 | N/A |
| hsa-miR-92a-2-5p | 1356-1362 | 7mer-m8 | -0.2 | 90 | -0.2 | 0.072 | N/A |
| hsa-miR-6730-5p | 1360-1366 | 7mer-m8 | -0.02 | 42 | -0.02 | 0 | N/A |
| hsa-miR-6733-5p | 1362-1368 | 7mer-m8 | -0.05 | 64 | -0.05 | 0 | N/A |
| hsa-miR-3153 | 1362-1368 | 7mer-m8 | -0.02 | 54 | -0.02 | 0 | N/A |
| hsa-miR-6739-5p | 1362-1368 | 7mer-m8 | -0.02 | 41 | -0.02 | 0 | N/A |
| hsa-miR-5584-5p | 1363-1369 | 7mer-m8 | -0.02 | 30 | -0.02 | 0 | N/A |
| hsa-miR-1207-5p | 1366-1372 | 7mer-m8 | -0.23 | 90 | -0.23 | 0 | N/A |
| hsa-miR-4763-3p | 1366-1372 | 7mer-m8 | -0.21 | 88 | -0.21 | 0 | N/A |
| hsa-miR-6721-5p | 1367-1373 | 7mer-m8 | -0.13 | 71 | -0.13 | 0 | N/A |
| hsa-miR-486-3p | 1368-1374 | 7mer-m8 | -0.2 | 73 | -0.2 | 0.505 | N/A |
| hsa-miR-6743-5p | 1369-1375 | 7mer-m8 | -0.19 | 83 | -0.19 | 0.409 | N/A |
| hsa-miR-4688 | 1369-1375 | 7mer-m8 | -0.19 | 82 | -0.19 | 0.409 | N/A |
| hsa-miR-4659b-5p | 1387-1393 | 7mer-m8 | -0.08 | 68 | -0.08 | 0 | N/A |
| hsa-miR-4659a-5p | 1387-1393 | 7mer-m8 | -0.06 | 62 | -0.06 | 0 | N/A |
| hsa-miR-1229-5p | 1400-1406 | 7mer-m8 | -0.11 | 75 | -0.11 | 0 | N/A |
| hsa-miR-197-5p | 1400-1406 | 7mer-1A | -0.18 | 69 | -0.18 | 0.021 | N/A |
| hsa-miR-3132 | 1400-1406 | 7mer-1A | -0.15 | 66 | -0.15 | 0.021 | N/A |
| hsa-miR-6861-5p | 1401-1407 | 7mer-m8 | -0.13 | 81 | -0.13 | 0 | N/A |
| hsa-miR-582-3p | 1404-1410 | 7mer-1A | -0.03 | 43 | -0.03 | 0 | N/A |
| hsa-miR-1252-5p | 1411-1417 | 7mer-m8 | -0.02 | 20 | -0.02 | 0.021 | N/A |
| hsa-miR-4533 | 1412-1418 | 7mer-m8 | -0.07 | 65 | -0.07 | 0 | N/A |
| hsa-miR-6758-5p | 1412-1423 | non-canonical | N/A | N/A | N/A | 0 | N/A |
| hsa-miR-6758-5p | 1412-1423 | non-canonical | N/A | N/A | N/A | 0 | N/A |
| hsa-miR-4747-5p | 1413-1419 | 7mer-m8 | -0.14 | 76 | -0.14 | 0 | N/A |
| hsa-miR-5196-5p | 1413-1419 | 7mer-m8 | -0.14 | 75 | -0.14 | 0 | N/A |
| hsa-miR-4271 | 1414-1420 | 7mer-m8 | -0.1 | 71 | -0.1 | 0 | N/A |
| hsa-miR-4725-3p | 1414-1420 | 7mer-m8 | -0.12 | 68 | -0.12 | 0 | N/A |
| hsa-miR-6780b-5p | 1414-1420 | 7mer-m8 | -0.12 | 67 | -0.12 | 0 | N/A |
| hsa-miR-6783-5p | 1415-1421 | 7mer-m8 | -0.11 | 74 | -0.11 | 0 | N/A |
| hsa-miR-615-5p | 1425-1432 | 8mer | -0.08 | 59 | -0.08 | 0.072 | N/A |
| hsa-miR-7704 | 1426-1432 | 7mer-1A | -0.07 | 53 | -0.07 | 0 | N/A |
| hsa-miR-6812-5p | 1427-1433 | 7mer-1A | -0.09 | 61 | -0.09 | 0.409 | N/A |
| hsa-miR-5572 | 1427-1434 | 8mer | -0.03 | 50 | -0.03 | 0 | N/A |
| hsa-miR-6819-5p | 1427-1433 | 7mer-1A | -0.03 | 35 | -0.03 | 0.409 | N/A |
| hsa-miR-3170 | 1427-1433 | 7mer-1A | -0.01 | 21 | -0.01 | 0 | N/A |
| hsa-miR-6737-5p | 1427-1433 | 7mer-1A | -0.01 | 19 | -0.01 | 0.409 | N/A |
| hsa-miR-6855-5p | 1427-1433 | 7mer-1A | -0.01 | 17 | -0.01 | 0 | N/A |
| hsa-miR-450a-2-3p | 1428-1434 | 7mer-1A | -0.07 | 45 | -0.07 | 0.505 | N/A |
| hsa-miR-4260 | 1428-1434 | 7mer-1A | -0.01 | 26 | -0.01 | 0.409 | N/A |
| hsa-miR-6740-5p | 1429-1435 | 7mer-m8 | -0.02 | 25 | -0.02 | 0 | N/A |
| hsa-miR-4748 | 1431-1437 | 7mer-m8 | -0.02 | 57 | -0.02 | 0 | N/A |
| hsa-miR-4464 | 1431-1437 | 7mer-m8 | -0.02 | 42 | -0.02 | 0 | N/A |
| hsa-miR-657 | 1433-1439 | 7mer-m8 | -0.02 | 43 | -0.02 | 0.021 | N/A |
| hsa-miR-4736 | 1434-1440 | 7mer-m8 | -0.14 | 87 | -0.14 | 0.409 | N/A |
| hsa-miR-6721-5p | 1435-1441 | 7mer-m8 | -0.13 | 71 | -0.13 | 0 | N/A |
| hsa-miR-486-3p | 1436-1443 | 8mer | -0.25 | 82 | -0.25 | 0.072 | N/A |
| hsa-miR-6852-5p | 1439-1445 | 7mer-m8 | -0.09 | 74 | -0.09 | 0 | N/A |
| hsa-miR-661 | 1440-1446 | 7mer-m8 | -0.07 | 58 | -0.07 | 0.075 | N/A |
| hsa-miR-4493 | 1444-1450 | 7mer-m8 | -0.11 | 73 | -0.11 | 0 | N/A |
| hsa-miR-3185 | 1447-1453 | 7mer-m8 | -0.02 | 46 | -0.02 | 0 | N/A |
| hsa-miR-6780b-5p | 1450-1457 | 8mer | -0.1 | 60 | -0.1 | 0 | N/A |
| hsa-miR-4271 | 1450-1457 | 8mer | -0.04 | 52 | -0.04 | 0 | N/A |
| hsa-miR-4725-3p | 1450-1457 | 8mer | -0.07 | 50 | -0.07 | 0 | N/A |
| hsa-miR-2110 | 1451-1457 | 7mer-m8 | -0.04 | 41 | -0.04 | 0 | N/A |
| hsa-miR-4667-5p | 1452-1458 | 7mer-m8 | -0.16 | 82 | -0.16 | 0.448 | N/A |
| hsa-miR-8089 | 1452-1458 | 7mer-m8 | -0.12 | 76 | -0.12 | 0.448 | N/A |
| hsa-miR-4700-5p | 1452-1458 | 7mer-m8 | -0.1 | 72 | -0.1 | 0.448 | N/A |
| hsa-miR-4731-5p | 1453-1459 | 7mer-m8 | -0.17 | 86 | -0.17 | 0 | N/A |
| hsa-miR-4505 | 1454-1461 | 8mer | -0.37 | 97 | -0.37 | 0.409 | N/A |
| hsa-miR-5787 | 1454-1461 | 8mer | -0.29 | 97 | -0.29 | 0.409 | N/A |
| hsa-miR-4430 | 1455-1461 | 7mer-1A | -0.16 | 89 | -0.16 | 0.409 | N/A |
| hsa-miR-3652 | 1455-1461 | 7mer-1A | -0.17 | 89 | -0.17 | 0.409 | N/A |
| hsa-miR-6842-3p | 1455-1461 | 7mer-m8 | -0.11 | 71 | -0.11 | 0.468 | N/A |
| hsa-miR-6504-5p | 1456-1462 | 7mer-m8 | -0.1 | 75 | -0.1 | 2.266 | N/A |
| hsa-miR-3064-5p | 1456-1462 | 7mer-m8 | -0.09 | 72 | -0.09 | 2.266 | N/A |
| hsa-miR-5580-5p | 1457-1463 | 7mer-m8 | -0.09 | 61 | -0.09 | 0.409 | N/A |
| hsa-miR-485-5p | 1460-1467 | 8mer | -0.13 | 75 | -0.13 | 0.862 | N/A |
| hsa-miR-6884-5p | 1460-1467 | 8mer | -0.13 | 73 | -0.13 | 0.862 | N/A |
| hsa-miR-3188 | 1461-1467 | 7mer-1A | -0.06 | 73 | -0.06 | 0.468 | N/A |
| hsa-miR-3975 | 1461-1467 | 7mer-1A | -0.05 | 58 | -0.05 | 0 | N/A |
| hsa-miR-2467-5p | 1461-1467 | 7mer-1A | -0.01 | 45 | -0.01 | 0.415 | N/A |
| hsa-miR-4649-3p | 1462-1468 | 7mer-m8 | -0.17 | 91 | -0.17 | 0 | N/A |
| hsa-miR-4738-3p | 1466-1472 | 7mer-m8 | -0.06 | 71 | -0.06 | 0 | N/A |
| hsa-miR-4651 | 1477-1484 | 8mer | -0.26 | 83 | -0.26 | 0.409 | N/A |
| hsa-miR-608 | 1477-1484 | 8mer | -0.28 | 83 | -0.28 | 0.409 | N/A |
| hsa-miR-6737-5p | 1478-1484 | 7mer-m8 | -0.13 | 71 | -0.13 | 0.409 | N/A |
| hsa-miR-6812-5p | 1478-1484 | 7mer-m8 | -0.12 | 67 | -0.12 | 0.409 | N/A |
| hsa-miR-6819-5p | 1478-1484 | 7mer-m8 | -0.11 | 66 | -0.11 | 0.409 | N/A |
| hsa-miR-6747-5p | 1478-1484 | 7mer-1A | -0.08 | 60 | -0.08 | 0 | N/A |
| hsa-miR-342-5p | 1478-1484 | 7mer-1A | -0.03 | 43 | -0.03 | 0.445 | N/A |
| hsa-miR-4664-5p | 1478-1484 | 7mer-1A | -0.01 | 25 | -0.01 | 0.445 | N/A |
| hsa-miR-6742-5p | 1479-1486 | 8mer | -0.21 | 89 | -0.21 | 0 | N/A |
| hsa-miR-491-5p | 1480-1486 | 7mer-1A | -0.2 | 83 | -0.2 | 0.994 | N/A |
| hsa-miR-6796-5p | 1480-1486 | 7mer-m8 | -0.17 | 78 | -0.17 | 0.409 | N/A |
| hsa-miR-3151-5p | 1480-1486 | 7mer-1A | -0.08 | 72 | -0.08 | 0.021 | N/A |
| hsa-miR-4447 | 1480-1486 | 7mer-1A | -0.06 | 60 | -0.06 | 0 | N/A |
| hsa-miR-4472 | 1480-1486 | 7mer-1A | -0.05 | 56 | -0.05 | 0 | N/A |
| hsa-miR-574-5p | 1483-1490 | 8mer | -0.09 | 58 | -0.09 | 0 | N/A |
| hsa-miR-6790-5p | 1484-1490 | 7mer-m8 | -0.02 | 48 | -0.02 | 0 | N/A |
| hsa-miR-4658 | 1484-1490 | 7mer-m8 | -0.02 | 48 | -0.02 | 0 | N/A |
| hsa-miR-3659 | 1484-1490 | 7mer-1A | -0.02 | 21 | -0.02 | 0 | N/A |
| hsa-miR-4764-5p | 1489-1495 | 7mer-m8 | -0.11 | 64 | -0.11 | 0 | N/A |
| hsa-miR-105-3p | 1490-1497 | 8mer | -0.4 | 94 | -0.4 | 0.072 | N/A |
| hsa-miR-3917 | 1493-1499 | 7mer-1A | -0.14 | 77 | -0.14 | 0 | N/A |
| hsa-miR-4529-5p | 1498-1504 | 7mer-m8 | -0.15 | 78 | -0.15 | 0 | N/A |
| hsa-miR-15a-3p | 1499-1505 | 7mer-m8 | -0.02 | 38 | -0.02 | 0 | N/A |
| hsa-miR-2467-3p | 1503-1509 | 7mer-m8 | -0.02 | 33 | -0.02 | 0 | N/A |
| hsa-miR-1910-3p | 1505-1511 | 7mer-m8 | -0.02 | 49 | -0.02 | 0 | N/A |
| hsa-miR-6511a-5p | 1505-1511 | 7mer-m8 | -0.02 | 38 | -0.02 | 0 | N/A |
| hsa-miR-1827 | 1506-1513 | 8mer | -0.07 | 54 | -0.07 | 0.021 | N/A |
| hsa-miR-4316 | 1508-1515 | 8mer | -0.14 | 87 | -0.14 | 0 | N/A |
| hsa-miR-1294 | 1509-1515 | 7mer-1A | -0.04 | 45 | -0.04 | 0.021 | N/A |
| hsa-miR-4677-3p | 1510-1516 | 7mer-m8 | -0.03 | 52 | -0.03 | 0 | N/A |
| hsa-miR-7974 | 1512-1518 | 7mer-m8 | -0.11 | 62 | -0.11 | 0 | N/A |
| hsa-miR-484 | 1515-1521 | 7mer-1A | -0.01 | 50 | -0.01 | 0.312 | N/A |
| hsa-miR-3155b | 1515-1521 | 7mer-1A | -0.01 | 49 | -0.01 | 0.312 | N/A |
| hsa-miR-3155a | 1515-1521 | 7mer-1A | -0.01 | 48 | -0.01 | 0.312 | N/A |
| hsa-miR-510-5p | 1517-1523 | 7mer-m8 | -0.08 | 70 | -0.08 | 0 | N/A |
| hsa-miR-4471 | 1521-1527 | 7mer-m8 | -0.04 | 40 | -0.04 | 0 | N/A |
| hsa-miR-8059 | 1521-1527 | 7mer-m8 | -0.02 | 37 | -0.02 | 0 | N/A |
| hsa-miR-4725-3p | 1523-1529 | 7mer-1A | -0.02 | 28 | -0.02 | 0 | N/A |
| hsa-miR-6780b-5p | 1523-1529 | 7mer-1A | -0.02 | 27 | -0.02 | 0 | N/A |
| hsa-miR-4271 | 1523-1529 | 7mer-1A | -0.01 | 26 | -0.01 | 0 | N/A |
| hsa-miR-2110 | 1523-1529 | 7mer-m8 | -0.02 | 22 | -0.02 | 0 | N/A |
| hsa-miR-4667-5p | 1524-1530 | 7mer-m8 | -0.05 | 50 | -0.05 | 0.075 | N/A |
| hsa-miR-4700-5p | 1524-1530 | 7mer-m8 | -0.04 | 43 | -0.04 | 0.075 | N/A |
| hsa-miR-8089 | 1524-1530 | 7mer-m8 | -0.03 | 35 | -0.03 | 0.075 | N/A |
| hsa-miR-4731-5p | 1525-1531 | 7mer-m8 | -0.09 | 71 | -0.09 | 0 | N/A |
| hsa-miR-4505 | 1526-1532 | 7mer-m8 | -0.09 | 64 | -0.09 | 0 | N/A |
| hsa-miR-5787 | 1526-1532 | 7mer-m8 | -0.05 | 64 | -0.05 | 0 | N/A |
| hsa-miR-638 | 1539-1545 | 7mer-m8 | -0.17 | 79 | -0.17 | 0.072 | N/A |
| hsa-miR-6857-5p | 1541-1547 | 7mer-m8 | -0.05 | 56 | -0.05 | 0 | N/A |
| hsa-miR-4450 | 1541-1547 | 7mer-1A | -0.08 | 51 | -0.08 | 0 | N/A |
| hsa-miR-4667-5p | 1542-1549 | 8mer | -0.17 | 84 | -0.17 | 0.075 | N/A |
| hsa-miR-8089 | 1542-1549 | 8mer | -0.16 | 82 | -0.16 | 0.075 | N/A |
| hsa-miR-4700-5p | 1542-1549 | 8mer | -0.14 | 81 | -0.14 | 0.075 | N/A |
| hsa-miR-7155-5p | 1543-1549 | 7mer-1A | -0.1 | 79 | -0.1 | 0 | N/A |
| hsa-miR-637 | 1543-1549 | 7mer-1A | -0.13 | 75 | -0.13 | 0.021 | N/A |
| hsa-miR-1231 | 1545-1551 | 7mer-m8 | -0.02 | 51 | -0.02 | 0 | N/A |
| hsa-miR-3120-5p | 1546-1552 | 7mer-m8 | -0.02 | 47 | -0.02 | 0 | N/A |
| hsa-miR-6876-3p | 1547-1553 | 7mer-m8 | -0.05 | 42 | -0.05 | 0.021 | N/A |
| hsa-miR-626 | 1547-1553 | 7mer-m8 | -0.05 | 41 | -0.05 | 0.021 | N/A |
| hsa-miR-4687-3p | 1549-1555 | 7mer-1A | -0.09 | 72 | -0.09 | 0 | N/A |
| hsa-miR-7974 | 1549-1555 | 7mer-1A | -0.09 | 56 | -0.09 | 0 | N/A |
| hsa-miR-889-5p | 1550-1556 | 7mer-m8 | -0.13 | 76 | -0.13 | 0.072 | N/A |
| hsa-miR-942-3p | 1552-1558 | 7mer-m8 | -0.11 | 81 | -0.11 | 0 | N/A |
| hsa-miR-4717-3p | 1553-1559 | 7mer-m8 | -0.03 | 54 | -0.03 | 0 | N/A |
| hsa-miR-5589-3p | 1554-1560 | 7mer-m8 | -0.1 | 71 | -0.1 | 0 | N/A |
| hsa-miR-4638-3p | 1562-1568 | 7mer-m8 | -0.02 | 28 | -0.02 | 0 | N/A |
| hsa-miR-4430 | 1564-1571 | 8mer | -0.12 | 82 | -0.12 | 0 | N/A |
| hsa-miR-3652 | 1564-1571 | 8mer | -0.11 | 79 | -0.11 | 0 | N/A |
| hsa-miR-4505 | 1565-1571 | 7mer-1A | -0.17 | 82 | -0.17 | 0 | N/A |
| hsa-miR-5787 | 1565-1571 | 7mer-1A | -0.11 | 79 | -0.11 | 0 | N/A |
| hsa-miR-6842-3p | 1565-1571 | 7mer-m8 | -0.09 | 64 | -0.09 | 0 | N/A |
| hsa-miR-1538 | 1573-1580 | 8mer | -0.4 | 92 | -0.4 | 0 | N/A |
| hsa-miR-4745-3p | 1573-1580 | 8mer | -0.35 | 89 | -0.35 | 0 | N/A |
| hsa-miR-663b | 1575-1581 | 7mer-m8 | -0.05 | 60 | -0.05 | 0 | N/A |
| hsa-miR-4651 | 1578-1585 | 8mer | -0.13 | 53 | -0.13 | 0 | N/A |
| hsa-miR-608 | 1578-1585 | 8mer | -0.13 | 50 | -0.13 | 0 | N/A |
| hsa-miR-6737-5p | 1579-1585 | 7mer-m8 | -0.03 | 37 | -0.03 | 0 | N/A |
| hsa-miR-6812-5p | 1579-1585 | 7mer-m8 | -0.02 | 29 | -0.02 | 0 | N/A |
| hsa-miR-4664-5p | 1579-1585 | 7mer-1A | -0.01 | 25 | -0.01 | 0.072 | N/A |
| hsa-miR-6819-5p | 1579-1585 | 7mer-m8 | -0.02 | 25 | -0.02 | 0 | N/A |
| hsa-miR-342-5p | 1579-1585 | 7mer-1A | -0.01 | 22 | -0.01 | 0.072 | N/A |
| hsa-miR-6747-5p | 1579-1585 | 7mer-1A | -0.01 | 12 | -0.01 | 0 | N/A |
| hsa-miR-6742-5p | 1580-1586 | 7mer-m8 | -0.02 | 23 | -0.02 | 0 | N/A |
| hsa-miR-92a-2-5p | 1581-1588 | 8mer | -0.19 | 89 | -0.19 | 0.072 | N/A |
| hsa-miR-654-5p | 1582-1588 | 7mer-1A | -0.01 | 30 | -0.01 | 0.072 | N/A |
| hsa-miR-541-3p | 1582-1588 | 7mer-1A | -0.01 | 30 | -0.01 | 0.072 | N/A |
| hsa-miR-6769b-5p | 1582-1588 | 7mer-1A | -0.01 | 18 | -0.01 | 0 | N/A |
| hsa-miR-6769a-5p | 1582-1588 | 7mer-1A | -0.01 | 16 | -0.01 | 0 | N/A |
| hsa-miR-5591-3p | 1595-1601 | 7mer-m8 | -0.02 | 23 | -0.02 | 0 | N/A |
| hsa-miR-660-5p | 1595-1601 | 7mer-1A | -0.01 | 18 | -0.01 | 0.075 | N/A |
| hsa-miR-3162-3p | 1598-1604 | 7mer-m8 | -0.02 | 27 | -0.02 | 0 | N/A |
| hsa-miR-5008-5p | 1602-1608 | 7mer-m8 | -0.05 | 60 | -0.05 | 0 | N/A |
| hsa-miR-6825-5p | 1605-1612 | 8mer | -0.03 | 22 | -0.03 | 0.409 | N/A |
| hsa-miR-6763-5p | 1606-1612 | 7mer-m8 | -0.02 | 33 | -0.02 | 0.445 | N/A |
| hsa-miR-3150a-3p | 1606-1612 | 7mer-m8 | -0.02 | 33 | -0.02 | 0.445 | N/A |
| hsa-miR-1343-5p | 1606-1612 | 7mer-1A | -0.01 | 28 | -0.01 | 0.448 | N/A |
| hsa-miR-939-5p | 1606-1612 | 7mer-1A | -0.01 | 24 | -0.01 | 0.448 | N/A |
| hsa-miR-3175 | 1606-1612 | 7mer-1A | -0.01 | 10 | -0.01 | 0 | N/A |
| hsa-miR-491-5p | 1607-1613 | 7mer-m8 | -0.04 | 27 | -0.04 | 2.104 | N/A |
| hsa-miR-92a-2-5p | 1608-1614 | 7mer-m8 | -0.05 | 59 | -0.05 | 0.445 | N/A |
| hsa-miR-6786-5p | 1609-1615 | 7mer-m8 | -0.18 | 77 | -0.18 | 0 | N/A |
| hsa-miR-1276 | 1615-1621 | 7mer-m8 | -0.03 | 78 | -0.03 | 0.021 | N/A |
| hsa-miR-4766-5p | 1618-1624 | 7mer-m8 | -0.02 | 60 | -0.02 | 0 | N/A |
| hsa-miR-4740-5p | 1621-1627 | 7mer-m8 | -0.12 | 70 | -0.12 | 0 | N/A |
| hsa-miR-8052 | 1622-1628 | 7mer-m8 | -0.16 | 76 | -0.16 | 0 | N/A |
| hsa-miR-3199 | 1622-1628 | 7mer-m8 | -0.15 | 73 | -0.15 | 0 | N/A |
| hsa-miR-4734 | 1626-1632 | 7mer-m8 | -0.19 | 79 | -0.19 | 0 | N/A |
| hsa-miR-558 | 1632-1638 | 7mer-m8 | -0.12 | 83 | -0.12 | 0.075 | N/A |
| hsa-miR-6729-5p | 1636-1642 | 7mer-m8 | -0.22 | 67 | -0.22 | 0 | N/A |
| hsa-miR-4649-5p | 1636-1642 | 7mer-m8 | -0.19 | 60 | -0.19 | 0 | N/A |
| hsa-miR-1909-3p | 1640-1647 | 8mer | -0.14 | 75 | -0.14 | 0 | N/A |
| hsa-miR-6722-3p | 1640-1647 | 8mer | -0.1 | 66 | -0.1 | 0 | N/A |
| hsa-miR-6836-5p | 1641-1647 | 7mer-1A | -0.01 | 27 | -0.01 | 0.075 | N/A |
| hsa-miR-6132 | 1641-1647 | 7mer-1A | -0.01 | 23 | -0.01 | 0.075 | N/A |
| hsa-miR-1205 | 1642-1648 | 7mer-m8 | -0.09 | 80 | -0.09 | 0.021 | N/A |
| hsa-miR-558 | 1645-1652 | 8mer | -0.18 | 89 | -0.18 | 0.075 | N/A |
| hsa-miR-3160-3p | 1646-1652 | 7mer-1A | -0.1 | 71 | -0.1 | 0 | N/A |
| hsa-miR-4487 | 1646-1652 | 7mer-1A | -0.12 | 71 | -0.12 | 0 | N/A |
| hsa-miR-657 | 1652-1658 | 7mer-m8 | -0.02 | 43 | -0.02 | 0.021 | N/A |
| hsa-miR-761 | 1654-1660 | 7mer-m8 | -0.02 | 55 | -0.02 | 0.312 | N/A |
| hsa-miR-3619-5p | 1654-1660 | 7mer-m8 | -0.02 | 46 | -0.02 | 0.312 | N/A |
| hsa-miR-214-3p | 1654-1660 | 7mer-m8 | -0.02 | 43 | -0.02 | 0.312 | N/A |
| hsa-miR-107 | 1656-1662 | 7mer-1A | -0.01 | 18 | -0.01 | 2.853 | N/A |
| hsa-miR-103a-3p | 1656-1662 | 7mer-1A | -0.01 | 18 | -0.01 | 2.853 | N/A |
| hsa-miR-1184 | 1657-1663 | 7mer-m8 | -0.06 | 75 | -0.06 | 0.075 | N/A |
| hsa-miR-4267 | 1662-1668 | 7mer-1A | -0.01 | 42 | -0.01 | 0 | N/A |
| hsa-miR-766-3p | 1663-1669 | 7mer-m8 | -0.11 | 82 | -0.11 | 0.021 | N/A |
| hsa-miR-4691-5p | 1665-1672 | 8mer | -0.03 | 50 | -0.03 | 0 | N/A |
| hsa-miR-6792-3p | 1665-1672 | 8mer | -0.03 | 33 | -0.03 | 0 | N/A |
| hsa-miR-6749-3p | 1666-1672 | 7mer-1A | -0.01 | 26 | -0.01 | 0 | N/A |
| hsa-miR-516b-3p | 1668-1674 | 7mer-m8 | -0.02 | 26 | -0.02 | 0.021 | N/A |
| hsa-miR-516a-3p | 1668-1674 | 7mer-m8 | -0.02 | 26 | -0.02 | 0.021 | N/A |
| hsa-miR-7162-5p | 1668-1674 | 7mer-m8 | -0.02 | 24 | -0.02 | 0.021 | N/A |
| hsa-miR-6085 | 1672-1678 | 7mer-m8 | -0.12 | 73 | -0.12 | 0 | N/A |
| hsa-miR-6813-5p | 1672-1678 | 7mer-m8 | -0.09 | 69 | -0.09 | 0 | N/A |
| hsa-miR-6798-5p | 1674-1680 | 7mer-1A | -0.05 | 53 | -0.05 | 0.072 | N/A |
| hsa-miR-4518 | 1675-1681 | 7mer-m8 | -0.02 | 58 | -0.02 | 0.021 | N/A |
| hsa-miR-1266-5p | 1675-1681 | 7mer-m8 | -0.02 | 49 | -0.02 | 0.021 | N/A |
| hsa-miR-6865-3p | 1679-1685 | 7mer-m8 | -0.02 | 44 | -0.02 | 0 | N/A |
| hsa-miR-4715-3p | 1682-1688 | 7mer-m8 | -0.02 | 49 | -0.02 | 0 | N/A |
| hsa-miR-1227-3p | 1683-1689 | 7mer-m8 | -0.04 | 48 | -0.04 | 0.021 | N/A |
| hsa-miR-1825 | 1686-1693 | 8mer | -0.06 | 71 | -0.06 | 0.021 | N/A |
| hsa-miR-199a-5p | 1687-1693 | 7mer-1A | -0.01 | 31 | -0.01 | 0.419 | N/A |
| hsa-miR-199b-5p | 1687-1693 | 7mer-1A | -0.01 | 31 | -0.01 | 0.419 | N/A |
| hsa-miR-3925-3p | 1688-1694 | 7mer-m8 | -0.02 | 53 | -0.02 | 0 | N/A |
| hsa-miR-4281 | 1700-1706 | 7mer-1A | -0.1 | 80 | -0.1 | 0 | N/A |
| hsa-miR-133a-3p.1 | 1700-1706 | 7mer-m8 | -0.16 | 72 | -0.16 | 0.404 | N/A |
| hsa-miR-210-5p | 1707-1713 | 7mer-m8 | -0.07 | 54 | -0.07 | 0.072 | N/A |
| hsa-miR-874-5p | 1709-1715 | 7mer-1A | -0.18 | 76 | -0.18 | 0.072 | N/A |
| hsa-miR-663b | 1710-1717 | 8mer | -0.19 | 90 | -0.19 | 0.021 | N/A |
| hsa-miR-7108-5p | 1711-1717 | 7mer-m8 | -0.07 | 74 | -0.07 | 0 | N/A |
| hsa-miR-7114-5p | 1713-1719 | 7mer-1A | -0.07 | 65 | -0.07 | 0 | N/A |
| hsa-miR-134-3p | 1713-1719 | 7mer-1A | -0.06 | 61 | -0.06 | 0.072 | N/A |
| hsa-miR-593-3p | 1716-1723 | 8mer | -0.09 | 72 | -0.09 | 0.075 | N/A |
| hsa-miR-6818-3p | 1717-1723 | 7mer-m8 | -0.02 | 38 | -0.02 | 0 | N/A |
| hsa-miR-6895-3p | 1717-1723 | 7mer-1A | -0.01 | 32 | -0.01 | 0 | N/A |
| hsa-miR-626 | 1719-1726 | 8mer | -0.1 | 61 | -0.1 | 0.021 | N/A |
| hsa-miR-6876-3p | 1719-1726 | 8mer | -0.1 | 61 | -0.1 | 0.021 | N/A |
| hsa-miR-455-3p.2 | 1739-1746 | 8mer | -0.05 | 79 | -0.05 | 1.501 | N/A |
| hsa-miR-6516-5p | 1740-1747 | 8mer | -0.03 | 60 | -0.03 | 0.273 | N/A |
| hsa-miR-4748 | 1744-1750 | 7mer-m8 | -0.02 | 57 | -0.02 | 0 | N/A |
| hsa-miR-4464 | 1744-1750 | 7mer-m8 | -0.02 | 42 | -0.02 | 0 | N/A |
| hsa-miR-5006-5p | 1748-1754 | 7mer-m8 | -0.13 | 75 | -0.13 | 0 | N/A |
| hsa-miR-4755-3p | 1748-1754 | 7mer-1A | -0.05 | 52 | -0.05 | 0 | N/A |
| hsa-miR-4740-3p | 1757-1764 | 8mer | -0.16 | 86 | -0.16 | 0 | N/A |
| hsa-miR-4281 | 1760-1767 | 8mer | -0.31 | 98 | -0.31 | 0 | N/A |
| hsa-miR-133a-3p.1 | 1761-1767 | 7mer-m8 | -0.16 | 73 | -0.16 | 1.1 | N/A |
| hsa-miR-7152-3p | 1762-1768 | 7mer-m8 | -0.07 | 69 | -0.07 | 0 | N/A |
| hsa-miR-6131 | 1763-1770 | 8mer | -0.03 | 58 | -0.03 | 0.021 | N/A |
| hsa-miR-133a-5p | 1764-1770 | 7mer-1A | -0.09 | 65 | -0.09 | 0.072 | N/A |
| hsa-miR-138-5p | 1764-1770 | 7mer-1A | -0.08 | 58 | -0.08 | 0.769 | N/A |
| hsa-miR-3692-5p | 1765-1771 | 7mer-m8 | -0.05 | 63 | -0.05 | 0 | N/A |
| hsa-miR-6762-3p | 1767-1773 | 7mer-m8 | -0.02 | 26 | -0.02 | 0 | N/A |
| hsa-miR-6880-3p | 1774-1780 | 7mer-m8 | -0.15 | 63 | -0.15 | 0 | N/A |
| hsa-miR-566 | 1776-1782 | 7mer-m8 | -0.14 | 72 | -0.14 | 0.021 | N/A |
| hsa-miR-3197 | 1778-1784 | 7mer-m8 | -0.27 | 94 | -0.27 | 0 | N/A |
| hsa-miR-4257 | 1781-1787 | 7mer-1A | -0.06 | 76 | -0.06 | 0 | N/A |
| hsa-miR-6847-5p | 1781-1787 | 7mer-1A | -0.04 | 50 | -0.04 | 0 | N/A |
| hsa-miR-6801-5p | 1783-1789 | 7mer-m8 | -0.06 | 56 | -0.06 | 0 | N/A |
| hsa-miR-6804-5p | 1798-1804 | 7mer-1A | -0.09 | 51 | -0.09 | 0 | N/A |
| hsa-miR-7162-3p | 1800-1806 | 7mer-1A | -0.01 | 41 | -0.01 | 0 | N/A |
| hsa-miR-4649-3p | 1800-1806 | 7mer-1A | -0.01 | 41 | -0.01 | 0 | N/A |
| hsa-miR-4711-3p | 1804-1810 | 7mer-1A | -0.01 | 26 | -0.01 | 0 | N/A |
| hsa-miR-6867-5p | 1806-1812 | 7mer-1A | -0.12 | 55 | -0.12 | 0 | N/A |
| hsa-miR-26b-3p | 1812-1818 | 7mer-m8 | -0.02 | 44 | -0.02 | 0.468 | N/A |
| hsa-miR-1208 | 1814-1821 | 8mer | -0.03 | 60 | -0.03 | 0.021 | N/A |
| hsa-miR-6760-3p | 1815-1821 | 7mer-1A | -0.01 | 39 | -0.01 | 0 | N/A |
| hsa-miR-5681a | 1824-1830 | 7mer-m8 | -0.02 | 40 | -0.02 | 0 | N/A |
| hsa-miR-3978 | 1826-1832 | 7mer-m8 | -0.02 | 64 | -0.02 | 0 | N/A |
| hsa-miR-6089 | 1840-1846 | 7mer-m8 | -0.07 | 77 | -0.07 | 0 | N/A |
| hsa-miR-4695-5p | 1842-1849 | 8mer | -0.19 | 89 | -0.19 | 0 | N/A |
| hsa-miR-4266 | 1843-1850 | 8mer | -0.16 | 75 | -0.16 | 0 | N/A |
| hsa-miR-4779 | 1843-1849 | 7mer-1A | -0.05 | 66 | -0.05 | 0 | N/A |
| hsa-miR-651-5p | 1845-1851 | 7mer-1A | -0.01 | 25 | -0.01 | 0.021 | N/A |
| hsa-miR-4459 | 1852-1858 | 7mer-m8 | -0.2 | 94 | -0.2 | 0 | N/A |
| hsa-miR-4722-5p | 1853-1859 | 7mer-m8 | -0.08 | 71 | -0.08 | 0 | N/A |
| hsa-miR-6165 | 1854-1860 | 7mer-m8 | -0.11 | 79 | -0.11 | 0 | N/A |
| hsa-miR-214-3p | 1855-1862 | 8mer | -0.22 | 95 | -0.22 | 0.075 | N/A |
| hsa-miR-3619-5p | 1855-1862 | 8mer | -0.2 | 93 | -0.2 | 0.075 | N/A |
| hsa-miR-761 | 1855-1862 | 8mer | -0.12 | 91 | -0.12 | 0.075 | N/A |
| hsa-miR-4291 | 1856-1863 | 8mer | -0.19 | 90 | -0.19 | 0 | N/A |
| hsa-miR-922 | 1856-1862 | 7mer-1A | -0.1 | 89 | -0.1 | 0.075 | N/A |
| hsa-miR-6758-3p | 1861-1867 | 7mer-m8 | -0.02 | 55 | -0.02 | 0 | N/A |
| hsa-miR-6891-3p | 1863-1869 | 7mer-1A | -0.01 | 33 | -0.01 | 0 | N/A |
| hsa-miR-6072 | 1863-1869 | 7mer-1A | -0.01 | 30 | -0.01 | 0 | N/A |
| hsa-miR-4257 | 1869-1875 | 7mer-m8 | -0.02 | 48 | -0.02 | 0 | N/A |
| hsa-miR-2467-3p | 1870-1876 | 7mer-m8 | -0.07 | 62 | -0.07 | 0.668 | N/A |
| hsa-miR-4468 | 1872-1879 | 8mer | -0.03 | 30 | -0.03 | 0 | N/A |
| hsa-miR-4663 | 1875-1881 | 7mer-m8 | -0.1 | 83 | -0.1 | 0 | N/A |
| hsa-miR-3929 | 1877-1883 | 7mer-m8 | -0.02 | 54 | -0.02 | 0 | N/A |
| hsa-miR-4478 | 1877-1883 | 7mer-m8 | -0.02 | 54 | -0.02 | 0 | N/A |
| hsa-miR-4419b | 1877-1883 | 7mer-m8 | -0.02 | 54 | -0.02 | 0 | N/A |
| hsa-miR-6884-5p | 1878-1884 | 7mer-m8 | -0.08 | 56 | -0.08 | 0.360 | N/A |
| hsa-miR-485-5p | 1878-1884 | 7mer-m8 | -0.05 | 44 | -0.05 | 0.360 | N/A |
| hsa-miR-922 | 1889-1895 | 7mer-m8 | -0.02 | 52 | -0.02 | 0.075 | N/A |
| hsa-miR-497-5p | 1891-1897 | 7mer-m8 | -0.02 | 28 | -0.02 | 0.201 | N/A |
| hsa-miR-424-5p | 1891-1897 | 7mer-m8 | -0.02 | 28 | -0.02 | 0.201 | N/A |
| hsa-miR-195-5p | 1891-1897 | 7mer-m8 | -0.02 | 27 | -0.02 | 0.201 | N/A |
| hsa-miR-16-5p | 1891-1897 | 7mer-m8 | -0.02 | 27 | -0.02 | 0.201 | N/A |
| hsa-miR-15b-5p | 1891-1897 | 7mer-m8 | -0.02 | 25 | -0.02 | 0.201 | N/A |
| hsa-miR-15a-5p | 1891-1897 | 7mer-m8 | -0.02 | 25 | -0.02 | 0.201 | N/A |
| hsa-miR-6838-5p | 1891-1897 | 7mer-m8 | -0.02 | 23 | -0.02 | 0.201 | N/A |
| hsa-miR-4671-5p | 1896-1902 | 7mer-1A | -0.15 | 67 | -0.15 | 0 | N/A |
| hsa-miR-1203 | 1911-1917 | 7mer-m8 | -0.06 | 41 | -0.06 | 0 | N/A |
| hsa-miR-4537 | 1915-1921 | 7mer-1A | -0.07 | 26 | -0.07 | 0 | N/A |
| hsa-miR-4690-3p | 1923-1929 | 7mer-m8 | -0.02 | 38 | -0.02 | 0 | N/A |
| hsa-miR-5685 | 1923-1929 | 7mer-m8 | -0.02 | 26 | -0.02 | 0 | N/A |
| hsa-miR-3186-5p | 1931-1937 | 7mer-m8 | -0.08 | 72 | -0.08 | 0 | N/A |
| hsa-miR-6825-5p | 1935-1941 | 7mer-m8 | -0.02 | 16 | -0.02 | 0 | N/A |
| hsa-miR-6777-5p | 1936-1943 | 8mer | -0.29 | 80 | -0.29 | 0 | N/A |
| hsa-miR-6889-5p | 1936-1943 | 8mer | -0.24 | 69 | -0.24 | 0 | N/A |
| hsa-miR-542-5p | 1937-1943 | 7mer-1A | -0.12 | 65 | -0.12 | 0.072 | N/A |
| hsa-miR-4286 | 1943-1949 | 7mer-m8 | -0.02 | 36 | -0.02 | 0 | N/A |
| hsa-miR-4758-3p | 1944-1951 | 8mer | -0.1 | 75 | -0.1 | 0 | N/A |
| hsa-miR-5699-5p | 1945-1951 | 7mer-1A | -0.06 | 49 | -0.06 | 0 | N/A |
| hsa-miR-3189-5p | 1945-1951 | 7mer-1A | -0.01 | 16 | -0.01 | 0 | N/A |
| hsa-miR-1913 | 1946-1952 | 7mer-m8 | -0.07 | 69 | -0.07 | 0.021 | N/A |
| hsa-miR-324-3p | 1946-1952 | 7mer-m8 | -0.05 | 61 | -0.05 | 0.021 | N/A |
| hsa-miR-4763-5p | 1948-1955 | 8mer | -0.21 | 79 | -0.21 | 0 | N/A |
| hsa-miR-6894-3p | 1949-1955 | 7mer-m8 | -0.08 | 79 | -0.08 | 0.072 | N/A |
| hsa-miR-4999-3p | 1956-1962 | 7mer-1A | -0.01 | 24 | -0.01 | 0 | N/A |
| hsa-miR-668-3p | 1958-1964 | 7mer-1A | -0.01 | 28 | -0.01 | 3.324 | N/A |
| hsa-miR-597-5p | 1959-1966 | 8mer | -0.16 | 84 | -0.16 | 0.448 | N/A |
| hsa-miR-592 | 1960-1967 | 8mer | -0.03 | 34 | -0.03 | 0.448 | N/A |
| hsa-miR-581 | 1962-1968 | 7mer-m8 | -0.06 | 64 | -0.06 | 0.448 | N/A |
| hsa-miR-4774-5p | 1971-1977 | 7mer-m8 | -0.06 | 54 | -0.06 | 0 | N/A |
| hsa-miR-133a-5p | 1972-1979 | 8mer | -0.25 | 92 | -0.25 | 0.938 | N/A |
| hsa-miR-6131 | 1973-1979 | 7mer-1A | -0.01 | 41 | -0.01 | 0.021 | N/A |
| hsa-miR-2115-5p | 1987-1994 | 8mer | -0.32 | 96 | -0.32 | 0 | N/A |
| hsa-miR-7162-5p | 1988-1994 | 7mer-1A | -0.11 | 73 | -0.11 | 0.021 | N/A |
| hsa-miR-516b-3p | 1988-1994 | 7mer-1A | -0.06 | 57 | -0.06 | 0.021 | N/A |
| hsa-miR-516a-3p | 1988-1994 | 7mer-1A | -0.06 | 57 | -0.06 | 0.021 | N/A |
| hsa-miR-4689 | 1999-2005 | 7mer-m8 | -0.15 | 84 | -0.15 | 0 | N/A |
| hsa-miR-6858-5p | 1999-2005 | 7mer-m8 | -0.09 | 81 | -0.09 | 0 | N/A |
| hsa-miR-4784 | 1999-2005 | 7mer-1A | -0.13 | 77 | -0.13 | 0 | N/A |
| hsa-miR-3150b-3p | 1999-2005 | 7mer-1A | -0.1 | 72 | -0.1 | 0 | N/A |
| hsa-miR-7162-3p | 2001-2007 | 7mer-1A | -0.07 | 84 | -0.07 | 0 | N/A |
| hsa-miR-4649-3p | 2001-2007 | 7mer-1A | -0.04 | 68 | -0.04 | 0 | N/A |
| hsa-miR-4653-5p | 2002-2009 | 8mer | -0.2 | 94 | -0.2 | 0 | N/A |
| hsa-miR-3921 | 2002-2009 | 8mer | -0.2 | 94 | -0.2 | 0 | N/A |
| hsa-miR-146a-3p | 2003-2009 | 7mer-1A | -0.09 | 74 | -0.09 | 0 | N/A |
| hsa-miR-301a-5p | 2003-2009 | 7mer-1A | -0.01 | 48 | -0.01 | 0.505 | N/A |
| hsa-miR-301b-5p | 2003-2009 | 7mer-1A | -0.01 | 48 | -0.01 | 0.505 | N/A |
| hsa-miR-3191-5p | 2004-2010 | 7mer-1A | -0.06 | 67 | -0.06 | 0 | N/A |
| hsa-miR-8070 | 2012-2019 | 8mer | -0.2 | 93 | -0.2 | 0 | N/A |
| hsa-miR-758-3p | 2014-2020 | 7mer-1A | -0.02 | 51 | -0.02 | 1.883 | N/A |
| hsa-miR-1251-3p | 2017-2023 | 7mer-1A | -0.03 | 57 | -0.03 | 0.445 | N/A |
| hsa-miR-6885-3p | 2019-2026 | 8mer | -0.06 | 78 | -0.06 | 0 | N/A |
| hsa-miR-129-5p | 2021-2027 | 7mer-m8 | -0.04 | 91 | -0.04 | 1.107 | N/A |
| hsa-miR-153-5p | 2023-2030 | 8mer | -0.03 | 71 | -0.03 | 0.938 | N/A |
| hsa-miR-1250-3p | 2024-2030 | 7mer-1A | -0.01 | 46 | -0.01 | 0 | N/A |
| hsa-miR-5696 | 2025-2031 | 7mer-1A | -0.01 | 46 | -0.01 | 0 | N/A |
| hsa-miR-664b-3p | 2025-2031 | 7mer-1A | -0.01 | 42 | -0.01 | 0.075 | N/A |
| hsa-miR-579-3p | 2025-2031 | 7mer-1A | -0.01 | 41 | -0.01 | 0.075 | N/A |
| hsa-miR-6853-3p | 2026-2033 | 8mer | -0.11 | 76 | -0.11 | 0 | N/A |
| hsa-miR-1206 | 2027-2033 | 7mer-1A | -0.08 | 67 | -0.08 | 0.021 | N/A |
| hsa-miR-124-5p | 2028-2034 | 7mer-m8 | -0.2 | 83 | -0.2 | 0 | N/A |
| hsa-miR-4255 | 2029-2035 | 7mer-m8 | -0.15 | 89 | -0.15 | 0 | N/A |
| hsa-miR-199a-5p | 2031-2037 | 7mer-m8 | -0.17 | 86 | -0.17 | 0.201 | N/A |
| hsa-miR-199b-5p | 2031-2037 | 7mer-m8 | -0.17 | 86 | -0.17 | 0.201 | N/A |
| hsa-miR-587 | 2038-2044 | 7mer-m8 | -0.02 | 39 | -0.02 | 0.021 | N/A |
| hsa-miR-6875-3p | 2041-2048 | 8mer | -0.11 | 85 | -0.11 | 0 | N/A |
| hsa-miR-4659a-3p | 2042-2048 | 7mer-1A | -0.01 | 24 | -0.01 | 0 | N/A |
| hsa-miR-4659b-3p | 2042-2048 | 7mer-1A | -0.01 | 24 | -0.01 | 0 | N/A |
| hsa-miR-1178-3p | 2049-2055 | 7mer-m8 | -0.28 | 96 | -0.28 | 0.021 | N/A |
| hsa-miR-203a-3p.1 | 2054-2060 | 7mer-1A | -0.01 | 33 | -0.01 | 1.899 | N/A |
| hsa-miR-521 | 2064-2070 | 7mer-m8 | -0.21 | 84 | -0.21 | 0.075 | N/A |
| hsa-miR-1286 | 2069-2076 | 8mer | -0.29 | 97 | -0.29 | 0.021 | N/A |
| hsa-miR-873-5p.2 | 2070-2076 | 7mer-1A | -0.05 | 45 | -0.05 | 2.379 | N/A |
| hsa-miR-4722-5p | 2070-2076 | 7mer-1A | -0.01 | 26 | -0.01 | 0 | N/A |
| hsa-miR-1205 | 2071-2077 | 7mer-m8 | -0.16 | 92 | -0.16 | 0.021 | N/A |
| hsa-miR-4713-3p | 2077-2084 | 8mer | -0.26 | 86 | -0.26 | 0 | N/A |
| hsa-miR-450b-3p | 2078-2084 | 7mer-m8 | -0.14 | 89 | -0.14 | 0.075 | N/A |
| hsa-miR-769-3p | 2078-2084 | 7mer-m8 | -0.09 | 84 | -0.09 | 0.075 | N/A |
| hsa-miR-638 | 2078-2084 | 7mer-1A | -0.11 | 66 | -0.11 | 0.072 | N/A |
| hsa-miR-4291 | 2090-2096 | 7mer-m8 | -0.04 | 51 | -0.04 | 0.409 | N/A |
| hsa-miR-761 | 2090-2096 | 7mer-1A | -0.01 | 45 | -0.01 | 1.167 | N/A |
| hsa-miR-214-3p | 2090-2096 | 7mer-1A | -0.02 | 43 | -0.02 | 1.167 | N/A |
| hsa-miR-922 | 2090-2096 | 7mer-1A | -0.01 | 40 | -0.01 | 0.075 | N/A |
| hsa-miR-3619-5p | 2090-2096 | 7mer-1A | -0.01 | 36 | -0.01 | 1.167 | N/A |
| hsa-miR-3617-3p | 2091-2097 | 7mer-m8 | -0.02 | 17 | -0.02 | 1.021 | N/A |
| hsa-miR-4769-3p | 2096-2103 | 8mer | -0.03 | 56 | -0.03 | 0 | N/A |
| hsa-miR-6817-5p | 2096-2103 | 8mer | -0.03 | 54 | -0.03 | 0 | N/A |
| hsa-miR-4727-5p | 2097-2103 | 7mer-m8 | -0.02 | 21 | -0.02 | 0 | N/A |
| hsa-miR-6876-3p | 2108-2114 | 7mer-1A | -0.04 | 37 | -0.04 | 0.021 | N/A |
| hsa-miR-626 | 2108-2114 | 7mer-1A | -0.04 | 36 | -0.04 | 0.021 | N/A |
| hsa-miR-598-3p | 2123-2130 | 8mer | -0.25 | 86 | -0.25 | 0.075 | N/A |
| hsa-miR-133a-5p | 2129-2135 | 7mer-m8 | -0.02 | 15 | -0.02 | 0.452 | N/A |
| hsa-miR-1207-3p | 2131-2137 | 7mer-m8 | -0.09 | 77 | -0.09 | 0.411 | N/A |
| hsa-miR-3714 | 2135-2141 | 7mer-m8 | -0.02 | 49 | -0.02 | 0 | N/A |
| hsa-miR-605-3p | 2136-2142 | 7mer-m8 | -0.02 | 15 | -0.02 | 0 | N/A |
| hsa-miR-4747-5p | 2138-2144 | 7mer-m8 | -0.06 | 56 | -0.06 | 0 | N/A |
| hsa-miR-5196-5p | 2138-2144 | 7mer-m8 | -0.03 | 41 | -0.03 | 0 | N/A |
| hsa-miR-425-3p | 2140-2147 | 8mer | -0.32 | 91 | -0.32 | 0 | N/A |
| hsa-miR-6771-5p | 2141-2147 | 7mer-1A | -0.19 | 64 | -0.19 | 0 | N/A |
| hsa-miR-513a-3p | 2145-2152 | 8mer | -0.03 | 89 | -0.03 | 0 | N/A |
| hsa-miR-3606-3p | 2145-2152 | 8mer | -0.03 | 89 | -0.03 | 0 | N/A |
| hsa-miR-513c-3p | 2145-2152 | 8mer | -0.03 | 89 | -0.03 | 0 | N/A |
| hsa-miR-4799-5p | 2148-2155 | 8mer | -0.03 | 72 | -0.03 | 0 | N/A |
| hsa-miR-548ay-3p | 2157-2163 | 7mer-m8 | -0.02 | 67 | -0.02 | 0 | N/A |
| hsa-miR-548at-3p | 2157-2163 | 7mer-m8 | -0.02 | 65 | -0.02 | 0 | N/A |
| hsa-miR-548o-3p | 2159-2165 | 7mer-1A | -0.01 | 38 | -0.01 | 0.075 | N/A |
| hsa-miR-1323 | 2159-2165 | 7mer-1A | -0.01 | 36 | -0.01 | 0.075 | N/A |
| hsa-miR-205-5p | 2163-2169 | 7mer-1A | -0.01 | 28 | -0.01 | 3.441 | N/A |
| hsa-miR-5193 | 2166-2172 | 7mer-1A | -0.01 | 34 | -0.01 | 0 | N/A |
| hsa-miR-660-3p | 2166-2172 | 7mer-1A | -0.01 | 24 | -0.01 | 0.072 | N/A |
| hsa-miR-6852-3p | 2168-2174 | 7mer-1A | -0.03 | 31 | -0.03 | 0 | N/A |
| hsa-miR-3909 | 2168-2174 | 7mer-1A | -0.02 | 20 | -0.02 | 0 | N/A |
| hsa-miR-6817-3p | 2173-2180 | 8mer | -0.16 | 90 | -0.16 | 0 | N/A |
| hsa-miR-6873-3p | 2174-2181 | 8mer | -0.03 | 46 | -0.03 | 0 | N/A |
| hsa-miR-7110-3p | 2174-2180 | 7mer-1A | -0.01 | 30 | -0.01 | 0 | N/A |
| hsa-miR-6833-3p | 2175-2181 | 7mer-m8 | -0.02 | 61 | -0.02 | 0 | N/A |
| hsa-miR-4768-5p | 2175-2181 | 7mer-m8 | -0.02 | 59 | -0.02 | 0 | N/A |
| hsa-miR-942-5p | 2176-2182 | 7mer-m8 | -0.02 | 38 | -0.02 | 0.021 | N/A |
| hsa-miR-28-5p | 2187-2194 | 8mer | -0.2 | 90 | -0.2 | 0.031 | N/A |
| hsa-miR-708-5p | 2187-2194 | 8mer | -0.2 | 90 | -0.2 | 0.031 | N/A |
| hsa-miR-3139 | 2187-2194 | 8mer | -0.17 | 87 | -0.17 | 0.031 | N/A |
| hsa-miR-8065 | 2190-2196 | 7mer-1A | -0.03 | 13 | -0.03 | 0 | N/A |
| hsa-miR-3137 | 2192-2198 | 7mer-1A | -0.01 | 39 | -0.01 | 0 | N/A |
| hsa-miR-20b-3p | 2192-2198 | 7mer-1A | -0.01 | 20 | -0.01 | 0 | N/A |
| hsa-miR-3686 | 2193-2199 | 7mer-1A | -0.02 | 31 | -0.02 | 0 | N/A |
| hsa-miR-4743-3p | 2194-2200 | 7mer-1A | -0.01 | 39 | -0.01 | 0 | N/A |
| hsa-miR-4652-3p | 2194-2200 | 7mer-1A | -0.01 | 36 | -0.01 | 0 | N/A |
| hsa-miR-4308 | 2196-2207 | non-canonical | N/A | N/A | N/A | 0 | N/A |
| hsa-miR-4308 | 2196-2207 | non-canonical | N/A | N/A | N/A | 0 | N/A |
| hsa-miR-4653-3p | 2199-2205 | 7mer-1A | -0.08 | 69 | -0.08 | 0 | N/A |
| hsa-miR-3135b | 2201-2207 | 7mer-m8 | -0.03 | 48 | -0.03 | 0 | N/A |
| hsa-miR-3652 | 2202-2208 | 7mer-m8 | -0.08 | 72 | -0.08 | 0.310 | N/A |
| hsa-miR-4430 | 2202-2208 | 7mer-m8 | -0.08 | 69 | -0.08 | 0.310 | N/A |
| hsa-miR-762 | 2203-2210 | 8mer | -0.34 | 97 | -0.34 | 0.075 | N/A |
| hsa-miR-5001-5p | 2203-2210 | 8mer | -0.43 | 97 | -0.43 | 0.075 | N/A |
| hsa-miR-4498 | 2203-2210 | 8mer | -0.39 | 97 | -0.39 | 0.075 | N/A |
| hsa-miR-4492 | 2203-2210 | 8mer | -0.31 | 96 | -0.31 | 0.075 | N/A |
| hsa-miR-4741 | 2204-2210 | 7mer-1A | -0.11 | 79 | -0.11 | 0 | N/A |
| hsa-miR-4675 | 2204-2210 | 7mer-1A | -0.06 | 76 | -0.06 | 0 | N/A |
| hsa-miR-4656 | 2204-2210 | 7mer-1A | -0.13 | 76 | -0.13 | 0 | N/A |
| hsa-miR-6829-5p | 2204-2210 | 7mer-1A | -0.08 | 71 | -0.08 | 0 | N/A |
| hsa-miR-1587 | 2204-2210 | 7mer-m8 | -0.11 | 69 | -0.11 | 0 | N/A |
| hsa-miR-3620-5p | 2204-2210 | 7mer-m8 | -0.04 | 61 | -0.04 | 0 | N/A |
| hsa-miR-378g | 2205-2211 | 7mer-m8 | -0.1 | 81 | -0.1 | 0 | N/A |
| hsa-miR-939-3p | 2206-2212 | 7mer-m8 | -0.15 | 85 | -0.15 | 0 | N/A |
| hsa-miR-7851-3p | 2207-2213 | 7mer-m8 | -0.16 | 76 | -0.16 | 0 | N/A |
| hsa-miR-490-3p | 2208-2214 | 7mer-m8 | -0.02 | 37 | -0.02 | 0.828 | N/A |
| hsa-miR-3605-3p | 2215-2222 | 8mer | -0.36 | 94 | -0.36 | 0 | N/A |
| hsa-miR-4522 | 2220-2226 | 7mer-1A | -0.01 | 17 | -0.01 | 0 | N/A |
| hsa-miR-345-5p | 2221-2228 | 8mer | -0.03 | 70 | -0.03 | 0.075 | N/A |
| hsa-miR-4256 | 2223-2229 | 7mer-1A | -0.06 | 60 | -0.06 | 0 | N/A |
| hsa-miR-6838-3p | 2225-2236 | non-canonical | N/A | N/A | N/A | 0 | N/A |
| hsa-miR-6838-3p | 2225-2236 | non-canonical | N/A | N/A | N/A | 0 | N/A |
| hsa-miR-103a-2-5p | 2226-2232 | 7mer-1A | -0.01 | 21 | -0.01 | 0.072 | N/A |
| hsa-miR-6893-3p | 2229-2235 | 7mer-1A | -0.19 | 89 | -0.19 | 1.909 | N/A |
| hsa-miR-370-3p | 2229-2235 | 7mer-1A | -0.13 | 88 | -0.13 | 1.909 | N/A |
| hsa-miR-1976 | 2230-2236 | 7mer-m8 | -0.03 | 66 | -0.03 | 0 | N/A |
| hsa-miR-6736-3p | 2233-2239 | 7mer-m8 | -0.07 | 72 | -0.07 | 0 | N/A |
| hsa-miR-4660 | 2234-2240 | 7mer-m8 | -0.02 | 39 | -0.02 | 0.021 | N/A |
| hsa-miR-6790-3p | 2242-2248 | 7mer-m8 | -0.02 | 32 | -0.02 | 0 | N/A |
| hsa-miR-6861-3p | 2243-2250 | 8mer | -0.08 | 63 | -0.08 | 0 | N/A |
| hsa-miR-659-5p | 2244-2250 | 7mer-1A | -0.08 | 58 | -0.08 | 0 | N/A |
| hsa-miR-4535 | 2244-2250 | 7mer-m8 | -0.02 | 28 | -0.02 | 0 | N/A |
| hsa-miR-492 | 2244-2250 | 7mer-1A | -0.03 | 25 | -0.03 | 0.075 | N/A |
| hsa-miR-4800-5p | 2245-2251 | 7mer-m8 | -0.12 | 68 | -0.12 | 0 | N/A |
| hsa-miR-8071 | 2246-2253 | 8mer | -0.1 | 78 | -0.1 | 0 | N/A |
| hsa-miR-6880-5p | 2247-2253 | 7mer-1A | -0.05 | 43 | -0.05 | 0 | N/A |
| hsa-miR-3165 | 2247-2253 | 7mer-1A | -0.04 | 41 | -0.04 | 0.021 | N/A |
| hsa-miR-4456 | 2248-2254 | 7mer-m8 | -0.08 | 66 | -0.08 | 0 | N/A |
| hsa-miR-181a-2-3p | 2252-2258 | 7mer-1A | -0.03 | 53 | -0.03 | 0 | N/A |
| hsa-miR-1273g-3p | 2252-2258 | 7mer-1A | -0.01 | 32 | -0.01 | 0 | N/A |
| hsa-miR-6509-3p | 2252-2258 | 7mer-m8 | -0.02 | 29 | -0.02 | 0 | N/A |
| hsa-miR-324-5p | 2256-2262 | 7mer-1A | -0.02 | 27 | -0.02 | 0.994 | N/A |
| hsa-miR-20a-3p | 2258-2264 | 7mer-1A | -0.01 | 22 | -0.01 | 0.072 | N/A |
| hsa-miR-4632-3p | 2266-2272 | 7mer-m8 | -0.16 | 59 | -0.16 | 0 | N/A |
| hsa-miR-6721-5p | 2272-2278 | 7mer-m8 | -0.02 | 29 | -0.02 | 0 | N/A |
| hsa-miR-7112-5p | 2273-2279 | 7mer-m8 | -0.07 | 31 | -0.07 | 0 | N/A |
| hsa-miR-4767 | 2275-2281 | 7mer-1A | -0.19 | 67 | -0.19 | 0 | N/A |
| hsa-miR-4734 | 2276-2282 | 7mer-m8 | -0.02 | 21 | -0.02 | 0 | N/A |
| hsa-miR-604 | 2278-2285 | 8mer | -0.08 | 67 | -0.08 | 0.021 | N/A |
| hsa-miR-647 | 2279-2285 | 7mer-m8 | -0.02 | 58 | -0.02 | 0 | N/A |
| hsa-miR-6762-3p | 2279-2285 | 7mer-1A | -0.04 | 46 | -0.04 | 0 | N/A |
| hsa-miR-889-5p | 2280-2286 | 7mer-m8 | -0.02 | 30 | -0.02 | 0.072 | N/A |
| hsa-miR-6817-5p | 2292-2298 | 7mer-1A | -0.02 | 47 | -0.02 | 0 | N/A |
| hsa-miR-4727-5p | 2292-2299 | 8mer | -0.03 | 34 | -0.03 | 0 | N/A |
| hsa-miR-4769-3p | 2292-2298 | 7mer-1A | -0.01 | 31 | -0.01 | 0 | N/A |
| hsa-miR-6738-3p | 2293-2299 | 7mer-m8 | -0.07 | 68 | -0.07 | 0 | N/A |
| hsa-miR-5001-3p | 2293-2299 | 7mer-1A | -0.01 | 18 | -0.01 | 0 | N/A |
| hsa-miR-545-5p | 2304-2310 | 7mer-m8 | -0.02 | 28 | -0.02 | 0.430 | N/A |
| hsa-miR-513a-5p | 2308-2314 | 7mer-1A | -0.04 | 64 | -0.04 | 0.075 | N/A |
| hsa-miR-5096 | 2310-2316 | 7mer-1A | -0.01 | 44 | -0.01 | 0 | N/A |
| hsa-miR-502-5p | 2317-2323 | 7mer-1A | -0.05 | 67 | -0.05 | 0.075 | N/A |
| hsa-miR-6782-3p | 2324-2330 | 7mer-1A | -0.05 | 69 | -0.05 | 0 | N/A |
| hsa-miR-4724-3p | 2325-2331 | 7mer-1A | -0.01 | 40 | -0.01 | 0 | N/A |
| hsa-miR-556-3p | 2327-2333 | 7mer-m8 | -0.12 | 87 | -0.12 | 0.021 | N/A |
| hsa-miR-5579-5p | 2340-2346 | 7mer-1A | -0.03 | 49 | -0.03 | 0 | N/A |
| hsa-miR-5007-3p | 2343-2349 | 7mer-m8 | -0.02 | 39 | -0.02 | 0 | N/A |
| hsa-miR-6072 | 2346-2352 | 7mer-1A | -0.04 | 60 | -0.04 | 0 | N/A |
| hsa-miR-6891-3p | 2346-2352 | 7mer-1A | -0.01 | 33 | -0.01 | 0 | N/A |
| hsa-miR-670-3p | 2347-2353 | 7mer-m8 | -0.04 | 54 | -0.04 | 1.762 | N/A |
| hsa-miR-141-5p | 2350-2357 | 8mer | -0.17 | 88 | -0.17 | 0 | N/A |
| hsa-miR-1252-5p | 2358-2364 | 7mer-m8 | -0.02 | 20 | -0.02 | 0.021 | N/A |
| hsa-miR-6770-5p | 2360-2366 | 7mer-m8 | -0.02 | 43 | -0.02 | 0 | N/A |
| hsa-miR-3919 | 2362-2368 | 7mer-m8 | -0.02 | 44 | -0.02 | 0 | N/A |
| hsa-miR-5588-3p | 2367-2373 | 7mer-m8 | -0.02 | 12 | -0.02 | 0 | N/A |
| hsa-miR-8077 | 2371-2377 | 7mer-m8 | -0.02 | 64 | -0.02 | 0 | N/A |
| hsa-miR-6825-3p | 2373-2379 | 7mer-1A | -0.04 | 36 | -0.04 | 0 | N/A |
| hsa-miR-639 | 2374-2380 | 7mer-1A | -0.14 | 64 | -0.14 | 0 | N/A |
| hsa-miR-4669 | 2385-2391 | 7mer-m8 | -0.11 | 75 | -0.11 | 0 | N/A |
| hsa-miR-6844 | 2392-2398 | 7mer-1A | -0.01 | 30 | -0.01 | 0 | N/A |
| hsa-miR-4778-3p | 2394-2400 | 7mer-1A | -0.01 | 32 | -0.01 | 0 | N/A |
| hsa-miR-6740-3p | 2395-2401 | 7mer-m8 | -0.09 | 66 | -0.09 | 0 | N/A |
| hsa-miR-1250-3p | 2402-2408 | 7mer-m8 | -0.02 | 67 | -0.02 | 0 | N/A |
| hsa-miR-4713-3p | 2408-2414 | 7mer-m8 | -0.21 | 80 | -0.21 | 0 | N/A |
| hsa-miR-4525 | 2410-2416 | 7mer-m8 | -0.08 | 67 | -0.08 | 0 | N/A |
| hsa-miR-5010-5p | 2410-2416 | 7mer-m8 | -0.1 | 58 | -0.1 | 0 | N/A |
| hsa-miR-6794-5p | 2411-2417 | 7mer-m8 | -0.16 | 72 | -0.16 | 0 | N/A |
| hsa-miR-4716-3p | 2411-2417 | 7mer-m8 | -0.17 | 72 | -0.17 | 0 | N/A |
| hsa-miR-561-5p | 2415-2421 | 7mer-1A | -0.02 | 21 | -0.02 | 0 | N/A |
| hsa-miR-8070 | 2420-2426 | 7mer-m8 | -0.02 | 37 | -0.02 | 0 | N/A |
| hsa-miR-4679 | 2421-2427 | 7mer-m8 | -0.02 | 43 | -0.02 | 0 | N/A |
| hsa-miR-1914-5p | 2423-2429 | 7mer-1A | -0.01 | 40 | -0.01 | 0 | N/A |
| hsa-miR-6828-3p | 2435-2441 | 7mer-m8 | -0.07 | 72 | -0.07 | 0 | N/A |
| hsa-miR-767-3p | 2435-2441 | 7mer-1A | -0.04 | 50 | -0.04 | 0.072 | N/A |
| hsa-miR-1909-3p | 2447-2453 | 7mer-m8 | -0.14 | 75 | -0.14 | 0 | N/A |
| hsa-miR-6722-3p | 2447-2453 | 7mer-m8 | -0.11 | 69 | -0.11 | 0 | N/A |
| hsa-miR-4763-3p | 2448-2454 | 7mer-m8 | -0.08 | 64 | -0.08 | 0 | N/A |
| hsa-miR-1207-5p | 2448-2454 | 7mer-m8 | -0.05 | 58 | -0.05 | 0 | N/A |
| hsa-miR-6721-5p | 2449-2455 | 7mer-m8 | -0.05 | 50 | -0.05 | 0 | N/A |
| hsa-miR-486-3p | 2450-2457 | 8mer | -0.15 | 60 | -0.15 | 0 | N/A |
| hsa-miR-3151-5p | 2452-2458 | 7mer-m8 | -0.07 | 71 | -0.07 | 0.021 | N/A |
| hsa-miR-92a-2-5p | 2453-2459 | 7mer-m8 | -0.08 | 66 | -0.08 | 0 | N/A |
| hsa-miR-6134 | 2455-2462 | 8mer | -0.03 | 34 | -0.03 | 0.021 | N/A |
| hsa-miR-7854-3p | 2456-2462 | 7mer-1A | -0.01 | 14 | -0.01 | 0 | N/A |
| hsa-miR-7162-3p | 2457-2463 | 7mer-m8 | -0.02 | 52 | -0.02 | 0 | N/A |
| hsa-miR-7160-5p | 2458-2464 | 7mer-m8 | -0.02 | 31 | -0.02 | 0 | N/A |
| hsa-miR-4656 | 2460-2466 | 7mer-m8 | -0.05 | 44 | -0.05 | 0 | N/A |
| hsa-miR-6762-3p | 2468-2474 | 7mer-1A | -0.1 | 68 | -0.1 | 0 | N/A |
| hsa-miR-604 | 2468-2474 | 7mer-1A | -0.06 | 59 | -0.06 | 0.021 | N/A |
| hsa-miR-647 | 2468-2474 | 7mer-m8 | -0.02 | 58 | -0.02 | 0 | N/A |
| hsa-miR-3064-5p | 2469-2475 | 7mer-m8 | -0.05 | 58 | -0.05 | 1.002 | 0.18 |
| hsa-miR-6504-5p | 2469-2475 | 7mer-m8 | -0.03 | 51 | -0.03 | 1.002 | 0.18 |
| hsa-miR-5580-5p | 2470-2476 | 7mer-m8 | -0.02 | 20 | -0.02 | 0.072 | N/A |
| hsa-miR-7978 | 2482-2488 | 7mer-m8 | -0.02 | 39 | -0.02 | 0 | N/A |
| hsa-miR-6765-3p | 2485-2491 | 7mer-m8 | -0.02 | 50 | -0.02 | 0 | N/A |
| hsa-miR-29a-3p | 2490-2496 | 7mer-m8 | -0.02 | 21 | -0.02 | 2.424 | < 0.1 |
| hsa-miR-29c-3p | 2490-2496 | 7mer-m8 | -0.02 | 21 | -0.02 | 2.424 | < 0.1 |
| hsa-miR-29b-3p | 2490-2496 | 7mer-m8 | -0.02 | 20 | -0.02 | 2.424 | < 0.1 |
| hsa-miR-1236-3p | 2498-2505 | 8mer | -0.07 | 69 | -0.07 | 0 | N/A |
| hsa-miR-6515-3p | 2499-2505 | 7mer-1A | -0.01 | 23 | -0.01 | 0 | N/A |
| hsa-miR-452-3p | 2503-2509 | 7mer-1A | -0.02 | 48 | -0.02 | 0 | N/A |
| hsa-miR-7161-3p | 2509-2515 | 7mer-m8 | -0.06 | 71 | -0.06 | 0 | N/A |
| hsa-miR-3156-5p | 2510-2516 | 7mer-m8 | -0.02 | 37 | -0.02 | 0 | N/A |
| hsa-miR-4699-5p | 2512-2518 | 7mer-1A | -0.01 | 22 | -0.01 | 0 | N/A |
| hsa-miR-8060 | 2514-2520 | 7mer-m8 | -0.02 | 49 | -0.02 | 0 | N/A |
| hsa-miR-4261 | 2523-2530 | 8mer | -0.03 | 39 | -0.03 | 0 | N/A |
| hsa-miR-194-3p | 2528-2534 | 7mer-1A | -0.03 | 62 | -0.03 | 0 | N/A |
| hsa-miR-5693 | 2528-2534 | 7mer-1A | -0.01 | 43 | -0.01 | 0 | N/A |
| hsa-miR-4276 | 2529-2535 | 7mer-1A | -0.05 | 52 | -0.05 | 0 | N/A |
| hsa-miR-30a-3p | 2530-2536 | 7mer-m8 | -0.02 | 54 | -0.02 | 0.075 | N/A |
| hsa-miR-30d-3p | 2530-2536 | 7mer-m8 | -0.02 | 54 | -0.02 | 0.075 | N/A |
| hsa-miR-30e-3p | 2530-2536 | 7mer-m8 | -0.02 | 54 | -0.02 | 0.075 | N/A |
| hsa-miR-205-3p | 2531-2537 | 7mer-m8 | -0.02 | 69 | -0.02 | 0 | N/A |
| hsa-miR-3912-5p | 2536-2542 | 7mer-m8 | -0.07 | 70 | -0.07 | 0 | N/A |
| hsa-miR-4669 | 2538-2544 | 7mer-1A | -0.2 | 91 | -0.2 | 0 | N/A |
| hsa-miR-3657 | 2538-2544 | 7mer-1A | -0.07 | 56 | -0.07 | 0 | N/A |
| hsa-miR-511-3p | 2540-2546 | 7mer-1A | -0.05 | 56 | -0.05 | 0.072 | N/A |
| hsa-miR-548e-5p | 2556-2562 | 7mer-m8 | -0.02 | 39 | -0.02 | 0 | N/A |
| hsa-miR-3613-3p | 2560-2566 | 7mer-m8 | -0.02 | 36 | -0.02 | 0 | N/A |
| hsa-miR-4799-5p | 2568-2574 | 7mer-1A | -0.01 | 35 | -0.01 | 0 | N/A |
| hsa-miR-4263 | 2569-2575 | 7mer-1A | -0.01 | 40 | -0.01 | 0 | N/A |
| hsa-miR-576-5p | 2569-2575 | 7mer-1A | -0.01 | 37 | -0.01 | 0.072 | N/A |
| hsa-miR-4781-3p | 2582-2588 | 7mer-1A | -0.06 | 75 | -0.06 | 0 | N/A |
| hsa-miR-6756-3p | 2596-2602 | 7mer-m8 | -0.16 | 79 | -0.16 | 0 | N/A |
| hsa-miR-3127-3p | 2596-2602 | 7mer-m8 | -0.15 | 78 | -0.15 | 0 | N/A |
| hsa-miR-7856-5p | 2606-2612 | 7mer-1A | -0.01 | 40 | -0.01 | 0 | N/A |
| hsa-miR-4328 | 2609-2615 | 7mer-m8 | -0.02 | 47 | -0.02 | 0 | N/A |
| hsa-miR-140-3p.1 | 2613-2619 | 7mer-1A | -0.09 | 76 | -0.09 | 1.218 | < 0.1 |
| hsa-miR-4639-3p | 2618-2624 | 7mer-m8 | -0.06 | 74 | -0.06 | 0.072 | N/A |
| hsa-miR-6777-3p | 2619-2625 | 7mer-m8 | -0.02 | 39 | -0.02 | 0 | N/A |
| hsa-miR-4286 | 2621-2628 | 8mer | -0.17 | 89 | -0.17 | 0 | N/A |
| hsa-miR-4680-5p | 2628-2634 | 7mer-m8 | -0.03 | 44 | -0.03 | 0 | N/A |
| hsa-miR-6716-3p | 2630-2637 | 8mer | -0.44 | 94 | -0.44 | 0 | N/A |
| hsa-miR-3124-3p | 2635-2641 | 7mer-1A | -0.07 | 74 | -0.07 | 0 | N/A |
| hsa-miR-548d-3p | 2647-2653 | 7mer-1A | -0.07 | 94 | -0.07 | 0.072 | N/A |
| hsa-miR-548ac | 2647-2653 | 7mer-1A | -0.01 | 57 | -0.01 | 0.072 | N/A |
| hsa-miR-548z | 2647-2653 | 7mer-1A | -0.01 | 57 | -0.01 | 0.072 | N/A |
| hsa-miR-548bb-3p | 2647-2653 | 7mer-1A | -0.01 | 57 | -0.01 | 0.072 | N/A |
| hsa-miR-548h-3p | 2647-2653 | 7mer-1A | -0.01 | 57 | -0.01 | 0.072 | N/A |
| hsa-miR-548ah-3p | 2647-2653 | 7mer-1A | -0.01 | 50 | -0.01 | 0.072 | N/A |
| hsa-miR-548aq-3p | 2647-2653 | 7mer-1A | -0.01 | 50 | -0.01 | 0.072 | N/A |
| hsa-miR-548am-3p | 2647-2653 | 7mer-1A | -0.01 | 50 | -0.01 | 0.072 | N/A |
| hsa-miR-548j-3p | 2647-2653 | 7mer-1A | -0.01 | 50 | -0.01 | 0.072 | N/A |
| hsa-miR-548ae-3p | 2647-2653 | 7mer-1A | -0.01 | 50 | -0.01 | 0.072 | N/A |
| hsa-miR-548aj-3p | 2647-2653 | 7mer-1A | -0.01 | 49 | -0.01 | 0.072 | N/A |
| hsa-miR-548x-3p | 2647-2653 | 7mer-1A | -0.01 | 49 | -0.01 | 0.072 | N/A |
| hsa-miR-3163 | 2649-2655 | 7mer-1A | -0.01 | 45 | -0.01 | 0 | N/A |
| hsa-miR-410-3p | 2651-2657 | 7mer-1A | -0.01 | 55 | -0.01 | 0.426 | N/A |
| hsa-miR-5011-5p | 2652-2659 | 8mer | -0.03 | 56 | -0.03 | 0 | N/A |
| hsa-miR-190a-3p | 2653-2660 | 8mer | -0.03 | 69 | -0.03 | 0.072 | N/A |
| hsa-miR-6083 | 2655-2661 | 7mer-1A | -0.01 | 45 | -0.01 | 0 | N/A |
| hsa-miR-190a-3p | 2662-2668 | 7mer-m8 | -0.02 | 40 | -0.02 | 0.072 | N/A |
| hsa-miR-5011-5p | 2662-2668 | 7mer-1A | -0.01 | 24 | -0.01 | 0 | N/A |
| hsa-miR-5011-5p | 2663-2669 | 7mer-m8 | -0.02 | 37 | -0.02 | 0 | N/A |
| hsa-miR-1323 | 2674-2680 | 7mer-m8 | -0.02 | 52 | -0.02 | 0.075 | N/A |
| hsa-miR-548o-3p | 2674-2680 | 7mer-m8 | -0.02 | 51 | -0.02 | 0.075 | N/A |
| hsa-miR-188-3p | 2679-2685 | 7mer-m8 | -0.09 | 65 | -0.09 | 0 | N/A |
| hsa-miR-2355-5p | 2686-2692 | 7mer-1A | -0.16 | 84 | -0.16 | 0 | N/A |
| hsa-miR-1236-3p | 2689-2696 | 8mer | -0.17 | 87 | -0.17 | 0 | N/A |
| hsa-miR-6515-3p | 2690-2696 | 7mer-1A | -0.09 | 75 | -0.09 | 0 | N/A |
| hsa-miR-216b-5p | 2692-2698 | 7mer-m8 | -0.08 | 77 | -0.08 | 0.898 | < 0.1 |
| hsa-miR-4668-3p | 2695-2701 | 7mer-1A | -0.01 | 56 | -0.01 | 0 | N/A |
| hsa-miR-5692a | 2706-2712 | 7mer-1A | -0.01 | 56 | -0.01 | 0 | N/A |
| hsa-miR-514b-5p | 2714-2721 | 8mer | -0.09 | 87 | -0.09 | 0.072 | N/A |
| hsa-miR-513c-5p | 2714-2721 | 8mer | -0.08 | 84 | -0.08 | 0.072 | N/A |
| hsa-miR-6830-3p | 2719-2725 | 7mer-m8 | -0.08 | 80 | -0.08 | 0 | N/A |
| hsa-miR-4474-5p | 2722-2728 | 7mer-1A | -0.02 | 34 | -0.02 | 0 | N/A |
| hsa-miR-3197 | 2729-2735 | 7mer-m8 | -0.32 | 96 | -0.32 | 0 | N/A |
| hsa-miR-3117-5p | 2737-2744 | 8mer | -0.08 | 59 | -0.08 | 0 | N/A |
| hsa-miR-6730-3p | 2739-2745 | 7mer-m8 | -0.02 | 51 | -0.02 | 0 | N/A |
| hsa-miR-4732-3p | 2741-2748 | 8mer | -0.05 | 78 | -0.05 | 0 | N/A |
| hsa-miR-345-3p | 2742-2748 | 7mer-1A | -0.04 | 64 | -0.04 | 0 | N/A |
| hsa-miR-670-5p | 2742-2749 | 8mer | -0.03 | 61 | -0.03 | 0 | N/A |
| hsa-miR-125b-5p | 2742-2748 | 7mer-1A | -0.03 | 39 | -0.03 | 1.3 | < 0.1 |
| hsa-miR-4319 | 2742-2748 | 7mer-1A | -0.01 | 21 | -0.01 | 1.3 | < 0.1 |
| hsa-miR-125a-5p | 2742-2748 | 7mer-1A | -0.01 | 21 | -0.01 | 1.3 | < 0.1 |
| hsa-miR-891b | 2748-2754 | 7mer-m8 | -0.02 | 25 | -0.02 | 0.075 | N/A |
| hsa-miR-1827 | 2752-2758 | 7mer-1A | -0.06 | 51 | -0.06 | 0.411 | N/A |
| hsa-miR-4649-3p | 2753-2759 | 7mer-m8 | -0.12 | 83 | -0.12 | 0 | N/A |
| hsa-miR-6090 | 2761-2767 | 7mer-1A | -0.06 | 70 | -0.06 | 0 | N/A |
| hsa-miR-4300 | 2761-2767 | 7mer-1A | -0.1 | 66 | -0.1 | 0.075 | N/A |
| hsa-miR-6827-5p | 2761-2767 | 7mer-1A | -0.1 | 64 | -0.1 | 0.072 | N/A |
| hsa-miR-6726-5p | 2761-2767 | 7mer-1A | -0.08 | 58 | -0.08 | 0.075 | N/A |
| hsa-miR-920 | 2761-2767 | 7mer-1A | -0.05 | 54 | -0.05 | 0.075 | N/A |
| hsa-miR-5591-5p | 2761-2767 | 7mer-1A | -0.04 | 37 | -0.04 | 0.075 | N/A |
| hsa-miR-3192-5p | 2762-2769 | 8mer | -0.03 | 57 | -0.03 | 0.021 | N/A |
| hsa-miR-6506-5p | 2763-2769 | 7mer-1A | -0.04 | 71 | -0.04 | 0 | N/A |
| hsa-miR-4314 | 2763-2770 | 8mer | -0.05 | 46 | -0.05 | 0 | N/A |
| hsa-miR-4646-5p | 2763-2769 | 7mer-1A | -0.03 | 46 | -0.03 | 0 | N/A |
| hsa-miR-619-5p | 2763-2769 | 7mer-1A | -0.01 | 44 | -0.01 | 0 | N/A |
| hsa-miR-204-3p | 2763-2769 | 7mer-1A | -0.01 | 39 | -0.01 | 0 | N/A |
| hsa-miR-6505-3p | 2767-2773 | 7mer-m8 | -0.02 | 26 | -0.02 | 0.072 | N/A |
| hsa-miR-8083 | 2769-2775 | 7mer-m8 | -0.02 | 35 | -0.02 | 0 | N/A |
| hsa-miR-4700-3p | 2771-2777 | 7mer-m8 | -0.03 | 63 | -0.03 | 0 | N/A |
| hsa-miR-513a-5p | 2773-2779 | 7mer-m8 | -0.02 | 45 | -0.02 | 0.075 | N/A |
| hsa-miR-4704-3p | 2776-2782 | 7mer-m8 | -0.07 | 73 | -0.07 | 0 | N/A |
| hsa-miR-27a-3p | 2779-2785 | 7mer-m8 | -0.02 | 47 | -0.02 | 0.179 | < 0.1 |
| hsa-miR-27b-3p | 2779-2785 | 7mer-m8 | -0.02 | 46 | -0.02 | 0.179 | < 0.1 |
| hsa-miR-128-3p | 2779-2785 | 7mer-1A | -0.01 | 31 | -0.01 | 0.179 | < 0.1 |
| hsa-miR-216a-3p | 2779-2785 | 7mer-1A | -0.01 | 30 | -0.01 | 0.179 | < 0.1 |
| hsa-miR-3681-3p | 2779-2785 | 7mer-1A | -0.01 | 28 | -0.01 | 0.179 | < 0.1 |
| hsa-miR-4257 | 2790-2796 | 7mer-m8 | -0.02 | 48 | -0.02 | 0 | N/A |
| hsa-miR-1324 | 2794-2800 | 7mer-m8 | -0.02 | 41 | -0.02 | 0 | N/A |
| hsa-miR-3166 | 2795-2801 | 7mer-m8 | -0.02 | 14 | -0.02 | 0 | N/A |
| hsa-miR-922 | 2797-2803 | 7mer-m8 | -0.02 | 52 | -0.02 | 0.075 | N/A |
| hsa-miR-107 | 2799-2805 | 7mer-1A | -0.01 | 18 | -0.01 | 0.171 | < 0.1 |
| hsa-miR-103a-3p | 2799-2805 | 7mer-1A | -0.01 | 18 | -0.01 | 0.171 | < 0.1 |
| hsa-miR-3944-5p | 2800-2806 | 7mer-m8 | -0.08 | 76 | -0.08 | 0 | N/A |
| hsa-miR-548q | 2803-2809 | 7mer-m8 | -0.02 | 59 | -0.02 | 0 | N/A |
| hsa-miR-138-5p | 2804-2810 | 7mer-m8 | -0.03 | 39 | -0.03 | 0.382 | < 0.1 |
| hsa-miR-1207-3p | 2806-2812 | 7mer-m8 | -0.02 | 43 | -0.02 | 0.021 | N/A |
| hsa-miR-2355-5p | 2810-2816 | 7mer-m8 | -0.02 | 30 | -0.02 | 0 | N/A |
| hsa-miR-4786-3p | 2820-2826 | 7mer-m8 | -0.02 | 18 | -0.02 | 0 | N/A |
| hsa-miR-671-5p | 2821-2828 | 8mer | -0.04 | 52 | -0.04 | 0.072 | N/A |
| hsa-miR-6079 | 2822-2828 | 7mer-m8 | -0.02 | 57 | -0.02 | 0 | N/A |
| hsa-miR-6828-5p | 2822-2828 | 7mer-1A | -0.01 | 12 | -0.01 | 0.072 | N/A |
| hsa-miR-6128 | 2824-2830 | 7mer-1A | -0.01 | 33 | -0.01 | 0.075 | N/A |
| hsa-miR-1299 | 2824-2830 | 7mer-m8 | -0.02 | 24 | -0.02 | 0.021 | N/A |
| hsa-miR-875-3p | 2824-2830 | 7mer-1A | -0.01 | 24 | -0.01 | 0.072 | N/A |
| hsa-miR-518c-5p | 2825-2831 | 7mer-m8 | -0.02 | 31 | -0.02 | 0 | N/A |
| hsa-miR-6796-3p | 2828-2834 | 7mer-m8 | -0.02 | 47 | -0.02 | 0 | N/A |
| hsa-miR-3153 | 2832-2839 | 8mer | -0.03 | 67 | -0.03 | 0 | N/A |
| hsa-miR-6733-5p | 2832-2839 | 8mer | -0.03 | 53 | -0.03 | 0 | N/A |
| hsa-miR-6739-5p | 2832-2839 | 8mer | -0.03 | 52 | -0.03 | 0 | N/A |
| hsa-miR-4668-5p | 2833-2839 | 7mer-1A | -0.01 | 15 | -0.01 | 0 | N/A |
| hsa-miR-204-3p | 2834-2840 | 7mer-m8 | -0.02 | 50 | -0.02 | 0 | N/A |
| hsa-miR-4646-5p | 2834-2840 | 7mer-m8 | -0.02 | 33 | -0.02 | 0 | N/A |
| hsa-miR-4292 | 2836-2842 | 7mer-m8 | -0.15 | 80 | -0.15 | 0 | N/A |
| hsa-miR-6791-5p | 2836-2842 | 7mer-m8 | -0.15 | 80 | -0.15 | 0 | N/A |
| hsa-miR-504-5p.1 | 2837-2843 | 7mer-m8 | -0.07 | 77 | -0.07 | 1.403 | N/A |
| hsa-miR-3620-3p | 2838-2844 | 7mer-m8 | -0.11 | 78 | -0.11 | 0 | N/A |
| hsa-miR-640 | 2847-2853 | 7mer-m8 | -0.12 | 77 | -0.12 | 0.075 | N/A |
| hsa-miR-3151-3p | 2848-2855 | 8mer | -0.27 | 93 | -0.27 | 0 | N/A |
| hsa-miR-3192-3p | 2849-2855 | 7mer-m8 | -0.05 | 74 | -0.05 | 0 | N/A |
| hsa-miR-7641 | 2853-2859 | 7mer-1A | -0.01 | 38 | -0.01 | 0 | N/A |
| hsa-miR-4282 | 2858-2864 | 7mer-m8 | -0.02 | 66 | -0.02 | 0 | N/A |
| hsa-miR-5004-5p | 2864-2870 | 7mer-m8 | -0.09 | 53 | -0.09 | 0 | N/A |
| hsa-miR-6758-5p | 2867-2873 | 7mer-m8 | -0.04 | 62 | -0.04 | 0 | N/A |
| hsa-miR-6856-5p | 2867-2873 | 7mer-m8 | -0.02 | 44 | -0.02 | 0 | N/A |
| hsa-miR-6793-3p | 2873-2879 | 7mer-1A | -0.01 | 39 | -0.01 | 0 | N/A |
| hsa-miR-4274 | 2878-2884 | 7mer-m8 | -0.16 | 89 | -0.16 | 0 | N/A |
| hsa-miR-548e-5p | 2881-2887 | 7mer-m8 | -0.02 | 39 | -0.02 | 0 | N/A |
| hsa-miR-577 | 2886-2892 | 7mer-m8 | -0.02 | 38 | -0.02 | 0.075 | N/A |
| hsa-miR-3942-3p | 2889-2895 | 7mer-m8 | -0.05 | 78 | -0.05 | 0 | N/A |
| hsa-miR-5100 | 2889-2895 | 7mer-1A | -0.01 | 38 | -0.01 | 0 | N/A |
| hsa-miR-892c-5p | 2890-2896 | 7mer-m8 | -0.09 | 80 | -0.09 | 0.075 | N/A |
| hsa-miR-4760-3p | 2892-2898 | 7mer-1A | -0.01 | 40 | -0.01 | 0 | N/A |
| hsa-miR-1252-3p | 2899-2905 | 7mer-1A | -0.01 | 53 | -0.01 | 0 | N/A |
| hsa-miR-3646 | 2899-2905 | 7mer-1A | -0.01 | 37 | -0.01 | 0 | N/A |
| hsa-miR-6831-3p | 2903-2909 | 7mer-1A | -0.02 | 47 | -0.02 | 0 | N/A |
| hsa-miR-6813-3p | 2908-2914 | 7mer-1A | -0.07 | 66 | -0.07 | 0 | N/A |
| hsa-miR-3200-3p | 2908-2914 | 7mer-1A | -0.03 | 56 | -0.03 | 0.075 | N/A |
| hsa-miR-3163 | 2923-2929 | 7mer-1A | -0.01 | 45 | -0.01 | 0 | N/A |
| hsa-miR-568 | 2925-2931 | 7mer-m8 | -0.03 | 42 | -0.03 | 0.021 | N/A |
| hsa-miR-3924 | 2927-2933 | 7mer-m8 | -0.02 | 39 | -0.02 | 0 | N/A |
| hsa-miR-5002-5p | 2933-2939 | 7mer-m8 | -0.02 | 45 | -0.02 | 0 | N/A |
| hsa-miR-136-5p | 2937-2944 | 8mer | -0.13 | 84 | -0.13 | 0.751 | N/A |
| hsa-miR-6762-3p | 2946-2952 | 7mer-m8 | -0.24 | 91 | -0.24 | 0 | N/A |
| hsa-miR-485-5p | 2948-2954 | 7mer-m8 | -0.1 | 63 | -0.1 | 1.523 | N/A |
| hsa-miR-6884-5p | 2948-2954 | 7mer-m8 | -0.08 | 57 | -0.08 | 1.523 | N/A |
| hsa-miR-6888-5p | 2953-2959 | 7mer-1A | -0.01 | 32 | -0.01 | 0 | N/A |
| hsa-miR-7844-5p | 2957-2963 | 7mer-m8 | -0.02 | 61 | -0.02 | 0 | N/A |
| hsa-miR-5582-3p | 2959-2965 | 7mer-1A | -0.01 | 47 | -0.01 | 0 | N/A |
| hsa-miR-548a-3p | 2959-2965 | 7mer-1A | -0.01 | 47 | -0.01 | 0.021 | N/A |
| hsa-miR-548e-3p | 2959-2965 | 7mer-1A | -0.01 | 46 | -0.01 | 0.021 | N/A |
| hsa-miR-548az-3p | 2959-2965 | 7mer-1A | -0.01 | 46 | -0.01 | 0.021 | N/A |
| hsa-miR-548ar-3p | 2959-2965 | 7mer-1A | -0.01 | 46 | -0.01 | 0.021 | N/A |
| hsa-miR-548f-3p | 2959-2965 | 7mer-1A | -0.01 | 45 | -0.01 | 0.021 | N/A |
| hsa-miR-510-3p | 2966-2972 | 7mer-1A | -0.01 | 43 | -0.01 | 0 | N/A |
| hsa-miR-2052 | 2968-2974 | 7mer-m8 | -0.02 | 30 | -0.02 | 0 | N/A |
| hsa-miR-8066 | 2978-2985 | 8mer | -0.12 | 85 | -0.12 | 0 | N/A |
| hsa-miR-1228-3p | 2989-2995 | 7mer-1A | -0.02 | 39 | -0.02 | 0 | N/A |
| hsa-miR-377-3p | 2990-2996 | 7mer-1A | -0.09 | 64 | -0.09 | 1.148 | N/A |
| hsa-miR-192-3p | 3004-3010 | 7mer-1A | -0.02 | 38 | -0.02 | 0 | N/A |
| hsa-miR-4776-3p | 3005-3011 | 7mer-1A | -0.07 | 64 | -0.07 | 0 | N/A |
| hsa-miR-5087 | 3008-3014 | 7mer-1A | -0.01 | 28 | -0.01 | 0 | N/A |
| hsa-miR-29b-2-5p | 3009-3015 | 7mer-1A | -0.01 | 40 | -0.01 | 0 | N/A |
| hsa-miR-4429 | 3016-3022 | 7mer-m8 | -0.02 | 48 | -0.02 | 1.561 | N/A |
| hsa-miR-320a | 3016-3022 | 7mer-m8 | -0.02 | 48 | -0.02 | 1.561 | N/A |
| hsa-miR-320c | 3016-3022 | 7mer-m8 | -0.02 | 48 | -0.02 | 1.561 | N/A |
| hsa-miR-320d | 3016-3022 | 7mer-m8 | -0.02 | 48 | -0.02 | 1.561 | N/A |
| hsa-miR-320b | 3016-3022 | 7mer-m8 | -0.02 | 48 | -0.02 | 1.561 | N/A |
| hsa-miR-4786-3p | 3022-3028 | 7mer-m8 | -0.07 | 58 | -0.07 | 0 | N/A |
| hsa-miR-4499 | 3045-3051 | 7mer-m8 | -0.1 | 78 | -0.1 | 0 | N/A |
| hsa-miR-4494 | 3046-3053 | 8mer | -0.25 | 95 | -0.25 | 0 | N/A |
| hsa-miR-499b-5p | 3047-3053 | 7mer-1A | -0.05 | 61 | -0.05 | 0 | N/A |
| hsa-miR-1283 | 3065-3071 | 7mer-m8 | -0.02 | 20 | -0.02 | 0.021 | N/A |
| hsa-miR-4999-3p | 3068-3074 | 7mer-1A | -0.04 | 52 | -0.04 | 0 | N/A |
| hsa-miR-6739-3p | 3073-3079 | 7mer-1A | -0.03 | 44 | -0.03 | 0 | N/A |
| hsa-miR-375 | 3073-3079 | 7mer-1A | -0.01 | 36 | -0.01 | 3.525 | 0.33 |
| hsa-miR-1305 | 3080-3086 | 7mer-m8 | -0.02 | 78 | -0.02 | 0 | N/A |
| hsa-miR-33a-3p | 3084-3090 | 7mer-m8 | -0.02 | 49 | -0.02 | 0 | N/A |
| hsa-miR-6501-3p | 3091-3097 | 7mer-m8 | -0.06 | 70 | -0.06 | 0 | N/A |
| hsa-miR-520a-5p | 3093-3099 | 7mer-m8 | -0.08 | 70 | -0.08 | 0.347 | N/A |
| hsa-miR-525-5p | 3093-3099 | 7mer-m8 | -0.08 | 69 | -0.08 | 0.347 | N/A |
| hsa-miR-198 | 3094-3100 | 7mer-m8 | -0.06 | 63 | -0.06 | 0.075 | N/A |
| hsa-miR-6723-5p | 3097-3103 | 7mer-1A | -0.19 | 87 | -0.19 | 0 | N/A |
| hsa-miR-4540 | 3097-3103 | 7mer-1A | -0.11 | 71 | -0.11 | 0 | N/A |
| hsa-miR-103a-2-5p | 3103-3109 | 7mer-m8 | -0.02 | 33 | -0.02 | 0.072 | N/A |
| hsa-miR-6815-3p | 3104-3110 | 7mer-m8 | -0.13 | 77 | -0.13 | 0 | N/A |
| hsa-miR-645 | 3107-3113 | 7mer-m8 | -0.1 | 66 | -0.1 | 0.021 | N/A |
| hsa-miR-497-3p | 3113-3119 | 7mer-m8 | -0.02 | 68 | -0.02 | 0.072 | N/A |
| hsa-miR-6715a-3p | 3114-3120 | 7mer-m8 | -0.02 | 25 | -0.02 | 0 | N/A |
| hsa-miR-4701-5p | 3122-3129 | 8mer | -0.06 | 77 | -0.06 | 0.021 | N/A |
| hsa-miR-588 | 3122-3129 | 8mer | -0.05 | 74 | -0.05 | 0.021 | N/A |
| hsa-miR-4435 | 3123-3129 | 7mer-1A | -0.03 | 43 | -0.03 | 0 | N/A |
| hsa-miR-3187-3p | 3123-3129 | 7mer-1A | -0.01 | 32 | -0.01 | 0 | N/A |
| hsa-miR-548s | 3123-3129 | 7mer-1A | -0.02 | 23 | -0.02 | 0 | N/A |
| hsa-miR-4715-5p | 3125-3131 | 7mer-m8 | -0.02 | 33 | -0.02 | 0.072 | N/A |
| hsa-miR-571 | 3126-3132 | 7mer-m8 | -0.02 | 24 | -0.02 | 0.021 | N/A |
| hsa-miR-4653-3p | 3128-3134 | 7mer-1A | -0.01 | 13 | -0.01 | 0 | N/A |
| hsa-miR-6514-5p | 3129-3135 | 7mer-m8 | -0.02 | 34 | -0.02 | 0 | N/A |
| hsa-miR-4534 | 3130-3136 | 7mer-m8 | -0.02 | 38 | -0.02 | 0 | N/A |
| hsa-miR-8082 | 3130-3136 | 7mer-m8 | -0.02 | 26 | -0.02 | 0 | N/A |
| hsa-miR-4520-5p | 3148-3155 | 8mer | -0.26 | 79 | -0.26 | 0 | N/A |
| hsa-miR-4288 | 3151-3157 | 7mer-m8 | -0.05 | 70 | -0.05 | 0.021 | N/A |
| hsa-miR-632 | 3151-3157 | 7mer-m8 | -0.02 | 62 | -0.02 | 0.021 | N/A |
| hsa-miR-346 | 3151-3157 | 7mer-1A | -0.01 | 14 | -0.01 | 0.021 | N/A |
| hsa-miR-3120-5p | 3152-3159 | 8mer | -0.03 | 57 | -0.03 | 0 | N/A |
| hsa-miR-6888-3p | 3153-3159 | 7mer-m8 | -0.02 | 35 | -0.02 | 0 | N/A |
| hsa-miR-7107-3p | 3155-3161 | 7mer-m8 | -0.02 | 28 | -0.02 | 0 | N/A |
| hsa-miR-6753-3p | 3155-3161 | 7mer-m8 | -0.02 | 25 | -0.02 | 0 | N/A |
| hsa-miR-1182 | 3157-3163 | 7mer-m8 | -0.02 | 32 | -0.02 | 0.021 | N/A |
| hsa-miR-4797-5p | 3162-3168 | 7mer-1A | -0.01 | 52 | -0.01 | 0 | N/A |
| hsa-let-7f-2-3p | 3164-3170 | 7mer-m8 | -0.07 | 80 | -0.07 | 0 | N/A |
| hsa-miR-1185-2-3p | 3164-3170 | 7mer-m8 | -0.02 | 52 | -0.02 | 0 | N/A |
| hsa-miR-1185-1-3p | 3164-3170 | 7mer-m8 | -0.02 | 51 | -0.02 | 0 | N/A |
| hsa-miR-298 | 3172-3178 | 7mer-1A | -0.12 | 79 | -0.12 | 0.075 | N/A |
| hsa-miR-5700 | 3175-3181 | 7mer-1A | -0.01 | 36 | -0.01 | 0 | N/A |
| hsa-miR-6507-5p | 3180-3186 | 7mer-m8 | -0.02 | 59 | -0.02 | 0 | N/A |
| hsa-miR-4428 | 3188-3194 | 7mer-m8 | -0.09 | 75 | -0.09 | 0.021 | N/A |
| hsa-miR-4766-5p | 3194-3200 | 7mer-1A | -0.01 | 43 | -0.01 | 0.072 | N/A |
| hsa-miR-4527 | 3197-3203 | 7mer-1A | -0.15 | 83 | -0.15 | 0 | N/A |
| hsa-miR-6503-5p | 3197-3203 | 7mer-1A | -0.14 | 80 | -0.14 | 0 | N/A |
| hsa-miR-6753-3p | 3197-3203 | 7mer-1A | -0.05 | 48 | -0.05 | 0 | N/A |
| hsa-miR-7107-3p | 3197-3203 | 7mer-1A | -0.04 | 41 | -0.04 | 0 | N/A |
| hsa-miR-2681-3p | 3201-3207 | 7mer-m8 | -0.06 | 70 | -0.06 | 0 | N/A |
| hsa-miR-4529-5p | 3205-3212 | 8mer | -0.35 | 95 | -0.35 | 0 | N/A |
| hsa-miR-3187-3p | 3206-3213 | 8mer | -0.17 | 94 | -0.17 | 0 | N/A |
| hsa-miR-548s | 3207-3213 | 7mer-1A | -0.12 | 82 | -0.12 | 0 | N/A |
| hsa-miR-4435 | 3207-3213 | 7mer-1A | -0.1 | 72 | -0.1 | 0 | N/A |
| hsa-miR-588 | 3207-3213 | 7mer-1A | -0.03 | 66 | -0.03 | 0.021 | N/A |
| hsa-miR-4701-5p | 3207-3213 | 7mer-1A | -0.01 | 38 | -0.01 | 0.021 | N/A |
| hsa-miR-1279 | 3211-3217 | 7mer-m8 | -0.02 | 57 | -0.02 | 0 | N/A |
| hsa-miR-136-5p | 3215-3221 | 7mer-1A | -0.06 | 67 | -0.06 | 0.156 | N/A |
| hsa-miR-216b-5p | 3218-3224 | 7mer-1A | -0.08 | 76 | -0.08 | 0.060 | < 0.1 |
| hsa-miR-216a-5p | 3218-3224 | 7mer-1A | -0.05 | 62 | -0.05 | 0.060 | < 0.1 |
| hsa-miR-4502 | 3232-3238 | 7mer-m8 | -0.02 | 40 | -0.02 | 0.409 | N/A |
| hsa-miR-3136-5p | 3235-3241 | 7mer-m8 | -0.13 | 80 | -0.13 | 0.021 | N/A |
| hsa-miR-4439 | 3235-3241 | 7mer-m8 | -0.07 | 75 | -0.07 | 0.021 | N/A |
| hsa-miR-6855-3p | 3235-3241 | 7mer-1A | -0.04 | 54 | -0.04 | 0 | N/A |
| hsa-miR-6857-3p | 3235-3241 | 7mer-1A | -0.05 | 51 | -0.05 | 0 | N/A |
| hsa-miR-4513 | 3235-3241 | 7mer-1A | -0.03 | 44 | -0.03 | 0 | N/A |
| hsa-miR-4770 | 3239-3245 | 7mer-m8 | -0.08 | 71 | -0.08 | 0.576 | < 0.1 |
| hsa-miR-143-3p | 3239-3245 | 7mer-m8 | -0.08 | 70 | -0.08 | 0.576 | < 0.1 |
| hsa-miR-6088 | 3239-3245 | 7mer-m8 | -0.03 | 42 | -0.03 | 0.576 | < 0.1 |
| hsa-miR-4311 | 3242-3249 | 8mer | -0.03 | 78 | -0.03 | 0 | N/A |
| hsa-miR-1276 | 3243-3249 | 7mer-1A | -0.01 | 54 | -0.01 | 0.021 | N/A |
| hsa-miR-583 | 3243-3249 | 7mer-1A | -0.01 | 49 | -0.01 | 0.075 | N/A |
| hsa-miR-224-5p | 3252-3258 | 7mer-m8 | -0.02 | 60 | -0.02 | 1.067 | N/A |
| hsa-miR-9500 | 3256-3262 | 7mer-m8 | -0.07 | 70 | -0.07 | 0 | N/A |
| hsa-miR-527 | 3261-3268 | 8mer | -0.03 | 68 | -0.03 | 0.021 | N/A |
| hsa-miR-518a-5p | 3261-3268 | 8mer | -0.03 | 68 | -0.03 | 0.021 | N/A |
| hsa-miR-548aw | 3262-3268 | 7mer-1A | -0.01 | 36 | -0.01 | 0 | N/A |
| hsa-miR-450b-5p | 3263-3269 | 7mer-m8 | -0.02 | 36 | -0.02 | 0 | N/A |
| hsa-miR-5087 | 3266-3272 | 7mer-1A | -0.01 | 28 | -0.01 | 0 | N/A |
| hsa-miR-29b-1-5p | 3267-3273 | 7mer-m8 | -0.02 | 43 | -0.02 | 0 | N/A |
| hsa-miR-5587-5p | 3277-3284 | 8mer | -0.1 | 76 | -0.1 | 0 | N/A |
| hsa-miR-4717-3p | 3287-3293 | 7mer-1A | -0.03 | 58 | -0.03 | 0 | N/A |
| hsa-miR-342-3p | 3289-3295 | 7mer-1A | -0.01 | 39 | -0.01 | 0.060 | N/A |
| hsa-miR-193a-5p | 3293-3299 | 7mer-1A | -0.02 | 30 | -0.02 | 0.373 | < 0.1 |
| hsa-miR-4515 | 3297-3303 | 7mer-m8 | -0.1 | 63 | -0.1 | 0 | N/A |
| hsa-miR-3169 | 3298-3304 | 7mer-m8 | -0.07 | 66 | -0.07 | 0 | N/A |
| hsa-miR-4700-3p | 3300-3306 | 7mer-m8 | -0.11 | 85 | -0.11 | 0 | N/A |
| hsa-miR-548ap-3p | 3308-3314 | 7mer-m8 | -0.02 | 37 | -0.02 | 0 | N/A |
| hsa-miR-548t-3p | 3308-3314 | 7mer-m8 | -0.02 | 36 | -0.02 | 0 | N/A |
| hsa-miR-548aa | 3308-3314 | 7mer-m8 | -0.02 | 36 | -0.02 | 0 | N/A |
| hsa-miR-191-5p | 3316-3322 | 7mer-m8 | -0.04 | 8 | -0.04 | 0.179 | < 0.1 |
| hsa-miR-218-1-3p | 3323-3329 | 7mer-m8 | -0.06 | 59 | -0.06 | 0 | N/A |
| hsa-miR-203a-5p | 3324-3330 | 7mer-m8 | -0.02 | 30 | -0.02 | 0 | N/A |
| hsa-miR-876-3p | 3325-3331 | 7mer-m8 | -0.02 | 23 | -0.02 | 0 | N/A |
| hsa-miR-1293 | 3326-3333 | 8mer | -0.21 | 92 | -0.21 | 0 | N/A |
| hsa-miR-4483 | 3326-3333 | 8mer | -0.12 | 87 | -0.12 | 0 | N/A |
| hsa-miR-363-5p | 3327-3333 | 7mer-1A | -0.11 | 68 | -0.11 | 0.072 | N/A |
| hsa-miR-6745 | 3327-3333 | 7mer-1A | -0.09 | 62 | -0.09 | 0.072 | N/A |
| hsa-miR-6766-5p | 3327-3333 | 7mer-1A | -0.12 | 57 | -0.12 | 0 | N/A |
| hsa-miR-6756-5p | 3327-3333 | 7mer-1A | -0.14 | 57 | -0.14 | 0 | N/A |
| hsa-miR-6776-5p | 3328-3335 | 8mer | -0.03 | 48 | -0.03 | 0 | N/A |
| hsa-miR-6861-5p | 3329-3335 | 7mer-1A | -0.01 | 15 | -0.01 | 0 | N/A |
| hsa-miR-1231 | 3331-3337 | 7mer-m8 | -0.02 | 51 | -0.02 | 0 | N/A |
| hsa-miR-654-3p | 3332-3338 | 7mer-m8 | -0.03 | 48 | -0.03 | 0.448 | N/A |
| hsa-miR-6828-5p | 3340-3347 | 8mer | -0.03 | 32 | -0.03 | 0 | N/A |
| hsa-miR-6079 | 3341-3347 | 7mer-m8 | -0.08 | 80 | -0.08 | 0 | N/A |
| hsa-miR-671-5p | 3341-3347 | 7mer-1A | -0.05 | 56 | -0.05 | 0.072 | N/A |
| hsa-miR-3190-3p | 3342-3348 | 7mer-m8 | -0.02 | 29 | -0.02 | 0 | N/A |
| hsa-miR-363-5p | 3344-3351 | 8mer | -0.21 | 87 | -0.21 | 0 | N/A |
| hsa-miR-6745 | 3344-3351 | 8mer | -0.2 | 86 | -0.2 | 0 | N/A |
| hsa-miR-1293 | 3345-3351 | 7mer-1A | -0.17 | 88 | -0.17 | 0.021 | N/A |
| hsa-miR-4483 | 3345-3351 | 7mer-1A | -0.12 | 87 | -0.12 | 0.021 | N/A |
| hsa-miR-6766-5p | 3345-3351 | 7mer-1A | -0.26 | 87 | -0.26 | 0 | N/A |
| hsa-miR-6756-5p | 3345-3351 | 7mer-1A | -0.19 | 71 | -0.19 | 0 | N/A |
| hsa-miR-6776-5p | 3346-3352 | 7mer-m8 | -0.03 | 46 | -0.03 | 0 | N/A |
| hsa-miR-5589-5p | 3347-3353 | 7mer-m8 | -0.02 | 56 | -0.02 | 0 | N/A |
| hsa-miR-4505 | 3348-3355 | 8mer | -0.39 | 98 | -0.39 | 0 | N/A |
| hsa-miR-5787 | 3348-3355 | 8mer | -0.3 | 97 | -0.3 | 0 | N/A |
| hsa-miR-4430 | 3349-3355 | 7mer-1A | -0.15 | 87 | -0.15 | 0 | N/A |
| hsa-miR-6842-3p | 3349-3356 | 8mer | -0.17 | 83 | -0.17 | 0 | N/A |
| hsa-miR-3652 | 3349-3355 | 7mer-1A | -0.13 | 83 | -0.13 | 0 | N/A |
| hsa-miR-647 | 3350-3356 | 7mer-1A | -0.01 | 46 | -0.01 | 0 | N/A |
| hsa-miR-27a-5p | 3355-3361 | 7mer-1A | -0.09 | 56 | -0.09 | 0.072 | N/A |
| hsa-miR-4417 | 3356-3363 | 8mer | -0.18 | 89 | -0.18 | 0 | N/A |
| hsa-miR-4296 | 3357-3363 | 7mer-m8 | -0.24 | 91 | -0.24 | 0 | N/A |
| hsa-miR-4265 | 3357-3363 | 7mer-m8 | -0.24 | 91 | -0.24 | 0 | N/A |
| hsa-miR-4322 | 3357-3363 | 7mer-m8 | -0.21 | 90 | -0.21 | 0 | N/A |
| hsa-miR-299-3p | 3358-3364 | 7mer-m8 | -0.08 | 66 | -0.08 | 0.117 | N/A |
| hsa-miR-576-3p | 3365-3371 | 7mer-m8 | -0.08 | 70 | -0.08 | 0.072 | N/A |
| hsa-miR-6888-5p | 3368-3374 | 7mer-m8 | -0.02 | 42 | -0.02 | 0 | N/A |
| hsa-miR-4768-3p | 3369-3375 | 7mer-m8 | -0.02 | 38 | -0.02 | 0 | N/A |
| hsa-miR-665 | 3370-3376 | 7mer-m8 | -0.07 | 64 | -0.07 | 0.311 | N/A |
| hsa-miR-1307-3p | 3376-3382 | 7mer-1A | -0.1 | 65 | -0.1 | 0.021 | N/A |
| hsa-miR-4638-5p | 3376-3382 | 7mer-1A | -0.12 | 62 | -0.12 | 0 | N/A |
| hsa-miR-3615 | 3377-3383 | 7mer-m8 | -0.06 | 33 | -0.06 | 0 | N/A |
| hsa-miR-6762-3p | 3382-3389 | 8mer | -0.1 | 68 | -0.1 | 0 | N/A |
| hsa-miR-604 | 3383-3389 | 7mer-1A | -0.11 | 75 | -0.11 | 0.021 | N/A |
| hsa-miR-647 | 3383-3389 | 7mer-m8 | -0.02 | 58 | -0.02 | 0 | N/A |
| hsa-miR-5693 | 3386-3392 | 7mer-m8 | -0.02 | 53 | -0.02 | 0 | N/A |
| hsa-miR-34a-5p | 3388-3395 | 8mer | -0.04 | 32 | -0.04 | 0.201 | < 0.1 |
| hsa-miR-34c-5p | 3388-3395 | 8mer | -0.04 | 32 | -0.04 | 0.201 | < 0.1 |
| hsa-miR-449b-5p | 3388-3395 | 8mer | -0.03 | 29 | -0.03 | 0.201 | < 0.1 |
| hsa-miR-449a | 3388-3395 | 8mer | -0.03 | 27 | -0.03 | 0.201 | < 0.1 |
| hsa-miR-548au-3p | 3389-3395 | 7mer-1A | -0.01 | 17 | -0.01 | 0 | N/A |
| hsa-miR-4481 | 3393-3399 | 7mer-1A | -0.05 | 55 | -0.05 | 0 | N/A |
| hsa-miR-4758-5p | 3393-3399 | 7mer-m8 | -0.02 | 48 | -0.02 | 0 | N/A |
| hsa-miR-1238-5p | 3393-3399 | 7mer-m8 | -0.02 | 47 | -0.02 | 0 | N/A |
| hsa-miR-4745-5p | 3393-3399 | 7mer-1A | -0.03 | 32 | -0.03 | 0 | N/A |
| hsa-miR-6857-3p | 3396-3402 | 7mer-m8 | -0.04 | 44 | -0.04 | 0 | N/A |
| hsa-miR-4499 | 3397-3403 | 7mer-m8 | -0.02 | 27 | -0.02 | 0 | N/A |
| hsa-miR-4494 | 3398-3405 | 8mer | -0.04 | 54 | -0.04 | 0 | N/A |
| hsa-miR-499b-5p | 3399-3405 | 7mer-1A | -0.01 | 26 | -0.01 | 0 | N/A |
| hsa-miR-455-3p.2 | 3414-3420 | 7mer-m8 | -0.08 | 85 | -0.08 | 0.903 | < 0.1 |
| hsa-miR-5683 | 3419-3426 | 8mer | -0.03 | 56 | -0.03 | 0 | N/A |
| hsa-miR-345-3p | 3429-3435 | 7mer-m8 | -0.02 | 39 | -0.02 | 0 | N/A |
| hsa-miR-3130-3p | 3435-3441 | 7mer-m8 | -0.02 | 61 | -0.02 | 0 | N/A |
| hsa-miR-6890-3p | 3438-3444 | 7mer-1A | -0.01 | 43 | -0.01 | 0 | N/A |
| hsa-miR-1304-3p | 3439-3445 | 7mer-m8 | -0.02 | 38 | -0.02 | 0 | N/A |
| hsa-miR-3670 | 3442-3449 | 8mer | -0.03 | 22 | -0.03 | 0 | N/A |
| hsa-miR-4792 | 3445-3451 | 7mer-m8 | -0.16 | 85 | -0.16 | 0 | N/A |
| hsa-miR-7854-3p | 3447-3453 | 7mer-m8 | -0.02 | 20 | -0.02 | 0 | N/A |
| hsa-miR-6731-5p | 3451-3458 | 8mer | -0.03 | 38 | -0.03 | 0 | N/A |
| hsa-miR-8085 | 3451-3458 | 8mer | -0.03 | 37 | -0.03 | 0 | N/A |
| hsa-miR-6878-5p | 3452-3458 | 7mer-1A | -0.09 | 61 | -0.09 | 0 | N/A |
| hsa-miR-30c-1-3p | 3452-3458 | 7mer-m8 | -0.02 | 37 | -0.02 | 0 | N/A |
| hsa-miR-30c-2-3p | 3452-3458 | 7mer-m8 | -0.02 | 34 | -0.02 | 0 | N/A |
| hsa-miR-6788-5p | 3452-3458 | 7mer-m8 | -0.02 | 32 | -0.02 | 0 | N/A |
| hsa-miR-6778-5p | 3453-3459 | 7mer-m8 | -0.13 | 62 | -0.13 | 0.409 | N/A |
| hsa-miR-1233-5p | 3453-3459 | 7mer-m8 | -0.11 | 57 | -0.11 | 0.409 | N/A |
| hsa-miR-194-3p | 3455-3461 | 7mer-m8 | -0.05 | 68 | -0.05 | 0 | N/A |
| hsa-miR-6776-5p | 3455-3467 | non-canonical | N/A | N/A | N/A | 0 | N/A |
| hsa-miR-6776-5p | 3455-3467 | non-canonical | N/A | N/A | N/A | 0 | N/A |
| hsa-miR-6499-3p | 3457-3463 | 7mer-1A | -0.01 | 23 | -0.01 | 0 | N/A |
| hsa-miR-143-5p | 3458-3464 | 7mer-m8 | -0.02 | 42 | -0.02 | 0 | N/A |
| hsa-miR-6764-5p | 3464-3470 | 7mer-m8 | -0.03 | 58 | -0.03 | 0 | N/A |
| hsa-miR-1915-3p | 3464-3470 | 7mer-m8 | -0.02 | 53 | -0.02 | 0 | N/A |
| hsa-miR-7974 | 3478-3484 | 7mer-m8 | -0.02 | 20 | -0.02 | 0 | N/A |
| hsa-miR-4675 | 3479-3485 | 7mer-m8 | -0.02 | 49 | -0.02 | 0 | N/A |
| hsa-miR-4741 | 3479-3485 | 7mer-m8 | -0.02 | 33 | -0.02 | 0 | N/A |
| hsa-miR-4446-3p | 3480-3486 | 7mer-m8 | -0.1 | 67 | -0.1 | 0.075 | N/A |
| hsa-miR-4721 | 3481-3488 | 8mer | -0.16 | 79 | -0.16 | 0 | N/A |
| hsa-miR-1226-5p | 3482-3488 | 7mer-m8 | -0.14 | 85 | -0.14 | 0 | N/A |
| hsa-miR-3616-3p | 3482-3488 | 7mer-1A | -0.15 | 70 | -0.15 | 0 | N/A |
| hsa-miR-186-5p | 3488-3494 | 7mer-m8 | -0.02 | 73 | -0.02 | 0.587 | N/A |
| hsa-miR-8060 | 3494-3500 | 7mer-1A | -0.02 | 57 | -0.02 | 0 | N/A |
| hsa-miR-7154-5p | 3494-3500 | 7mer-m8 | -0.02 | 28 | -0.02 | 0 | N/A |
| hsa-miR-6847-3p | 3496-3502 | 7mer-m8 | -0.04 | 63 | -0.04 | 0 | N/A |
| hsa-miR-4437 | 3498-3504 | 7mer-m8 | -0.14 | 69 | -0.14 | 0 | N/A |
| hsa-miR-3944-3p | 3500-3506 | 7mer-m8 | -0.21 | 69 | -0.21 | 0 | N/A |
| hsa-miR-6856-5p | 3507-3513 | 7mer-m8 | -0.02 | 44 | -0.02 | 0 | N/A |
| hsa-miR-6758-5p | 3507-3513 | 7mer-m8 | -0.02 | 44 | -0.02 | 0 | N/A |
| hsa-miR-127-5p | 3520-3526 | 7mer-m8 | -0.02 | 35 | -0.02 | 0 | N/A |
| hsa-miR-3928-5p | 3520-3526 | 7mer-1A | -0.01 | 34 | -0.01 | 0 | N/A |
| hsa-miR-6806-3p | 3520-3526 | 7mer-1A | -0.01 | 33 | -0.01 | 0 | N/A |
| hsa-miR-2467-3p | 3529-3535 | 7mer-m8 | -0.02 | 33 | -0.02 | 0 | N/A |
| hsa-miR-6868-5p | 3532-3538 | 7mer-m8 | -0.16 | 84 | -0.16 | 0 | N/A |
| hsa-miR-6757-3p | 3535-3541 | 7mer-m8 | -0.02 | 42 | -0.02 | 0 | N/A |
| hsa-miR-3191-3p | 3540-3546 | 7mer-1A | -0.15 | 75 | -0.15 | 0 | N/A |
| hsa-miR-6810-5p | 3540-3546 | 7mer-m8 | -0.07 | 68 | -0.07 | 0 | N/A |
| hsa-miR-4652-5p | 3540-3546 | 7mer-1A | -0.1 | 59 | -0.1 | 0 | N/A |
| hsa-miR-3144-5p | 3540-3546 | 7mer-1A | -0.09 | 57 | -0.09 | 0 | N/A |
| hsa-miR-4700-5p | 3541-3547 | 7mer-m8 | -0.03 | 31 | -0.03 | 0.075 | N/A |
| hsa-miR-8089 | 3541-3547 | 7mer-m8 | -0.02 | 24 | -0.02 | 0.075 | N/A |
| hsa-miR-4667-5p | 3541-3547 | 7mer-m8 | -0.02 | 20 | -0.02 | 0.075 | N/A |
| hsa-miR-4731-5p | 3542-3549 | 8mer | -0.14 | 81 | -0.14 | 0 | N/A |
| hsa-miR-5589-5p | 3543-3549 | 7mer-1A | -0.01 | 37 | -0.01 | 0 | N/A |
| hsa-miR-512-3p | 3545-3551 | 7mer-m8 | -0.03 | 54 | -0.03 | 0.075 | N/A |
| hsa-miR-373-3p | 3546-3552 | 7mer-m8 | -0.02 | 36 | -0.02 | 0.596 | < 0.1 |
| hsa-miR-302d-3p | 3546-3552 | 7mer-m8 | -0.02 | 29 | -0.02 | 0.596 | < 0.1 |
| hsa-miR-302b-3p | 3546-3552 | 7mer-m8 | -0.02 | 29 | -0.02 | 0.596 | < 0.1 |
| hsa-miR-302e | 3546-3552 | 7mer-m8 | -0.02 | 29 | -0.02 | 0.596 | < 0.1 |
| hsa-miR-302c-3p.1 | 3546-3552 | 7mer-m8 | -0.02 | 29 | -0.02 | 0.596 | < 0.1 |
| hsa-miR-302a-3p | 3546-3552 | 7mer-m8 | -0.02 | 29 | -0.02 | 0.596 | < 0.1 |
| hsa-miR-520d-3p | 3546-3552 | 7mer-m8 | -0.02 | 28 | -0.02 | 0.596 | < 0.1 |
| hsa-miR-520e | 3546-3552 | 7mer-m8 | -0.02 | 28 | -0.02 | 0.596 | < 0.1 |
| hsa-miR-372-3p | 3546-3552 | 7mer-m8 | -0.02 | 27 | -0.02 | 0.596 | < 0.1 |
| hsa-miR-520a-3p | 3546-3552 | 7mer-m8 | -0.02 | 27 | -0.02 | 0.596 | < 0.1 |
| hsa-miR-520b | 3546-3552 | 7mer-m8 | -0.02 | 27 | -0.02 | 0.596 | < 0.1 |
| hsa-miR-520c-3p | 3546-3552 | 7mer-m8 | -0.02 | 27 | -0.02 | 0.596 | < 0.1 |
| hsa-miR-526b-3p | 3547-3553 | 7mer-m8 | -0.02 | 62 | -0.02 | 0.949 | < 0.1 |
| hsa-miR-93-5p | 3547-3553 | 7mer-m8 | -0.02 | 59 | -0.02 | 0.949 | < 0.1 |
| hsa-miR-20a-5p | 3547-3553 | 7mer-m8 | -0.02 | 58 | -0.02 | 0.949 | < 0.1 |
| hsa-miR-20b-5p | 3547-3553 | 7mer-m8 | -0.02 | 58 | -0.02 | 0.949 | < 0.1 |
| hsa-miR-17-5p | 3547-3553 | 7mer-m8 | -0.02 | 58 | -0.02 | 0.949 | < 0.1 |
| hsa-miR-106b-5p | 3547-3553 | 7mer-m8 | -0.02 | 58 | -0.02 | 0.949 | < 0.1 |
| hsa-miR-519d-3p | 3547-3553 | 7mer-m8 | -0.02 | 57 | -0.02 | 0.949 | < 0.1 |
| hsa-miR-106a-5p | 3547-3553 | 7mer-m8 | -0.02 | 56 | -0.02 | 0.949 | < 0.1 |
| hsa-miR-548az-5p | 3548-3555 | 8mer | -0.03 | 78 | -0.03 | 0 | N/A |
| hsa-miR-548t-5p | 3548-3555 | 8mer | -0.03 | 78 | -0.03 | 0 | N/A |
| hsa-miR-548n | 3549-3555 | 7mer-1A | -0.01 | 41 | -0.01 | 0.075 | N/A |
| hsa-miR-192-5p | 3554-3560 | 7mer-m8 | -0.12 | 81 | -0.12 | 0.574 | < 0.1 |
| hsa-miR-215-5p | 3554-3560 | 7mer-m8 | -0.12 | 81 | -0.12 | 0.574 | < 0.1 |
| hsa-miR-6513-3p | 3566-3572 | 7mer-m8 | -0.02 | 47 | -0.02 | 0 | N/A |
| hsa-miR-6793-3p | 3570-3576 | 7mer-1A | -0.01 | 39 | -0.01 | 0 | N/A |
| hsa-miR-2355-5p | 3571-3577 | 7mer-1A | -0.1 | 73 | -0.1 | 0 | N/A |
| hsa-miR-3679-3p | 3572-3578 | 7mer-1A | -0.01 | 35 | -0.01 | 0 | N/A |
| hsa-miR-4446-5p | 3573-3579 | 7mer-1A | -0.01 | 28 | -0.01 | 0 | N/A |
| hsa-miR-5003-3p | 3575-3581 | 7mer-m8 | -0.02 | 69 | -0.02 | 0 | N/A |
| hsa-miR-6878-3p | 3582-3588 | 7mer-m8 | -0.17 | 84 | -0.17 | 0 | N/A |
| hsa-miR-4757-5p | 3582-3588 | 7mer-1A | -0.11 | 77 | -0.11 | 0.072 | N/A |
| hsa-miR-6744-3p | 3582-3588 | 7mer-1A | -0.05 | 74 | -0.05 | 0.072 | N/A |
| hsa-miR-3922-3p | 3583-3589 | 7mer-m8 | -0.13 | 85 | -0.13 | 0 | N/A |
| hsa-miR-3176 | 3583-3589 | 7mer-m8 | -0.11 | 82 | -0.11 | 0 | N/A |
| hsa-miR-193b-3p | 3584-3590 | 7mer-m8 | -0.25 | 89 | -0.25 | 0.917 | < 0.1 |
| hsa-miR-193a-3p | 3584-3590 | 7mer-m8 | -0.25 | 89 | -0.25 | 0.917 | < 0.1 |
| hsa-miR-4463 | 3586-3592 | 7mer-m8 | -0.02 | 55 | -0.02 | 0 | N/A |
| hsa-miR-4494 | 3587-3593 | 7mer-m8 | -0.02 | 35 | -0.02 | 0 | N/A |
| hsa-miR-4771 | 3588-3594 | 7mer-m8 | -0.02 | 20 | -0.02 | 0 | N/A |
| hsa-miR-7112-5p | 3591-3597 | 7mer-m8 | -0.21 | 71 | -0.21 | 0 | N/A |
| hsa-miR-329-5p | 3607-3613 | 7mer-m8 | -0.02 | 51 | -0.02 | 0.072 | N/A |
| hsa-miR-655-5p | 3609-3615 | 7mer-1A | -0.13 | 77 | -0.13 | 0 | N/A |
| hsa-miR-6086 | 3609-3615 | 7mer-1A | -0.04 | 67 | -0.04 | 0.310 | N/A |
| hsa-miR-377-5p | 3609-3615 | 7mer-1A | -0.08 | 65 | -0.08 | 0.310 | N/A |
| hsa-miR-4666a-3p | 3622-3629 | 8mer | -0.03 | 75 | -0.03 | 0 | N/A |
| hsa-miR-300 | 3623-3629 | 7mer-1A | -0.01 | 51 | -0.01 | 0.179 | N/A |
| hsa-miR-381-3p | 3623-3629 | 7mer-1A | -0.01 | 48 | -0.01 | 0.179 | N/A |
| hsa-let-7b-3p | 3623-3629 | 7mer-m8 | -0.02 | 36 | -0.02 | 0.072 | N/A |
| hsa-let-7a-3p | 3623-3629 | 7mer-m8 | -0.02 | 35 | -0.02 | 0.072 | N/A |
| hsa-miR-98-3p | 3623-3629 | 7mer-m8 | -0.02 | 35 | -0.02 | 0.072 | N/A |
| hsa-let-7f-1-3p | 3623-3629 | 7mer-m8 | -0.02 | 35 | -0.02 | 0.072 | N/A |
| hsa-miR-6505-5p | 3628-3635 | 8mer | -0.14 | 88 | -0.14 | 0.072 | N/A |
| hsa-miR-9-5p | 3632-3638 | 7mer-1A | -0.08 | 80 | -0.08 | 2.586 | < 0.1 |
| hsa-miR-4539 | 3641-3647 | 7mer-1A | -0.01 | 41 | -0.01 | 0 | N/A |
| hsa-miR-4762-3p | 3643-3649 | 7mer-1A | -0.06 | 77 | -0.06 | 0 | N/A |
| hsa-miR-513a-3p | 3648-3654 | 7mer-1A | -0.01 | 57 | -0.01 | 0 | N/A |
| hsa-miR-513c-3p | 3648-3654 | 7mer-1A | -0.01 | 57 | -0.01 | 0 | N/A |
| hsa-miR-3606-3p | 3648-3654 | 7mer-1A | -0.01 | 57 | -0.01 | 0 | N/A |
| hsa-miR-195-3p | 3654-3660 | 7mer-m8 | -0.02 | 41 | -0.02 | 0 | N/A |
| hsa-miR-16-2-3p | 3654-3660 | 7mer-m8 | -0.02 | 40 | -0.02 | 0 | N/A |
| hsa-miR-211-3p | 3661-3667 | 7mer-m8 | -0.08 | 86 | -0.08 | 0 | N/A |
| hsa-miR-7113-5p | 3662-3668 | 7mer-m8 | -0.12 | 78 | -0.12 | 0 | N/A |
| hsa-miR-6753-5p | 3663-3669 | 7mer-m8 | -0.19 | 86 | -0.19 | 0 | N/A |
| hsa-miR-4733-3p | 3664-3670 | 7mer-m8 | -0.13 | 82 | -0.13 | 0.409 | N/A |
| hsa-miR-3692-3p | 3668-3674 | 7mer-1A | -0.04 | 62 | -0.04 | 0 | N/A |
| hsa-miR-4452 | 3673-3679 | 7mer-1A | -0.01 | 46 | -0.01 | 0 | N/A |
| hsa-miR-183-3p | 3673-3679 | 7mer-1A | -0.01 | 43 | -0.01 | 0 | N/A |
| hsa-miR-4276 | 3685-3692 | 8mer | -0.17 | 85 | -0.17 | 0 | N/A |
| hsa-miR-5190 | 3685-3691 | 7mer-1A | -0.01 | 53 | -0.01 | 0 | N/A |
| hsa-miR-22-5p | 3690-3697 | 8mer | -0.07 | 69 | -0.07 | 0 | N/A |
| hsa-miR-4677-5p | 3691-3697 | 7mer-m8 | -0.02 | 45 | -0.02 | 0.072 | N/A |
| hsa-miR-2116-5p | 3691-3697 | 7mer-1A | -0.01 | 25 | -0.01 | 0 | N/A |
| hsa-miR-26b-3p | 3692-3698 | 7mer-m8 | -0.07 | 67 | -0.07 | 0.072 | N/A |
| hsa-miR-224-5p | 3700-3706 | 7mer-1A | -0.02 | 60 | -0.02 | 1.430 | N/A |
| hsa-miR-7856-5p | 3703-3709 | 7mer-1A | -0.01 | 40 | -0.01 | 0 | N/A |
| hsa-miR-590-3p | 3705-3711 | 7mer-m8 | -0.02 | 59 | -0.02 | 0.075 | N/A |
| hsa-miR-133a-3p.2 | 3712-3718 | 7mer-m8 | -0.27 | 90 | -0.27 | 1.223 | < 0.1 |
| hsa-miR-133b | 3712-3718 | 7mer-m8 | -0.27 | 90 | -0.27 | 1.223 | < 0.1 |
| hsa-miR-133a-3p.1 | 3712-3718 | 7mer-1A | -0.15 | 71 | -0.15 | 1.223 | < 0.1 |
| hsa-miR-7109-3p | 3725-3732 | 8mer | -0.3 | 97 | -0.3 | 0 | N/A |
| hsa-miR-4503 | 3728-3734 | 7mer-1A | -0.09 | 64 | -0.09 | 0 | N/A |
| hsa-miR-7856-5p | 3729-3735 | 7mer-1A | -0.01 | 40 | -0.01 | 0 | N/A |
| hsa-miR-6832-3p | 3732-3739 | 8mer | -0.07 | 72 | -0.07 | 0 | N/A |
| hsa-miR-204-5p | 3733-3739 | 7mer-m8 | -0.03 | 57 | -0.03 | 0.349 | < 0.1 |
| hsa-miR-211-5p | 3733-3739 | 7mer-m8 | -0.03 | 57 | -0.03 | 0.349 | < 0.1 |
| hsa-miR-4281 | 3736-3742 | 7mer-1A | -0.15 | 89 | -0.15 | 0 | N/A |
| hsa-miR-133a-3p.1 | 3736-3742 | 7mer-m8 | -0.25 | 86 | -0.25 | 0.192 | < 0.1 |
| hsa-miR-7152-3p | 3737-3743 | 7mer-m8 | -0.17 | 85 | -0.17 | 0 | N/A |
| hsa-miR-504-5p.1 | 3740-3746 | 7mer-m8 | -0.14 | 89 | -0.14 | 0.190 | N/A |
| hsa-miR-6732-3p | 3741-3747 | 7mer-m8 | -0.16 | 84 | -0.16 | 0 | N/A |
| hsa-miR-548as-3p | 3743-3749 | 7mer-m8 | -0.05 | 80 | -0.05 | 0 | N/A |
| hsa-miR-4668-5p | 3750-3761 | non-canonical | N/A | N/A | N/A | 0 | N/A |
| hsa-miR-4668-5p | 3750-3761 | non-canonical | N/A | N/A | N/A | 0 | N/A |
| hsa-miR-1285-3p | 3795-3801 | 7mer-m8 | -0.11 | 75 | -0.11 | 0.021 | N/A |
| hsa-miR-5189-5p | 3795-3801 | 7mer-m8 | -0.09 | 72 | -0.09 | 0.021 | N/A |
| hsa-miR-612 | 3795-3801 | 7mer-m8 | -0.02 | 57 | -0.02 | 0.021 | N/A |
| hsa-miR-3187-5p | 3795-3801 | 7mer-m8 | -0.05 | 57 | -0.05 | 0.021 | N/A |
| hsa-miR-6860 | 3795-3801 | 7mer-m8 | -0.03 | 43 | -0.03 | 0.021 | N/A |
| hsa-miR-939-3p | 3796-3802 | 7mer-m8 | -0.16 | 86 | -0.16 | 0 | N/A |
| hsa-miR-661 | 3797-3803 | 7mer-m8 | -0.14 | 79 | -0.14 | 0.021 | N/A |
| hsa-miR-6849-3p | 3800-3807 | 8mer | -0.03 | 48 | -0.03 | 0 | N/A |
| hsa-miR-6512-3p | 3801-3807 | 7mer-m8 | -0.05 | 57 | -0.05 | 0 | N/A |
| hsa-miR-6720-5p | 3801-3807 | 7mer-m8 | -0.04 | 52 | -0.04 | 0 | N/A |
| hsa-miR-766-3p | 3802-3808 | 7mer-m8 | -0.02 | 43 | -0.02 | 0.021 | N/A |
| hsa-miR-508-5p | 3803-3809 | 7mer-m8 | -0.02 | 33 | -0.02 | 0.072 | N/A |
| hsa-miR-7703 | 3806-3812 | 7mer-m8 | -0.02 | 41 | -0.02 | 0 | N/A |
| hsa-miR-4793-3p | 3807-3813 | 7mer-m8 | -0.02 | 36 | -0.02 | 0 | N/A |
| hsa-miR-1273g-3p | 3810-3816 | 7mer-m8 | -0.16 | 86 | -0.16 | 0 | N/A |
| hsa-miR-4252 | 3811-3817 | 7mer-m8 | -0.02 | 29 | -0.02 | 0 | N/A |
| hsa-miR-566 | 3815-3821 | 7mer-1A | -0.13 | 68 | -0.13 | 0.021 | N/A |
| hsa-miR-6789-3p | 3815-3821 | 7mer-1A | -0.17 | 65 | -0.17 | 0 | N/A |
| hsa-miR-652-3p | 3816-3822 | 7mer-m8 | -0.18 | 77 | -0.18 | 0.042 | N/A |
| hsa-miR-4647 | 3819-3825 | 7mer-m8 | -0.02 | 60 | -0.02 | 0 | N/A |
| hsa-miR-4426 | 3819-3825 | 7mer-m8 | -0.02 | 59 | -0.02 | 0 | N/A |
| hsa-miR-4662b | 3819-3825 | 7mer-m8 | -0.02 | 44 | -0.02 | 0 | N/A |
| hsa-miR-6807-5p | 3825-3831 | 7mer-m8 | -0.02 | 32 | -0.02 | 0 | N/A |
| hsa-miR-6499-3p | 3830-3836 | 7mer-1A | -0.01 | 23 | -0.01 | 0 | N/A |
| hsa-miR-455-3p.2 | 3831-3837 | 7mer-1A | -0.01 | 46 | -0.01 | 0.193 | < 0.1 |
| hsa-miR-6516-5p | 3831-3837 | 7mer-m8 | -0.02 | 34 | -0.02 | 0 | N/A |
| hsa-miR-6086 | 3835-3841 | 7mer-m8 | -0.02 | 35 | -0.02 | 0.072 | N/A |
| hsa-miR-377-5p | 3835-3841 | 7mer-m8 | -0.02 | 22 | -0.02 | 0.072 | N/A |
| hsa-miR-4257 | 3837-3843 | 7mer-m8 | -0.02 | 48 | -0.02 | 0 | N/A |
| hsa-miR-2467-3p | 3838-3844 | 7mer-m8 | -0.04 | 49 | -0.04 | 0 | N/A |
| hsa-miR-1910-3p | 3840-3846 | 7mer-m8 | -0.02 | 49 | -0.02 | 0 | N/A |
| hsa-miR-6511a-5p | 3840-3846 | 7mer-m8 | -0.02 | 38 | -0.02 | 0 | N/A |
| hsa-miR-1827 | 3841-3847 | 7mer-m8 | -0.05 | 48 | -0.05 | 0.021 | N/A |
| hsa-miR-650 | 3842-3848 | 7mer-m8 | -0.06 | 49 | -0.06 | 0.075 | N/A |
| hsa-miR-3612 | 3842-3848 | 7mer-m8 | -0.06 | 44 | -0.06 | 0.075 | N/A |
| hsa-miR-6799-5p | 3844-3850 | 7mer-1A | -0.03 | 66 | -0.03 | 0 | N/A |
| hsa-miR-6779-5p | 3844-3850 | 7mer-m8 | -0.05 | 51 | -0.05 | 0.072 | N/A |
| hsa-miR-30b-3p | 3844-3850 | 7mer-m8 | -0.04 | 49 | -0.04 | 0.072 | N/A |
| hsa-miR-1273h-5p | 3844-3850 | 7mer-m8 | -0.05 | 49 | -0.05 | 0.072 | N/A |
| hsa-miR-7106-5p | 3844-3850 | 7mer-1A | -0.04 | 46 | -0.04 | 0 | N/A |
| hsa-miR-6883-5p | 3844-3850 | 7mer-1A | -0.03 | 44 | -0.03 | 0 | N/A |
| hsa-miR-6780a-5p | 3844-3850 | 7mer-m8 | -0.03 | 43 | -0.03 | 0.072 | N/A |
| hsa-miR-4728-5p | 3844-3850 | 7mer-1A | -0.03 | 41 | -0.03 | 0 | N/A |
| hsa-miR-149-3p | 3844-3850 | 7mer-1A | -0.02 | 34 | -0.02 | 0 | N/A |
| hsa-miR-3689a-3p | 3844-3850 | 7mer-m8 | -0.02 | 30 | -0.02 | 0.072 | N/A |
| hsa-miR-3689c | 3844-3850 | 7mer-m8 | -0.02 | 29 | -0.02 | 0.072 | N/A |
| hsa-miR-3689b-3p | 3844-3850 | 7mer-m8 | -0.02 | 29 | -0.02 | 0.072 | N/A |
| hsa-miR-6785-5p | 3844-3850 | 7mer-1A | -0.01 | 25 | -0.01 | 0 | N/A |
| hsa-miR-3192-5p | 3845-3851 | 7mer-m8 | -0.05 | 67 | -0.05 | 0.021 | N/A |
| hsa-miR-7851-3p | 3847-3853 | 7mer-m8 | -0.21 | 86 | -0.21 | 0 | N/A |
| hsa-miR-490-3p | 3848-3854 | 7mer-m8 | -0.02 | 37 | -0.02 | 0.109 | < 0.1 |
| hsa-miR-4768-3p | 3863-3869 | 7mer-m8 | -0.02 | 38 | -0.02 | 0 | N/A |
| hsa-miR-4722-5p | 3864-3870 | 7mer-m8 | -0.03 | 48 | -0.03 | 0 | N/A |
| hsa-miR-940 | 3866-3872 | 7mer-m8 | -0.13 | 85 | -0.13 | 0.021 | N/A |
| hsa-miR-6893-5p | 3866-3872 | 7mer-m8 | -0.08 | 74 | -0.08 | 0.021 | N/A |
| hsa-miR-6808-5p | 3866-3872 | 7mer-m8 | -0.07 | 71 | -0.07 | 0.021 | N/A |
| hsa-miR-1827 | 3867-3874 | 8mer | -0.04 | 45 | -0.04 | 0.021 | N/A |
| hsa-miR-4649-3p | 3869-3875 | 7mer-m8 | -0.04 | 68 | -0.04 | 0 | N/A |
| hsa-miR-7160-5p | 3870-3876 | 7mer-m8 | -0.03 | 44 | -0.03 | 0 | N/A |
| hsa-miR-665 | 3870-3882 | non-canonical | N/A | N/A | N/A | 0 | N/A |
| hsa-miR-665 | 3870-3882 | non-canonical | N/A | N/A | N/A | 0 | N/A |
| hsa-miR-4419b | 3872-3878 | 7mer-m8 | -0.02 | 54 | -0.02 | 0 | N/A |
| hsa-miR-4478 | 3872-3878 | 7mer-m8 | -0.02 | 54 | -0.02 | 0 | N/A |
| hsa-miR-3929 | 3872-3878 | 7mer-m8 | -0.02 | 54 | -0.02 | 0 | N/A |
| hsa-miR-6884-5p | 3873-3879 | 7mer-m8 | -0.02 | 27 | -0.02 | 0.179 | N/A |
| hsa-miR-485-5p | 3873-3879 | 7mer-m8 | -0.02 | 18 | -0.02 | 0.179 | N/A |
| hsa-miR-4695-5p | 3875-3881 | 7mer-m8 | -0.1 | 75 | -0.1 | 0 | N/A |
| hsa-miR-4459 | 3876-3883 | 8mer | -0.12 | 86 | -0.12 | 0 | N/A |
| hsa-miR-4433a-3p | 3877-3883 | 7mer-1A | -0.01 | 39 | -0.01 | 0.021 | N/A |
| hsa-miR-4768-3p | 3877-3883 | 7mer-1A | -0.01 | 33 | -0.01 | 0.072 | N/A |
| hsa-miR-3664-3p | 3878-3884 | 7mer-m8 | -0.02 | 33 | -0.02 | 0 | N/A |
| hsa-miR-510-5p | 3879-3886 | 8mer | -0.03 | 47 | -0.03 | 0 | N/A |
| hsa-miR-512-5p | 3880-3886 | 7mer-1A | -0.01 | 35 | -0.01 | 0.072 | N/A |
| hsa-miR-5690 | 3884-3890 | 7mer-m8 | -0.02 | 43 | -0.02 | 0 | N/A |
| hsa-miR-7977 | 3887-3893 | 7mer-m8 | -0.02 | 47 | -0.02 | 0 | N/A |
| hsa-miR-4281 | 3890-3896 | 7mer-1A | -0.06 | 65 | -0.06 | 0 | N/A |
| hsa-miR-133a-3p.1 | 3890-3896 | 7mer-m8 | -0.08 | 57 | -0.08 | 0.021 | < 0.1 |
| hsa-miR-6811-3p | 3895-3901 | 7mer-m8 | -0.02 | 31 | -0.02 | 0 | N/A |
| hsa-miR-6078 | 3897-3903 | 7mer-1A | -0.2 | 89 | -0.2 | 0 | N/A |
| hsa-miR-449b-5p | 3903-3909 | 7mer-1A | -0.05 | 38 | -0.05 | 0.052 | < 0.1 |
| hsa-miR-449a | 3903-3909 | 7mer-1A | -0.03 | 24 | -0.03 | 0.052 | < 0.1 |
| hsa-miR-34a-5p | 3903-3909 | 7mer-1A | -0.03 | 23 | -0.03 | 0.052 | < 0.1 |
| hsa-miR-548au-3p | 3903-3909 | 7mer-1A | -0.01 | 17 | -0.01 | 0 | N/A |
| hsa-miR-34c-5p | 3903-3909 | 7mer-1A | -0.01 | 14 | -0.01 | 0.052 | < 0.1 |
| hsa-miR-4455 | 3911-3918 | 8mer | -0.16 | 67 | -0.16 | 0 | N/A |
| hsa-miR-6772-5p | 3912-3918 | 7mer-1A | -0.12 | 67 | -0.12 | 0 | N/A |
| hsa-miR-609 | 3912-3918 | 7mer-1A | -0.06 | 49 | -0.06 | 0.021 | N/A |
| hsa-miR-6776-5p | 3913-3919 | 7mer-m8 | -0.13 | 79 | -0.13 | 0 | N/A |
| hsa-miR-5589-5p | 3914-3920 | 7mer-m8 | -0.1 | 89 | -0.1 | 0 | N/A |
| hsa-miR-3613-3p | 3924-3930 | 7mer-m8 | -0.02 | 36 | -0.02 | 0 | N/A |
| hsa-miR-548c-3p | 3932-3938 | 7mer-1A | -0.01 | 57 | -0.01 | 0.021 | N/A |
| hsa-miR-4684-5p | 3939-3945 | 7mer-m8 | -0.08 | 73 | -0.08 | 0 | N/A |
| hsa-miR-593-3p | 3941-3947 | 7mer-1A | -0.05 | 57 | -0.05 | 0.075 | N/A |
| hsa-miR-6818-3p | 3941-3947 | 7mer-m8 | -0.02 | 38 | -0.02 | 0 | N/A |
| hsa-miR-6895-3p | 3941-3947 | 7mer-1A | -0.01 | 32 | -0.01 | 0 | N/A |
| hsa-miR-4421 | 3943-3949 | 7mer-m8 | -0.09 | 67 | -0.09 | 0 | N/A |
| hsa-miR-5699-3p | 3943-3949 | 7mer-m8 | -0.07 | 63 | -0.07 | 0 | N/A |
| hsa-miR-339-5p | 3944-3950 | 7mer-m8 | -0.15 | 79 | -0.15 | 0.556 | N/A |
| hsa-miR-10b-5p | 3945-3951 | 7mer-m8 | -0.09 | 78 | -0.09 | 0.556 | < 0.1 |
| hsa-miR-10a-5p | 3945-3951 | 7mer-m8 | -0.09 | 77 | -0.09 | 0.556 | < 0.1 |
| hsa-miR-6732-3p | 3946-3952 | 7mer-m8 | -0.14 | 80 | -0.14 | 0 | N/A |
| hsa-miR-3926 | 3961-3967 | 7mer-1A | -0.09 | 67 | -0.09 | 0 | N/A |
| hsa-miR-548s | 3961-3967 | 7mer-m8 | -0.02 | 25 | -0.02 | 0 | N/A |
| hsa-miR-3190-5p | 3962-3968 | 7mer-m8 | -0.08 | 65 | -0.08 | 0 | N/A |
| hsa-miR-6849-3p | 3967-3973 | 7mer-m8 | -0.02 | 29 | -0.02 | 0 | N/A |
| hsa-miR-4786-5p | 3971-3977 | 7mer-m8 | -0.02 | 16 | -0.02 | 0 | N/A |
| hsa-miR-4433a-3p | 3980-3987 | 8mer | -0.03 | 66 | -0.03 | 0.021 | N/A |
| hsa-miR-4768-3p | 3981-3987 | 7mer-1A | -0.12 | 86 | -0.12 | 0.072 | N/A |
| hsa-miR-4459 | 3981-3987 | 7mer-1A | -0.1 | 82 | -0.1 | 0 | N/A |
| hsa-miR-3165 | 3997-4003 | 7mer-m8 | -0.05 | 52 | -0.05 | 0.021 | N/A |
| hsa-miR-6745 | 3998-4005 | 8mer | -0.18 | 84 | -0.18 | 0 | N/A |
| hsa-miR-363-5p | 3998-4005 | 8mer | -0.18 | 83 | -0.18 | 0 | N/A |
| hsa-miR-4483 | 3999-4005 | 7mer-1A | -0.11 | 86 | -0.11 | 0.021 | N/A |
| hsa-miR-6756-5p | 3999-4005 | 7mer-1A | -0.25 | 84 | -0.25 | 0 | N/A |
| hsa-miR-1293 | 3999-4005 | 7mer-1A | -0.12 | 81 | -0.12 | 0.021 | N/A |
| hsa-miR-6766-5p | 3999-4005 | 7mer-1A | -0.19 | 75 | -0.19 | 0 | N/A |
| hsa-miR-6748-5p | 4000-4006 | 7mer-m8 | -0.09 | 50 | -0.09 | 0 | N/A |
| hsa-miR-6769b-5p | 4001-4007 | 7mer-m8 | -0.08 | 68 | -0.08 | 0 | N/A |
| hsa-miR-6769a-5p | 4001-4007 | 7mer-m8 | -0.07 | 61 | -0.07 | 0 | N/A |
| hsa-miR-6134 | 4003-4009 | 7mer-m8 | -0.07 | 53 | -0.07 | 0.021 | N/A |
| hsa-miR-6089 | 4009-4015 | 7mer-m8 | -0.13 | 90 | -0.13 | 0 | N/A |
| hsa-miR-6799-5p | 4012-4018 | 7mer-1A | -0.04 | 71 | -0.04 | 0 | N/A |
| hsa-miR-6779-5p | 4012-4019 | 8mer | -0.11 | 66 | -0.11 | 0.072 | N/A |
| hsa-miR-3689a-3p | 4012-4019 | 8mer | -0.07 | 60 | -0.07 | 0.072 | N/A |
| hsa-miR-30b-3p | 4012-4019 | 8mer | -0.07 | 59 | -0.07 | 0.072 | N/A |
| hsa-miR-3689b-3p | 4012-4019 | 8mer | -0.07 | 59 | -0.07 | 0.072 | N/A |
| hsa-miR-3689c | 4012-4019 | 8mer | -0.07 | 59 | -0.07 | 0.072 | N/A |
| hsa-miR-1273h-5p | 4012-4019 | 8mer | -0.08 | 57 | -0.08 | 0.072 | N/A |
| hsa-miR-6883-5p | 4012-4018 | 7mer-1A | -0.05 | 54 | -0.05 | 0 | N/A |
| hsa-miR-6780a-5p | 4012-4019 | 8mer | -0.05 | 51 | -0.05 | 0.072 | N/A |
| hsa-miR-149-3p | 4012-4018 | 7mer-1A | -0.05 | 50 | -0.05 | 0 | N/A |
| hsa-miR-7106-5p | 4012-4018 | 7mer-1A | -0.04 | 44 | -0.04 | 0 | N/A |
| hsa-miR-4728-5p | 4012-4018 | 7mer-1A | -0.03 | 39 | -0.03 | 0 | N/A |
| hsa-miR-6785-5p | 4012-4018 | 7mer-1A | -0.01 | 25 | -0.01 | 0 | N/A |
| hsa-miR-6788-5p | 4013-4019 | 7mer-1A | -0.05 | 55 | -0.05 | 0 | N/A |
| hsa-miR-887-5p | 4013-4020 | 8mer | -0.03 | 38 | -0.03 | 0 | N/A |
| hsa-miR-30c-1-3p | 4013-4019 | 7mer-1A | -0.01 | 30 | -0.01 | 0 | N/A |
| hsa-miR-30c-2-3p | 4013-4019 | 7mer-1A | -0.01 | 27 | -0.01 | 0 | N/A |
| hsa-miR-3122 | 4014-4020 | 7mer-1A | -0.01 | 41 | -0.01 | 0.072 | N/A |
| hsa-miR-450a-1-3p | 4014-4020 | 7mer-1A | -0.02 | 37 | -0.02 | 0.072 | N/A |
| hsa-miR-3913-5p | 4014-4020 | 7mer-1A | -0.01 | 35 | -0.01 | 0.072 | N/A |
| hsa-miR-6513-5p | 4014-4020 | 7mer-1A | -0.01 | 25 | -0.01 | 0 | N/A |
| hsa-miR-383-3p | 4020-4026 | 7mer-m8 | -0.02 | 34 | -0.02 | 0 | N/A |
| hsa-miR-7977 | 4023-4029 | 7mer-m8 | -0.08 | 76 | -0.08 | 0 | N/A |
| hsa-miR-4284 | 4038-4045 | 8mer | -0.03 | 65 | -0.03 | 0 | N/A |
| hsa-miR-24-3p | 4039-4045 | 7mer-1A | -0.01 | 25 | -0.01 | 0.201 | < 0.1 |
| hsa-miR-6821-5p | 4040-4051 | non-canonical | N/A | N/A | N/A | 0 | N/A |
| hsa-miR-6821-5p | 4040-4051 | non-canonical | N/A | N/A | N/A | 0 | N/A |
| hsa-miR-1268b | 4047-4054 | 8mer | -0.29 | 65 | -0.29 | 0 | N/A |
| hsa-miR-1268a | 4047-4054 | 8mer | -0.29 | 65 | -0.29 | 0 | N/A |
| hsa-miR-585-3p | 4048-4054 | 7mer-1A | -0.11 | 40 | -0.11 | 0 | N/A |
| hsa-miR-1228-5p | 4049-4055 | 7mer-1A | -0.12 | 70 | -0.12 | 0 | N/A |
| hsa-miR-3147 | 4050-4056 | 7mer-m8 | -0.02 | 44 | -0.02 | 0 | N/A |
| hsa-miR-4259 | 4051-4057 | 7mer-m8 | -0.02 | 33 | -0.02 | 0 | N/A |
| hsa-miR-4281 | 4058-4064 | 7mer-1A | -0.13 | 85 | -0.13 | 0 | N/A |
| hsa-miR-133a-3p.1 | 4058-4064 | 7mer-m8 | -0.14 | 69 | -0.14 | 0.128 | < 0.1 |
| hsa-miR-7152-3p | 4059-4065 | 7mer-m8 | -0.15 | 83 | -0.15 | 0 | N/A |
| hsa-miR-504-5p.1 | 4062-4068 | 7mer-m8 | -0.13 | 88 | -0.13 | 0.201 | N/A |
| hsa-miR-6732-3p | 4063-4069 | 7mer-m8 | -0.12 | 75 | -0.12 | 0 | N/A |
| hsa-miR-548as-3p | 4065-4071 | 7mer-m8 | -0.02 | 64 | -0.02 | 0 | N/A |
| hsa-miR-3613-3p | 4075-4082 | 8mer | -0.03 | 62 | -0.03 | 0 | N/A |
| hsa-miR-6884-3p | 4080-4086 | 7mer-1A | -0.06 | 71 | -0.06 | 0 | N/A |
| hsa-miR-1306-5p | 4084-4091 | 8mer | -0.31 | 96 | -0.31 | 0.149 | < 0.1 |
| hsa-miR-5096 | 4087-4093 | 7mer-m8 | -0.02 | 65 | -0.02 | 0 | N/A |
| hsa-miR-4311 | 4094-4100 | 7mer-m8 | -0.02 | 52 | -0.02 | 0 | N/A |
| hsa-miR-520d-5p | 4097-4104 | 8mer | -0.03 | 80 | -0.03 | 0.072 | N/A |
| hsa-miR-524-5p | 4097-4104 | 8mer | -0.03 | 78 | -0.03 | 0.072 | N/A |
| hsa-miR-8056 | 4103-4109 | 7mer-m8 | -0.17 | 82 | -0.17 | 0 | N/A |
| hsa-miR-3681-5p | 4104-4111 | 8mer | -0.3 | 97 | -0.3 | 0 | N/A |
| hsa-miR-6849-5p | 4104-4111 | 8mer | -0.26 | 97 | -0.26 | 0 | N/A |
| hsa-miR-570-3p | 4113-4119 | 7mer-1A | -0.06 | 69 | -0.06 | 0 | N/A |
| hsa-miR-8069 | 4126-4132 | 7mer-m8 | -0.1 | 83 | -0.1 | 0 | N/A |
| hsa-miR-4426 | 4128-4135 | 8mer | -0.12 | 93 | -0.12 | 0 | N/A |
| hsa-miR-4662b | 4128-4135 | 8mer | -0.19 | 93 | -0.19 | 0 | N/A |
| hsa-miR-4647 | 4128-4135 | 8mer | -0.1 | 91 | -0.1 | 0 | N/A |
| hsa-miR-4742-5p | 4136-4142 | 7mer-1A | -0.22 | 96 | -0.22 | 0.021 | N/A |
| hsa-miR-4514 | 4136-4142 | 7mer-1A | -0.22 | 94 | -0.22 | 0 | N/A |
| hsa-miR-4692 | 4136-4142 | 7mer-1A | -0.2 | 93 | -0.2 | 0 | N/A |
| hsa-miR-6715b-5p | 4136-4142 | 7mer-1A | -0.14 | 89 | -0.14 | 0 | N/A |
| hsa-miR-4269 | 4136-4142 | 7mer-1A | -0.01 | 54 | -0.01 | 0 | N/A |
| hsa-miR-633 | 4142-4149 | 8mer | -0.03 | 57 | -0.03 | 0 | N/A |
| hsa-miR-4464 | 4148-4154 | 7mer-1A | -0.16 | 90 | -0.16 | 0 | N/A |
| hsa-miR-4748 | 4148-4154 | 7mer-1A | -0.09 | 88 | -0.09 | 0 | N/A |
| hsa-miR-329-5p | 4148-4154 | 7mer-1A | -0.02 | 51 | -0.02 | 0.072 | N/A |
| hsa-miR-6507-5p | 4153-4159 | 7mer-m8 | -0.02 | 59 | -0.02 | 0 | N/A |
| hsa-miR-3185 | 4155-4161 | 7mer-1A | -0.06 | 74 | -0.06 | 0 | N/A |
| hsa-miR-581 | 4165-4172 | 8mer | -0.29 | 96 | -0.29 | 0.448 | N/A |
| hsa-miR-578 | 4167-4173 | 7mer-m8 | -0.02 | 59 | -0.02 | 0.445 | N/A |
| hsa-miR-4677-5p | 4169-4175 | 7mer-m8 | -0.07 | 76 | -0.07 | 0.072 | N/A |
| hsa-miR-2116-5p | 4169-4175 | 7mer-1A | -0.03 | 60 | -0.03 | 0 | N/A |
| hsa-miR-22-5p | 4169-4175 | 7mer-1A | -0.01 | 37 | -0.01 | 0 | N/A |
| hsa-miR-4255 | 4171-4177 | 7mer-m8 | -0.21 | 94 | -0.21 | 0 | N/A |
| hsa-miR-548ah-5p | 4174-4180 | 7mer-1A | -0.14 | 93 | -0.14 | 0 | N/A |
| hsa-miR-519d-3p | 4174-4180 | 7mer-1A | -0.04 | 80 | -0.04 | 0.179 | < 0.1 |
| hsa-miR-3609 | 4174-4180 | 7mer-1A | -0.06 | 80 | -0.06 | 0 | N/A |
| hsa-miR-93-5p | 4174-4180 | 7mer-1A | -0.03 | 78 | -0.03 | 0.179 | < 0.1 |
| hsa-miR-17-5p | 4174-4180 | 7mer-1A | -0.03 | 77 | -0.03 | 0.179 | < 0.1 |
| hsa-miR-20b-5p | 4174-4180 | 7mer-1A | -0.03 | 77 | -0.03 | 0.179 | < 0.1 |
| hsa-miR-106a-5p | 4174-4180 | 7mer-1A | -0.03 | 77 | -0.03 | 0.179 | < 0.1 |
| hsa-miR-4796-3p | 4174-4180 | 7mer-1A | -0.03 | 74 | -0.03 | 0 | N/A |
| hsa-miR-526b-3p | 4174-4180 | 7mer-1A | -0.01 | 49 | -0.01 | 0.179 | < 0.1 |
| hsa-miR-20a-5p | 4174-4180 | 7mer-1A | -0.01 | 46 | -0.01 | 0.179 | < 0.1 |
| hsa-miR-106b-5p | 4174-4180 | 7mer-1A | -0.01 | 46 | -0.01 | 0.179 | < 0.1 |
| hsa-miR-8075 | 4205-4211 | 7mer-m8 | -0.21 | 86 | -0.21 | 0 | N/A |
| hsa-miR-2682-5p | 4211-4217 | 7mer-1A | -0.25 | 97 | -0.25 | 0 | N/A |
| hsa-miR-34b-5p | 4211-4217 | 7mer-1A | -0.22 | 96 | -0.22 | 0 | N/A |
| hsa-miR-449c-5p | 4211-4217 | 7mer-1A | -0.19 | 95 | -0.19 | 0 | N/A |
| hsa-miR-1910-3p | 4211-4217 | 7mer-1A | -0.09 | 90 | -0.09 | 0 | N/A |
| hsa-miR-6893-5p | 4211-4217 | 7mer-1A | -0.16 | 89 | -0.16 | 0.075 | N/A |
| hsa-miR-6808-5p | 4211-4217 | 7mer-1A | -0.16 | 89 | -0.16 | 0.075 | N/A |
| hsa-miR-6511a-5p | 4211-4217 | 7mer-1A | -0.12 | 88 | -0.12 | 0 | N/A |
| hsa-miR-940 | 4211-4217 | 7mer-1A | -0.15 | 88 | -0.15 | 0.075 | N/A |
| hsa-miR-154-3p | 4222-4228 | 7mer-1A | -0.01 | 39 | -0.01 | 0.060 | N/A |
| hsa-miR-487a-3p | 4222-4228 | 7mer-1A | -0.01 | 38 | -0.01 | 0.060 | N/A |
| hsa-miR-4494 | 4235-4241 | 7mer-m8 | -0.02 | 35 | -0.02 | 0 | N/A |
| hsa-miR-4771 | 4236-4243 | 8mer | -0.2 | 87 | -0.2 | 0 | N/A |
| hsa-miR-1273h-3p | 4237-4243 | 7mer-m8 | -0.13 | 88 | -0.13 | 0 | N/A |
| hsa-miR-3166 | 4237-4243 | 7mer-1A | -0.11 | 63 | -0.11 | 0 | N/A |
| hsa-miR-6507-5p | 4243-4249 | 7mer-1A | -0.01 | 45 | -0.01 | 0 | N/A |
| hsa-miR-3187-3p | 4249-4255 | 7mer-m8 | -0.22 | 96 | -0.22 | 0 | N/A |
| hsa-miR-4529-5p | 4249-4255 | 7mer-1A | -0.12 | 73 | -0.12 | 0 | N/A |
| hsa-miR-5197-5p | 4252-4258 | 7mer-1A | -0.1 | 72 | -0.1 | 0 | N/A |
| hsa-miR-194-5p | 4261-4267 | 7mer-1A | -0.09 | 72 | -0.09 | 1.22 | < 0.1 |
| hsa-miR-6809-5p | 4272-4278 | 7mer-m8 | -0.17 | 80 | -0.17 | 0 | N/A |
| hsa-miR-6866-5p | 4276-4283 | 8mer | -0.35 | 96 | -0.35 | 0 | N/A |
| hsa-miR-877-5p | 4277-4283 | 7mer-1A | -0.05 | 52 | -0.05 | 0.733 | N/A |
| hsa-miR-1303 | 4278-4284 | 7mer-1A | -0.01 | 14 | -0.01 | 0.021 | N/A |
| hsa-miR-345-5p | 4284-4290 | 7mer-1A | -0.01 | 44 | -0.01 | 0.349 | N/A |
| hsa-miR-433-3p | 4290-4297 | 8mer | -0.03 | 56 | -0.03 | 0.117 | N/A |
| hsa-miR-7154-5p | 4291-4297 | 7mer-1A | -0.07 | 70 | -0.07 | 0 | N/A |
| hsa-miR-205-5p | 4293-4299 | 7mer-m8 | -0.02 | 44 | -0.02 | 0.362 | < 0.1 |
| hsa-miR-5088-3p | 4295-4301 | 7mer-1A | -0.11 | 80 | -0.11 | 0 | N/A |
| hsa-miR-188-5p | 4296-4303 | 8mer | -0.17 | 90 | -0.17 | 0.373 | N/A |
| hsa-miR-6866-3p | 4296-4303 | 8mer | -0.1 | 85 | -0.1 | 0.373 | N/A |
| hsa-miR-4803 | 4325-4332 | 8mer | -0.03 | 62 | -0.03 | 0 | N/A |
| hsa-miR-4798-5p | 4331-4337 | 7mer-1A | -0.22 | 69 | -0.22 | 0 | N/A |
| hsa-miR-5705 | 4333-4340 | 8mer | -0.5 | 97 | -0.5 | 0 | N/A |
| hsa-miR-5010-3p | 4338-4345 | 8mer | -0.06 | 76 | -0.06 | 0.409 | N/A |
| hsa-miR-2113 | 4339-4345 | 7mer-1A | -0.01 | 42 | -0.01 | 0 | N/A |
| hsa-miR-4328 | 4342-4348 | 7mer-m8 | -0.02 | 47 | -0.02 | 0 | N/A |
| hsa-miR-548au-3p | 4344-4351 | 8mer | -0.31 | 96 | -0.31 | 0 | N/A |
| hsa-miR-449b-5p | 4345-4351 | 7mer-1A | -0.25 | 86 | -0.25 | 2.650 | 0.3 |
| hsa-miR-449a | 4345-4351 | 7mer-1A | -0.23 | 83 | -0.23 | 2.650 | 0.3 |
| hsa-miR-34c-5p | 4345-4351 | 7mer-1A | -0.22 | 80 | -0.22 | 2.650 | 0.3 |
| hsa-miR-34a-5p | 4345-4351 | 7mer-1A | -0.18 | 74 | -0.18 | 2.650 | 0.3 |
| hsa-miR-7150 | 4346-4352 | 7mer-1A | -0.25 | 93 | -0.25 | 0 | N/A |
| hsa-miR-96-5p | 4347-4353 | 7mer-1A | -0.02 | 47 | -0.02 | 2.342 | < 0.1 |
| hsa-miR-182-5p | 4347-4353 | 7mer-1A | -0.05 | 47 | -0.05 | 2.342 | < 0.1 |
| hsa-miR-1271-5p | 4347-4353 | 7mer-1A | -0.02 | 43 | -0.02 | 2.342 | < 0.1 |
| hsa-miR-922 | 4361-4367 | 7mer-m8 | -0.09 | 89 | -0.09 | 0.075 | N/A |
| hsa-miR-181c-5p | 4371-4377 | 7mer-m8 | -0.08 | 74 | -0.08 | 2.268 | < 0.1 |
| hsa-miR-4262 | 4371-4377 | 7mer-m8 | -0.03 | 71 | -0.03 | 2.268 | < 0.1 |
| hsa-miR-181a-5p | 4371-4377 | 7mer-m8 | -0.07 | 70 | -0.07 | 2.268 | < 0.1 |
| hsa-miR-181b-5p | 4371-4377 | 7mer-m8 | -0.06 | 67 | -0.06 | 2.268 | < 0.1 |
| hsa-miR-181d-5p | 4371-4377 | 7mer-m8 | -0.06 | 67 | -0.06 | 2.268 | < 0.1 |
| hsa-miR-543 | 4372-4378 | 7mer-m8 | -0.09 | 92 | -0.09 | 2.620 | N/A |
| hsa-miR-30b-5p | 4375-4381 | 7mer-m8 | -0.1 | 62 | -0.1 | 2.466 | 0.22 |
| hsa-miR-30c-5p | 4375-4381 | 7mer-m8 | -0.1 | 62 | -0.1 | 2.466 | 0.22 |
| hsa-miR-30d-5p | 4375-4381 | 7mer-m8 | -0.08 | 56 | -0.08 | 2.466 | 0.22 |
| hsa-miR-30a-5p | 4375-4381 | 7mer-m8 | -0.08 | 56 | -0.08 | 2.466 | 0.22 |
| hsa-miR-30e-5p | 4375-4381 | 7mer-m8 | -0.08 | 54 | -0.08 | 2.466 | 0.22 |
| hsa-miR-8069 | 4380-4386 | 7mer-1A | -0.02 | 45 | -0.02 | 0 | N/A |
| hsa-miR-8075 | 4382-4389 | 8mer | -0.26 | 92 | -0.26 | 0 | N/A |
| hsa-miR-4728-3p | 4384-4390 | 7mer-m8 | -0.11 | 80 | -0.11 | 0 | N/A |
| hsa-miR-155-5p | 4386-4392 | 7mer-m8 | -0.16 | 83 | -0.16 | 2.126 | 0.23 |
| hsa-miR-5692a | 4389-4395 | 7mer-m8 | -0.02 | 73 | -0.02 | 0 | N/A |
| hsa-miR-3163 | 4392-4398 | 7mer-m8 | -0.02 | 65 | -0.02 | 0 | N/A |
| hsa-miR-1252-3p | 4398-4404 | 7mer-1A | -0.05 | 86 | -0.05 | 0 | N/A |
| hsa-miR-3646 | 4398-4404 | 7mer-1A | -0.02 | 47 | -0.02 | 0 | N/A |
| hsa-miR-3663-3p | 4409-4415 | 7mer-1A | -0.07 | 58 | -0.07 | 0 | N/A |
| hsa-miR-4276 | 4413-4419 | 7mer-m8 | -0.12 | 76 | -0.12 | 0 | N/A |
| hsa-miR-5190 | 4413-4419 | 7mer-1A | -0.01 | 53 | -0.01 | 0 | N/A |
| hsa-miR-5094 | 4414-4420 | 7mer-m8 | -0.15 | 85 | -0.15 | 0 | N/A |
| hsa-miR-582-5p | 4425-4431 | 7mer-m8 | -0.02 | 42 | -0.02 | 0.058 | N/A |
| hsa-miR-139-5p | 4426-4432 | 7mer-m8 | -0.06 | 73 | -0.06 | 0.058 | < 0.1 |
| hsa-miR-548v | 4427-4433 | 7mer-m8 | -0.15 | 75 | -0.15 | 0 | N/A |
| hsa-miR-127-5p | 4431-4437 | 7mer-m8 | -0.02 | 35 | -0.02 | 0 | N/A |
| hsa-miR-3928-5p | 4431-4437 | 7mer-1A | -0.01 | 34 | -0.01 | 0.072 | N/A |
| hsa-miR-6806-3p | 4431-4437 | 7mer-1A | -0.01 | 33 | -0.01 | 0.072 | N/A |
| hsa-miR-564 | 4441-4447 | 7mer-1A | -0.18 | 64 | -0.18 | 0.021 | N/A |
| hsa-miR-183-5p.2 | 4442-4448 | 7mer-1A | -0.09 | 68 | -0.09 | 0.201 | < 0.1 |
| hsa-miR-1271-5p | 4442-4448 | 7mer-m8 | -0.04 | 56 | -0.04 | 0.201 | < 0.1 |
| hsa-miR-96-5p | 4442-4448 | 7mer-m8 | -0.02 | 43 | -0.02 | 0.201 | < 0.1 |
| hsa-miR-224-5p | 4454-4460 | 7mer-1A | -0.02 | 59 | -0.02 | 0.841 | N/A |
| hsa-miR-4477a | 4457-4463 | 7mer-m8 | -0.02 | 64 | -0.02 | 0 | N/A |
| hsa-miR-513b-5p | 4471-4478 | 8mer | -0.12 | 88 | -0.12 | 0.075 | N/A |
| hsa-miR-4457 | 4471-4477 | 7mer-1A | -0.03 | 53 | -0.03 | 0 | N/A |
| hsa-miR-125b-2-3p | 4471-4477 | 7mer-1A | -0.02 | 48 | -0.02 | 0 | N/A |
| hsa-miR-7-2-3p | 4478-4484 | 7mer-m8 | -0.02 | 72 | -0.02 | 0 | N/A |
| hsa-miR-7-1-3p | 4478-4484 | 7mer-m8 | -0.02 | 72 | -0.02 | 0 | N/A |
| hsa-miR-7112-3p | 4488-4494 | 7mer-m8 | -0.08 | 70 | -0.08 | 0 | N/A |
| hsa-miR-4642 | 4490-4497 | 8mer | -0.25 | 94 | -0.25 | 0 | N/A |
| hsa-miR-570-3p | 4509-4515 | 7mer-m8 | -0.03 | 47 | -0.03 | 0 | N/A |
| hsa-miR-186-5p | 4513-4519 | 7mer-1A | -0.02 | 73 | -0.02 | 1.364 | N/A |
| hsa-miR-3133 | 4513-4519 | 7mer-1A | -0.01 | 63 | -0.01 | 0 | N/A |
| hsa-miR-4744 | 4514-4520 | 7mer-m8 | -0.13 | 88 | -0.13 | 0 | N/A |
| hsa-miR-603 | 4519-4525 | 7mer-m8 | -0.07 | 86 | -0.07 | 0.072 | N/A |
| hsa-miR-8485 | 4521-4527 | 7mer-m8 | -0.02 | 19 | -0.02 | 0 | N/A |
| hsa-miR-3591-3p | 4537-4543 | 7mer-1A | -0.21 | 91 | -0.21 | 0.072 | N/A |
| hsa-miR-21-3p | 4537-4543 | 7mer-1A | -0.13 | 80 | -0.13 | 0.072 | N/A |
| hsa-miR-302c-5p | 4539-4545 | 7mer-1A | -0.01 | 35 | -0.01 | 0 | N/A |
| hsa-miR-4676-3p | 4543-4549 | 7mer-m8 | -0.08 | 77 | -0.08 | 0.031 | N/A |
| hsa-miR-892c-3p | 4543-4549 | 7mer-m8 | -0.08 | 76 | -0.08 | 0.031 | N/A |
| hsa-miR-452-5p | 4543-4549 | 7mer-m8 | -0.04 | 60 | -0.04 | 0.031 | N/A |
| hsa-miR-548g-3p | 4545-4551 | 7mer-m8 | -0.07 | 84 | -0.07 | 0 | N/A |
| hsa-miR-548ar-3p | 4546-4552 | 7mer-m8 | -0.04 | 83 | -0.04 | 0.021 | N/A |
| hsa-miR-548az-3p | 4546-4552 | 7mer-m8 | -0.04 | 82 | -0.04 | 0.021 | N/A |
| hsa-miR-548f-3p | 4546-4552 | 7mer-m8 | -0.03 | 74 | -0.03 | 0.021 | N/A |
| hsa-miR-548a-3p | 4546-4552 | 7mer-m8 | -0.02 | 74 | -0.02 | 0.021 | N/A |
| hsa-miR-548e-3p | 4546-4552 | 7mer-m8 | -0.02 | 59 | -0.02 | 0.021 | N/A |
| hsa-miR-548ah-3p | 4547-4553 | 7mer-m8 | -0.02 | 67 | -0.02 | 0 | N/A |
| hsa-miR-548aq-3p | 4547-4553 | 7mer-m8 | -0.02 | 67 | -0.02 | 0 | N/A |
| hsa-miR-548x-3p | 4547-4553 | 7mer-m8 | -0.02 | 67 | -0.02 | 0 | N/A |
| hsa-miR-548ae-3p | 4547-4553 | 7mer-m8 | -0.02 | 67 | -0.02 | 0 | N/A |
| hsa-miR-548aj-3p | 4547-4553 | 7mer-m8 | -0.02 | 67 | -0.02 | 0 | N/A |
| hsa-miR-548am-3p | 4547-4553 | 7mer-m8 | -0.02 | 67 | -0.02 | 0 | N/A |
| hsa-miR-548j-3p | 4547-4553 | 7mer-m8 | -0.02 | 67 | -0.02 | 0 | N/A |
| hsa-miR-3123 | 4552-4558 | 7mer-1A | -0.12 | 94 | -0.12 | 0 | N/A |
| hsa-miR-3925-5p | 4552-4558 | 7mer-1A | -0.01 | 54 | -0.01 | 0 | N/A |
| hsa-miR-3976 | 4553-4559 | 7mer-m8 | -0.15 | 80 | -0.15 | 0 | N/A |
| hsa-miR-3121-3p | 4555-4561 | 7mer-m8 | -0.02 | 48 | -0.02 | 0.021 | N/A |
| hsa-miR-4328 | 4563-4569 | 7mer-1A | -0.01 | 43 | -0.01 | 0 | N/A |
| hsa-miR-656-3p | 4580-4586 | 7mer-1A | -0.01 | 39 | -0.01 | 0.349 | N/A |
| hsa-miR-1277-5p | 4582-4588 | 7mer-m8 | -0.02 | 53 | -0.02 | 0 | N/A |
| hsa-miR-425-3p | 4587-4594 | 8mer | -0.52 | 98 | -0.52 | 0 | N/A |
| hsa-miR-6771-5p | 4588-4594 | 7mer-1A | -0.22 | 72 | -0.22 | 0 | N/A |
| hsa-miR-1236-3p | 4592-4598 | 7mer-1A | -0.01 | 32 | -0.01 | 0.021 | N/A |
| hsa-miR-6515-3p | 4592-4598 | 7mer-1A | -0.02 | 32 | -0.02 | 0 | N/A |
| hsa-miR-3675-3p | 4594-4600 | 7mer-1A | -0.12 | 87 | -0.12 | 0 | N/A |
| hsa-miR-5580-3p | 4598-4604 | 7mer-1A | -0.01 | 43 | -0.01 | 0 | N/A |
| hsa-miR-4699-3p | 4602-4608 | 7mer-1A | -0.01 | 32 | -0.01 | 0 | N/A |
| hsa-miR-21-5p | 4606-4612 | 7mer-m8 | -0.09 | 76 | -0.09 | 2.639 | < 0.1 |
| hsa-miR-590-5p | 4606-4612 | 7mer-m8 | -0.05 | 73 | -0.05 | 2.639 | < 0.1 |
| hsa-miR-27b-5p | 4607-4613 | 7mer-m8 | -0.15 | 80 | -0.15 | 0.505 | N/A |
| hsa-miR-6761-5p | 4611-4618 | 8mer | -0.13 | 88 | -0.13 | 0.609 | N/A |
| hsa-miR-4251 | 4612-4618 | 7mer-1A | -0.06 | 70 | -0.06 | 0 | N/A |
| hsa-miR-4329 | 4612-4618 | 7mer-1A | -0.01 | 45 | -0.01 | 0 | N/A |
| hsa-miR-4653-5p | 4613-4619 | 7mer-m8 | -0.18 | 92 | -0.18 | 0.310 | N/A |
| hsa-miR-3921 | 4613-4619 | 7mer-m8 | -0.15 | 90 | -0.15 | 0.310 | N/A |
| hsa-miR-4714-5p | 4614-4620 | 7mer-m8 | -0.11 | 79 | -0.11 | 0.409 | N/A |
| hsa-miR-7-1-3p | 4631-4637 | 7mer-m8 | -0.02 | 72 | -0.02 | 0 | N/A |
| hsa-miR-7-2-3p | 4631-4637 | 7mer-m8 | -0.02 | 72 | -0.02 | 0 | N/A |
| hsa-miR-3065-5p | 4632-4639 | 8mer | -0.03 | 43 | -0.03 | 0 | N/A |
| hsa-miR-7159-5p | 4633-4639 | 7mer-m8 | -0.02 | 32 | -0.02 | 0 | N/A |
| hsa-miR-4708-3p | 4640-4646 | 7mer-m8 | -0.15 | 80 | -0.15 | 0.273 | N/A |
| hsa-miR-4530 | 4644-4650 | 7mer-1A | -0.18 | 91 | -0.18 | 0 | N/A |
| hsa-miR-338-3p | 4644-4650 | 7mer-1A | -0.02 | 54 | -0.02 | 0.350 | < 0.1 |
| hsa-miR-198 | 4646-4652 | 7mer-1A | -0.05 | 55 | -0.05 | 0.072 | N/A |
| hsa-miR-3912-5p | 4647-4653 | 7mer-1A | -0.01 | 23 | -0.01 | 0 | N/A |
| hsa-miR-6500-3p | 4650-4656 | 7mer-m8 | -0.02 | 44 | -0.02 | 0 | N/A |
| hsa-miR-1255b-2-3p | 4652-4658 | 7mer-m8 | -0.02 | 35 | -0.02 | 0 | N/A |
| hsa-miR-497-3p | 4654-4660 | 7mer-m8 | -0.02 | 68 | -0.02 | 0.072 | N/A |
| hsa-miR-6715a-3p | 4655-4661 | 7mer-m8 | -0.02 | 25 | -0.02 | 0 | N/A |
| hsa-miR-8064 | 4665-4671 | 7mer-1A | -0.1 | 76 | -0.1 | 0 | N/A |
| hsa-miR-557 | 4667-4673 | 7mer-m8 | -0.02 | 76 | -0.02 | 0.075 | N/A |
| hsa-miR-507 | 4667-4673 | 7mer-m8 | -0.02 | 65 | -0.02 | 0.075 | N/A |
| hsa-miR-5087 | 4670-4676 | 7mer-1A | -0.01 | 28 | -0.01 | 0 | N/A |
| hsa-miR-29b-2-5p | 4671-4677 | 7mer-1A | -0.01 | 40 | -0.01 | 0.468 | N/A |
| hsa-miR-659-3p | 4672-4678 | 7mer-1A | -0.01 | 37 | -0.01 | 0 | N/A |
| hsa-miR-561-3p | 4676-4682 | 7mer-m8 | -0.02 | 54 | -0.02 | 0 | N/A |
| hsa-miR-186-5p | 4681-4687 | 7mer-1A | -0.02 | 72 | -0.02 | 0.179 | N/A |
| hsa-miR-3133 | 4681-4687 | 7mer-1A | -0.01 | 63 | -0.01 | 0 | N/A |
| hsa-miR-555 | 4685-4691 | 7mer-1A | -0.15 | 68 | -0.15 | 0.021 | N/A |
| hsa-miR-6861-5p | 4686-4692 | 7mer-m8 | -0.18 | 88 | -0.18 | 0 | N/A |
| hsa-miR-4325 | 4691-4697 | 7mer-1A | -0.09 | 81 | -0.09 | 0 | N/A |
| hsa-miR-7703 | 4691-4697 | 7mer-1A | -0.01 | 30 | -0.01 | 0 | N/A |
| hsa-miR-4762-5p | 4699-4705 | 7mer-m8 | -0.06 | 75 | -0.06 | 0 | N/A |
| hsa-miR-7-1-3p | 4701-4707 | 7mer-m8 | -0.02 | 72 | -0.02 | 0 | N/A |
| hsa-miR-7-2-3p | 4701-4707 | 7mer-m8 | -0.02 | 72 | -0.02 | 0 | N/A |
| hsa-miR-4274 | 4709-4715 | 7mer-m8 | -0.28 | 96 | -0.28 | 0 | N/A |
| hsa-miR-4801 | 4714-4720 | 7mer-m8 | -0.07 | 82 | -0.07 | 0 | N/A |
| hsa-miR-4731-3p | 4714-4720 | 7mer-m8 | -0.03 | 73 | -0.03 | 0 | N/A |
| hsa-miR-4470 | 4734-4740 | 7mer-m8 | -0.22 | 95 | -0.22 | 0 | N/A |
| hsa-miR-548an | 4737-4743 | 7mer-m8 | -0.16 | 88 | -0.16 | 0 | N/A |
| hsa-miR-4799-5p | 4742-4748 | 7mer-1A | -0.04 | 81 | -0.04 | 0 | N/A |
| hsa-miR-576-5p | 4743-4749 | 7mer-1A | -0.03 | 81 | -0.03 | 0.072 | N/A |
| hsa-miR-4263 | 4743-4749 | 7mer-1A | -0.02 | 58 | -0.02 | 0 | N/A |
| hsa-miR-4699-3p | 4754-4760 | 7mer-1A | -0.01 | 32 | -0.01 | 0 | N/A |
| hsa-miR-548c-3p | 4771-4777 | 7mer-1A | -0.01 | 57 | -0.01 | 0.312 | N/A |
| hsa-miR-548c-3p | 4777-4783 | 7mer-1A | -0.01 | 57 | 0 | 0.312 | N/A |

P_CT,_ the probability of conserved targeting; N/A, not applicable.
